# Naturally occurring modified ribonucleosides

**DOI:** 10.1002/wrna.1595

**Published:** 2020-04-16

**Authors:** Phillip J. McCown, Agnieszka Ruszkowska, Charlotte N. Kunkler, Kurtis Breger, Jacob P. Hulewicz, Matthew C. Wang, Noah A. Springer, Jessica A. Brown

**Affiliations:** Department of Chemistry and Biochemistry, University of Notre Dame, Notre Dame, Indiana

**Keywords:** mRNA, ncRNA, RNA modification, rRNA, tRNA

## Abstract

The chemical identity of RNA molecules beyond the four standard ribonucleosides has fascinated scientists since pseudouridine was characterized as the “fifth” ribonucleotide in 1951. Since then, the ever-increasing number and complexity of modified ribonucleosides have been found in viruses and throughout all three domains of life. Such modifications can be as simple as methylations, hydroxylations, or thiolations, complex as ring closures, glycosylations, acylations, or aminoacylations, or unusual as the incorporation of selenium. While initially found in transfer and ribosomal RNAs, modifications also exist in messenger RNAs and noncoding RNAs. Modifications have profound cellular outcomes at various levels, such as altering RNA structure or being essential for cell survival or organism viability. The aberrant presence or absence of RNA modifications can lead to human disease, ranging from cancer to various metabolic and developmental illnesses such as Hoyeraal–Hreidarsson syndrome, Bowen–Conradi syndrome, or Williams–Beuren syndrome. In this review article, we summarize the characterization of all 143 currently known modified ribonucleosides by describing their taxonomic distributions, the enzymes that generate the modifications, and any implications in cellular processes, RNA structure, and disease. We also highlight areas of active research, such as specific RNAs that contain a particular type of modification as well as methodologies used to identify novel RNA modifications.

## 1 | INTRODUCTION

**FIGURE 1:**
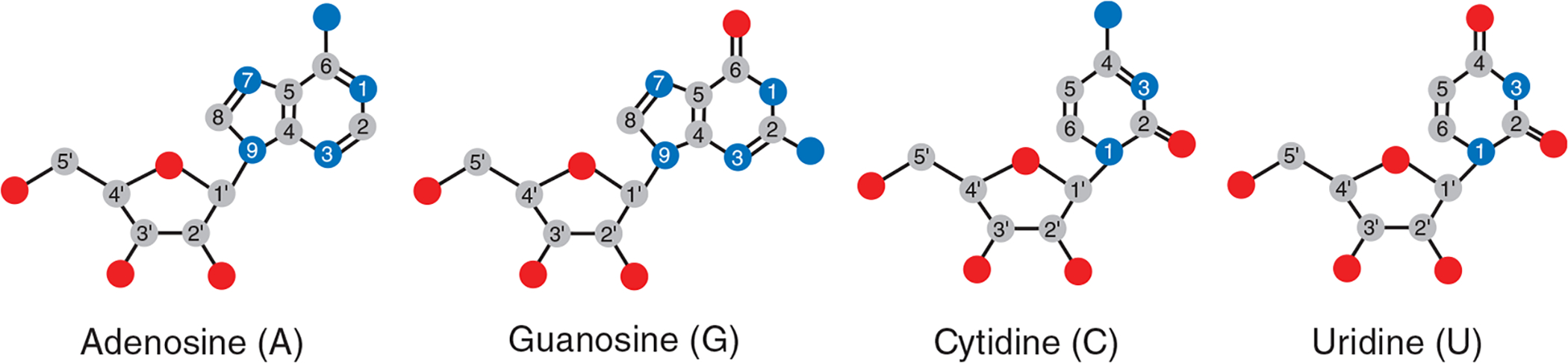
Ribonucleoside numbering of adenine, guanine, cytosine, uracil bases and numbering of the ribose sugar for adenosine (A), guanosine (G), cytidine (C), and uridine (U). Carbon atoms are in gray, oxygen atoms are in red, and nitrogen atoms are in blue. Single lines between atoms depict single bonds, double lines between atoms depict double bonds, and hydrogens are not depicted for simplicity

Adenosine (A), guanosine (G), cytidine (C), and uridine (U) comprise the four ribonucleosides typically found in ribonucleic acid (RNA) molecules. These four ribonucleosides were divided into two categories based on the shape and chemical properties of the nucleobases: pyrimidines and purines. Pyrimidines, composed of C and U (represented as Y), feature a six-membered heterocyclic configuration, similar to pyridine. Purines, composed of A and G (represented as R), possess a heterocyclic configuration of a pyrimidine fused to an imidazole ring. The pyrimidines and purines can be added to ribose molecules, forming ribonucleosides (Figure 1). From these pyrimidines and purines, several derivatives such as thiamin pyrophosphate (TPP), flavin mononucleotide (FMN), and cyclic di-guanosine monophosphate (c-di-GMP) are thought to belong to the RNA world, a world in which genes, genomes, and enzymes were composed solely of RNA (Crick, 1968; Joyce & Szostak, 2018; Nelson & Breaker, 2017; Orgel, 1968; Robertson & Joyce, 2012; Woese, 1967). However, pyrimidines and purines have also been discovered to be the precursors for numerous modifications (Cohn & Volkin, 1951; Dunn, 1960; Hall, 1963; Holley, 1965; Iwanami & Brown, 1968; D. A. Smith & Visser, 1965; reviewed in Boccaletto et al., 2018; Cantara et al., 2011). The discovery and characterization of RNA modifications underscore their biological significance and embody exciting strides throughout RNA biology (Boccaletto et al., 2018; Jung & Goldman, 2018; Shi, Wei, & He, 2019; Thapar et al., 2019).

**FIGURE 2:**
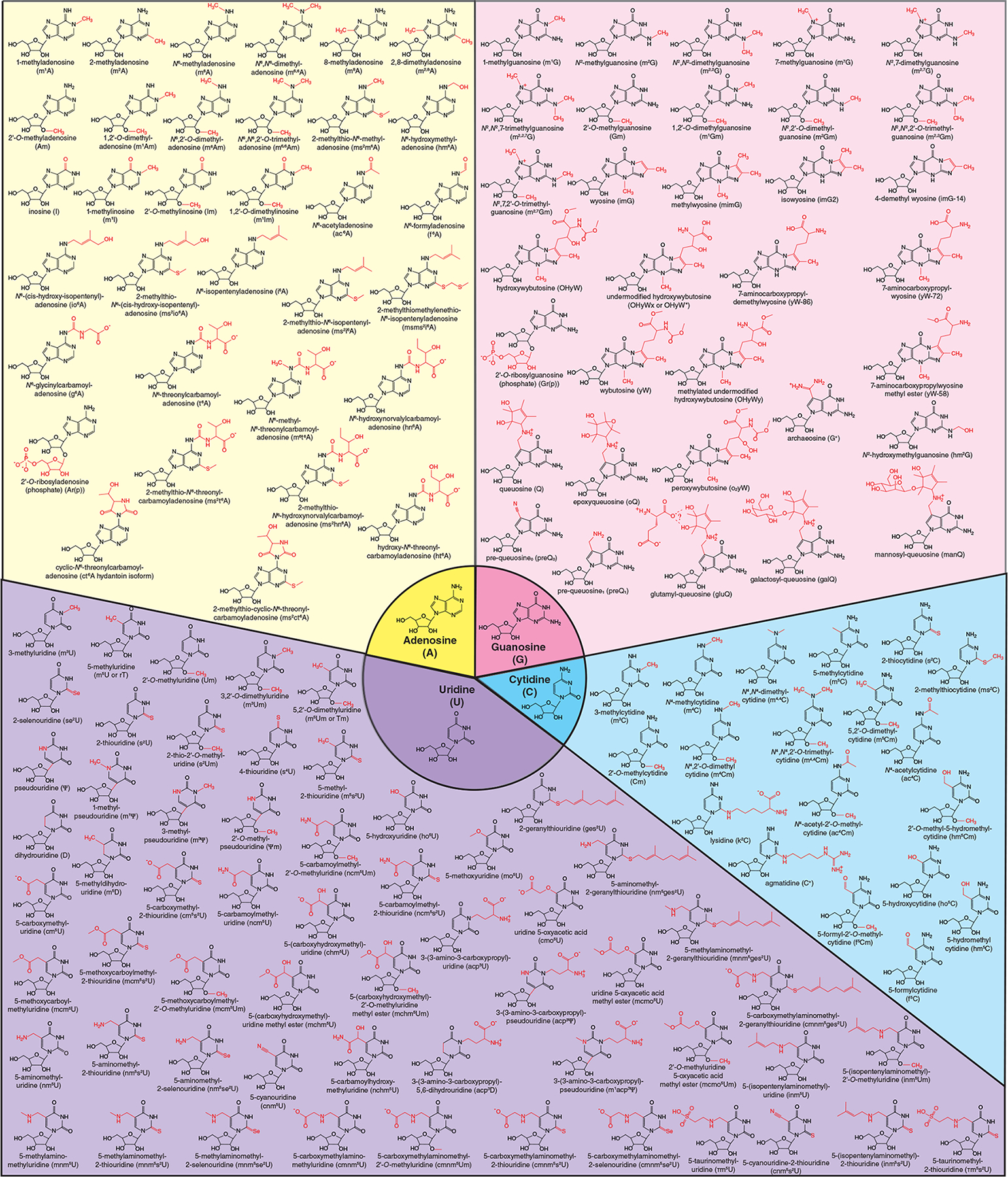
Chemical structures of all currently known RNA modifications. Adenosine-derived (yellow), guanosine-derived (pink), uridine-derived (purple), and cytidine-derived (cyan) modifications are classified based on the parent ribonucleoside. Red moieties indicate which portion of the modified ribonucleoside is different from the parent ribonucleoside, whose structures are shown in the central circle. A poster-size image is available as Figure S1

**FIGURE 3:**
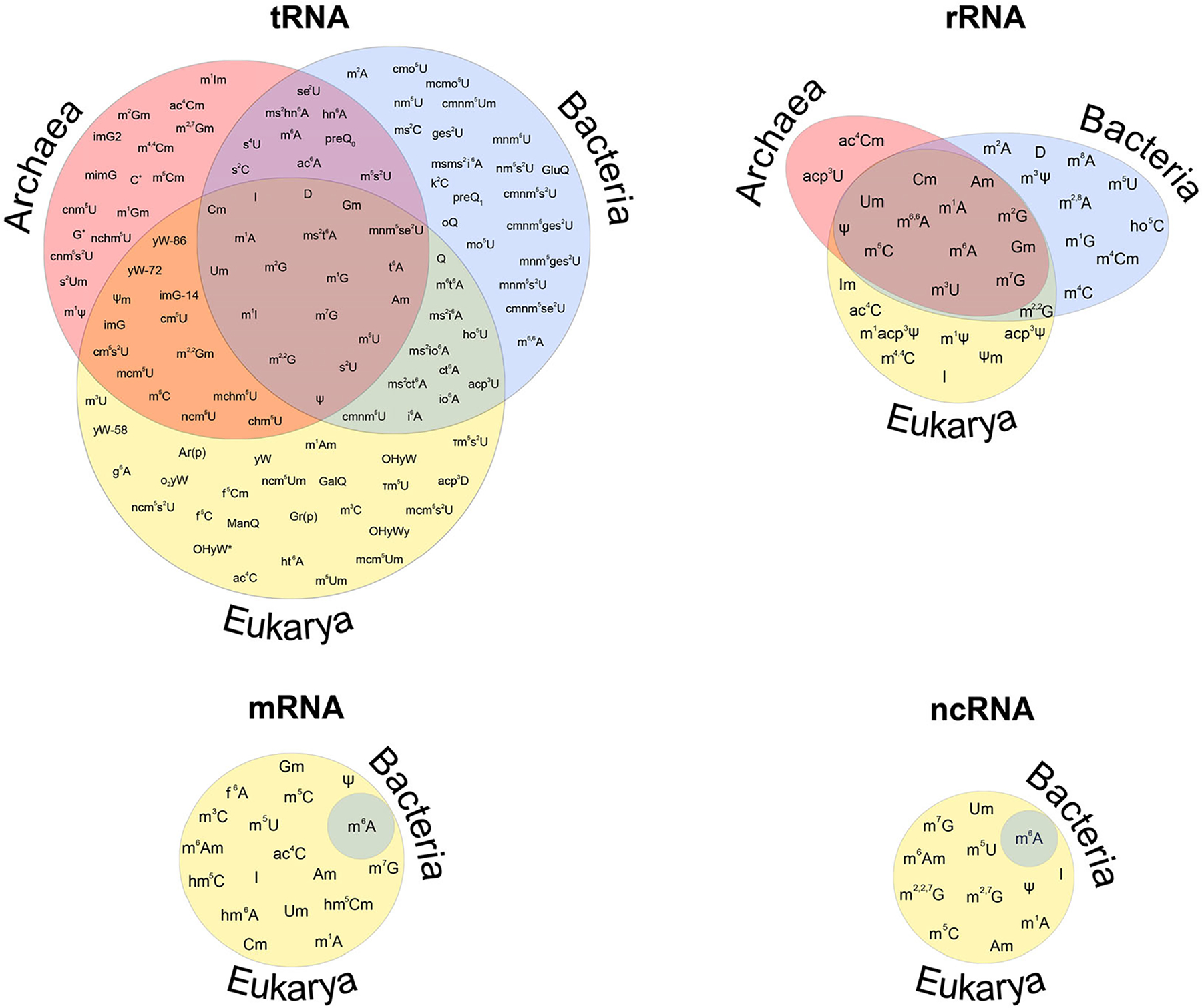
Euler diagrams showing the currently known phylogenetic distribution of ribonucleoside modifications in tRNA, rRNA, mRNA, and ncRNA classes. Archaeal modifications are in pink, bacterial modifications are in blue, and eukaryotic modifications are in yellow

**FIGURE 4:**
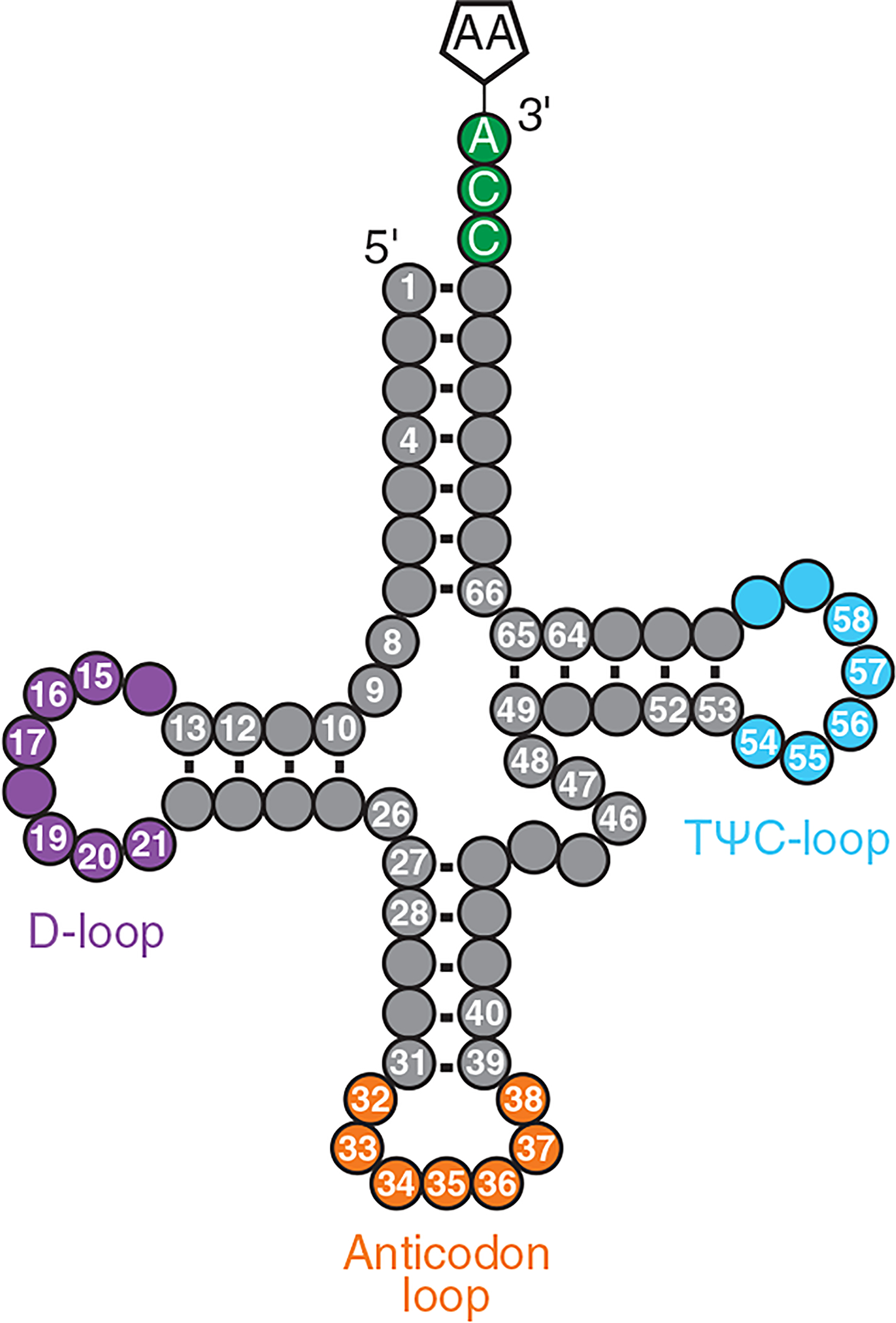
tRNA-specific modifications are clustered around specific nucleotides in tRNAs. A schematic of the secondary structure of tRNA is depicted as a cloverleaf structure, having a D-loop (purple), an anticodon loop (orange), a TΨC-loop (blue), and the 3′-CCA sequence (green) that is aminoacylated when charged with an amino acid (AA). Numbers refer to specific nucleotide positions where modifications have been mapped (Lorenz, Lünse, & Mörl, 2017; Phillips & de Crécy-Lagard, 2011; Phizicky & Hopper, 2010; Powell, Nicholls, & Minczuk, 2015). The modifications cataloged in Table 2 occur at numbered positions in Figure 4

Ribonucleoside modifications encompass a myriad of chemical moieties, which are added to adenosine, guanosine, cytidine, or uridine. From simple methylations or hydrations of double bonds to ring closures of the nitrogenous base or the addition of large groups such as amino acids or monosaccharides, almost all modifications are catalyzed by an enzyme. Some enzymes form multimers with auxiliary proteins (e.g., helper methyltransferases), expanding the range of applicable substrates or allowing for the integration of cellular activities and enzymatic processes (Guy & Phizicky, 2014). However, a few modifications arise from nonenzymatic processes or oxidative damage (Figure 2, Table 1). A further distinction pertains to RNA editing, which was originally described as a process of adding polyuridine residues within the coding region of select RNAs (Grosjean & Benne, 1998). RNA editing has since been expanded to include RNA base excisions or additions (e.g., deletion of uridine residues in pre-mRNAs or addition of G to select tRNAs) (Benne, 1994; Jackman & Phizicky, 2006) as well as RNA base conversions (e.g., A-to-I or C-to-U editing), although further distinctions have remained somewhat incoherent (Grosjean & Benne, 1998). While there are 111 modifications that can be found within transfer RNAs (tRNAs), 33 modifications can be found in ribosomal RNAs (rRNAs), 17 in messenger RNAs (mRNAs), and 11 in long noncoding RNAs (lncRNAs) and other noncoding RNAs (ncRNAs) (Figures 3 and 4, Table 2) (Boccaletto et al., 2018; Cantara et al., 2011; Lorenz et al., 2017).

In this review article, we summarize all currently known ribonucleoside modifications. Each section highlights one or more modifications that are structurally or functionally related to each other or belong to established biosynthetic pathways. Each section begins with the full chemical name of the ribonucleoside modification(s) and the corresponding IUPAC abbreviation(s), domain-of-life assignments, and RNA classes. Domain assignments refer to the domain-of-life of the host organisms that harbor a modification, using the abbreviations “A” for archaeal organisms, “B” for bacterial organisms, and “E” for eukaryotic organisms. Within the text, usage of the term “prokaryote” encompasses both archaeal and bacterial species (Woese & Fox, 1977). RNA modifications in different RNA classes are denoted as follows: a black cloverleaf (♣) for modifications in tRNAs, a red square (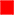) for modifications in rRNAs, a green dot (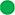) for modifications in mRNAs, and a blue triangle (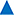) for modifications in ncRNAs. Instances where two or more modifications are grouped in a section but are found in different domains of life will be noted in parentheses. Next, we provide a description of which enzymes or pathways are involved in the synthesis of the modification. Also, we provide any additional data or discoveries associated with the modification, including impact on RNA structure, functional significance, disease relevance, or any other notable findings. We initially describe the pseudouridine (Ψ), inosine (I), and dihydrouridine (D) modifications for historical purposes, as these were among the first modifications to be found in RNA. We then characterize all adenosine-, guanosine-, cytidine-, and uridine-based modifications followed by ribose modifications. We conclude the review by summarizing the most recent technological advances in finding novel modifications, while also describing conflicting experimental evidence for the existence of select modifications.

## 2 | ABBREVIATIONS USED IN THIS WORK

Throughout this work, we use abbreviations that are commonplace or from publications that we reference. In addition to the ribonucleoside abbreviations that we catalog in Table 1, the following abbreviations appear in the text.

**Table:** 

ABH1:	alpha-ketoglutarate-dependent dioxygenase 1 or AlkB Homolog 1
ACP:	3-amino-3-carboxypropyl
ADAR:	adenosine deaminase acting on RNA
ADAT:	adenosine deaminase acting on tRNA
AHK3:	histidine kinase 3
AHK4:	histidine kinase 4
AID:	activation-induced cytidine deaminase
ALKB:	alpha-ketoglutarate-dependent dioxygenase
ALKBH1:	alkylated DNA repair protein (AlkB) homolog 1
ALKBH3:	alkylated DNA repair protein (AlkB) homolog 3
ALKBH5:	alkylated DNA repair protein (AlkB) homolog 5
ALKBH8:	alkylated DNA repair protein (AlkB) homolog 8
AML:	acute myeloid leukemia
ArcS/arcTGT:	archaeosine synthase
AroD:	3-dehydroquinate dehydratase
Atm1:	mitochondrial iron-sulfur clusters transporter
BMT5/6:	25S rRNA (uridine(2634)-*N*^3^)-methyltransferases 5 and 6
Bud23:	rRNA methyltransferase and ribosome maturation factor
c-di-GMP:	cyclic di-guanosine monophosphate
CDK5RAP1:	cyclin-dependent kinase 5 regulatory subunit associated protein 1
CDKAL1:	threonylcarbamoyladenosine tRNA methythiotransferase or cyclin-dependent kinase (CDK) 5-regulatory subunit associated protein 1 like 1
cDNA:	complementary DNA
CDS:	coding sequence
Cfr:	chloramphenicol-florfenicol resistance
CMC:	*N*-cyclohexyl-*N*′-(2-morpholinoethyl)-carbodiimide
CMCT:	*N*-cyclohexyl-*N*′-(2-morpholinoethyl)-carbodiimide metho-*p*-toluenesulphonate
c-myc:	cellular avian myelocytomatosis viral oncogene homolog
CmoABM:	carboxy-*S*-adenosyl-L-methionine synthase A, B, or M
COG1738:	conserved protein domain 1738
CRE1:	cytokinin response element 1
CsdAE:	cysteine desulfurase A or E
Ctu1–2:	cytoplasmic tRNA 2-thiolation proteins 1 or 2
DDX6:	DEAD-box helicase 6
Dim1:	dimethyladenosine transferase 1
DKC1:	dyskerin pseudouridine synthase 1
DNMT3A:	DNA methyltransferase 3 alpha or DNA (cytosine-5-)-methyltransferase 3 alpha
dpCoA:	3′-dephospho-coenzyme A
DROSHA:	double-stranded RNA-specific endoribonuclease
DTWD1–2:	DTW (aspartate-threonine-tryptophan) domain-containing proteins 1 or 2
Dus:	dihydrouridine synthase
Dus1–4p:	dihydrouridine synthase proteins 1–4
DusABC:	dihydrouridine synthase A, B, or C
eEF1A:	eukaryotic elongation factor 1A
EF-Tu:	elongation factor thermo unstable
EGFR:	epidermal growth factor receptor
eIF3H:	eukaryotic translation initiation factor 3 subunit H
eL41:	eukaryotic ribosomal protein L41
Elp1–6:	Elongator proteins 1–6
Emg1:	*N*^1^-specific pseudouridine methyltransferase
FICC-seq:	fluorouracil-induced-catalytic-crosslinking-sequencing
FMN:	flavin mononucleotide
FTO:	fat mass and obesity-associated related
Fur:	ferric uptake regulator
GAT-QueC:	glutamine amidotransferase class-II-7-cyano-7-deazaguanine synthase fusion protein
GCD:	tRNA (adenine-*N*^1^-)-methyltransferase
Gcd10p/Gcd14p:	tRNA (adenine-*N*^1^-)-methyltransferases 10 and 14 proteins or tRNA (adenine(58)-*N*(1))-methyltransferase noncatalytic subunit Trm6 and Trm61
GidA:	uridine 5-carboxymethylaminomethyl modification enzyme
GluQRS:	glutamyl-Q tRNA(Asp) synthetase
GRIA2:	glutamate ionotropic receptor alpha-amino 3-hydroxy-5-methyl-4-isoxazole propionate (AMPA) type subunit 2
GTPBP3:	guanosine triphosphate binding protein 3
H/ACA:	H- and ACA-box-containing small nucleolar RNA
HER2+:	human epidermal growth factor receptor 2 positive
hDus2p:	human dihydrouridine synthase protein 2
hnRNP:	heterogeneous nuclear ribonucleoprotein
hTRMT10A or B:	human tRNA methyltransferase 10 A or B
IGF2BP:	insulin-like growth factor 2 mRNA-binding protein
IPP:	isopentenyl pyrophosphate
IscS:	iron-sulfur cluster S or cysteine desulfurase
IscU:	iron-sulfur cluster assembly enzyme
Isu1/2:	iron-sulfur cluster assembly proteins 1 and 2
KEOPS:	kinase, putative endopeptidase and other proteins of small size
KsgA:	rRNA adenine dimethyltransferase or ribosomal RNA small subunit methyltransferase A
L7Ae:	50S ribosomal protein
LC-MS:	liquid chromatography-mass spectrometry
MALAT1:	metastasis-associated lung adenocarcinoma transcript 1
MAT2A:	methionine adenosyltransferase 2A
MELAS:	mitochondrial myopathy, encephalopathy, lactic acidosis, and stroke-like episodes
MERRF:	myoclonus epilepsy associated with ragged-red fibers
METTL:	methyltransferase-like protein
METTL2:	methyltransferase-like protein 2
METTL3/14:	methyltransferase-like proteins 3 and 14
METTL5:	methyltransferase-like protein 5
METTL6:	methyltransferase-like protein 6
METTL8:	methyltransferase-like protein 8
METTL15:	methyltransferase-like protein 15
METTL16:	methyltransferase-like protein 16
MiaA:	tRNA delta(2)-isopentenylpyrophosphate transferase A or tRNA dimethylallyltransferase A
MiaB:	tRNA delta(2)-isopentenylpyrophosphate transferase B or tRNA dimethylallyltransferase B
MiaE:	tRNA delta(2)-isopentenylpyrophosphate transferase E or tRNA dimethylallyltransferase E
miR/miRNA:	microRNA
MK2:	MAPK activated protein kinase 2 or mitogen-activated protein kinase-activated protein kinase 2 or MAPKAPK2
MLASA:	mitochondrial myopathy and sideroblastic anemia
MnmACEGH:	tRNA-specific 2-thiouridylase A, C, E, G, or H
MnmC(m):	tRNA-specific 2-thiouridylase C, methyltransferase domain
MnmC(o):	tRNA-specific 2-thiouridylase C, oxidoreductase domain
Moco:	molybdenum cofactor
Mod5:	tRNA isopentenyltransferase 1
MtaB:	threonylcarbamoyladenosine tRNA methylthiotransferase or TcdA
mTOR:	mammalian target of rapamycin
MTO1:	mitochondrial tRNA translation optimization 1
MTU1:	mitochondrial tRNA-specific 2-thiouridylase 1 or TRMU
NAD^+^:	nicotinamide adenine dinucleotide (oxidized form)
NADH:	nicotinamide adenine dinucleotide (reduced form)
NAP57:	nucleolar and coiled-body phosphoprotein 57 or dyskerin
NAT10:	*N*-acetyltransferase 10
Ncs6:	cytoplasmic tRNA 2-thiolation protein 1
Nep1/Emg1:	*N*^1^-specific pseudouridine methyltransferase
NFS1:	mitochondrial cysteine desulfurase
NifZ:	nitrogen fixation protein
Nm:	2′-*O*-methyl mark
NML:	nucleomethylin
NOP56:	nucleolar protein 56
NOP58:	nucleolar protein 58
NRF2:	nuclear factor erythroid 2 like 2
NSUN:	nucleolar protein 22/Sun domain RNA methyltransferase
Nt:	Nucleotide
Opa1:	optic atrophy 1
P body:	processing body
PAR:	poly(ADP-ribose)
PAR-CLIP:	photoactivatable ribonucleoside-enhanced crosslinking and immunoprecipitation
PCIF1:	phosphorylated C-terminal domain-interacting factor 1
PKR:	interferon-inducible double-stranded RNA-activated protein kinase or eIF-2A protein kinase 2
pri-miRNA:	primary microRNA transcripts
PRC2:	polycomb responsive complex 2
PRPP:	phosphoribosylpyrophosphate
PrrC:	anticodon nuclease
PRRC2A:	proline-rich coiled-coil 2A
PTC:	peptidyl transferase center
Pus:	pseudouridine synthase
Pus1p:	pseudouridine synthase 1 protein
Pus10:	pseudouridine synthase 10
QueA:	*S*-adenosyl-l-methionine-dependent tRNA ribosyltransferase-isomerase
QueCDE:	7-cyano-7-deazaguanine synthase, 6-carboxy-5,6,7,8-tetrahydropterine synthase, and 7-carboxy-7-deazaguanine synthase
QueF:	NADPH-dependent 7-cyano-7-deazaguanine reductase
QueF-like:	similar to NADPH-dependent 7-cyano-7-deazaguanine reductase
QueG:	epoxyqueuosine reductase
QueH:	epoxyqueuosine reductase
QueT:	queuosine precursor transporter
RaSEA:	radical SAM enzyme for archaeosine formation
Rit1:	ribosylation of initiator tRNA 1
RlhA:	23S rRNA 5-hydroxycytidine C2501 synthase
RlmA:	ribosomal RNA large subunit methyltransferase A or 23S rRNA (guanine(745)-*N*(1))-methyltransferase
RlmC:	ribosomal RNA large subunit methyltransferase C or 23S rRNA (uracil(747)-C(5))-methyltransferase
RlmD:	ribosomal RNA large subunit methyltransferase D or 23S rRNA (uracil(1939)-C(5))-methyltransferase
RlmF:	ribosomal RNA large subunit methyltransferase F
RlmFO:	ribosomal RNA large subunit methyltransferase FO or methylenetetrahydrofolate-tRNA-(uracil-5-)-methyltransferase
RlmG:	ribosomal RNA large subunit methyltransferase G
RlmH:	ribosomal RNA large subunit methyltransferase H
RlmJ:	ribosomal RNA large subunit methyltransferase J
RlmL:	ribosomal RNA large subunit methyltransferase L
RlmN:	23S rRNA m^2^A2503 methyltransferase/tRNA or dual-specificity RNA methyltransferase
RluA:	ribosomal large subunit pseudouridine synthase A or dual-specificity RNA pseudouridine synthase
RluD:	ribosomal large subunit pseudouridine synthase D
RNMT:	RNA guanine-7 methyltransferase
RRF:	ribosome release factor
RsmA:	ribosomal RNA small subunit methyltransferase A
RsmC:	ribosomal RNA small subunit methyltransferase C
RsmD:	ribosomal RNA small subunit methyltransferase D
RsmE:	ribosomal RNA small subunit methyltransferase E
RsmH:	ribosomal RNA small subunit methyltransferase H
RsmI:	ribosomal RNA small subunit methyltransferase I
RsuA:	ribosomal small subunit pseudouridine synthase A
SAH:	*S*-adensoyl-L-homocysteine
SAM:	*S*-adenosyl-L-methionine
SCARLET:	site-specific cleavage and radioactive labeling followed by ligation-assisted extraction and thin-layer chromatography
SCM-SAH:	*S*-carboxy-*S*-adenosyl-L-homocysteine
SELEX:	systematic evolution of ligands by exponential enrichment
SelU:	tRNA 2-selenouridine synthase
snoRNA:	small nucleolar RNA
snoRNP:	small nucleolar ribonucleo protein
SNP:	single nucleotide polymorphism
snR35:	small nucleolar RNA 35
snRNA:	small nuclear RNA
snRNP:	small nuclear ribonucleoprotein
SNU13:	small nuclear ribonucleoprotein 13 or 15.5K
SOS:	inducible DNA repair system
SRA:	steroid receptor RNA activator
T:	thymidine
Tad1p:	tRNA-specific adenosine deaminase 1 protein
Tad2p:	tRNA-specific adenosine deaminase 2 protein
Tad3p:	tRNA-specific adenosine deaminase 3 protein
TadA:	tRNA-specific adenosine deaminase
Tan1:	tRNA acetyltransferase 1
TapT:	tRNA aminocarboxypropyltransferase
Taw1–3:	SAM-dependent tRNA 4-demethylwyosine synthases 1–3
Taw22:	SAM-dependent tRNA 4-demethylwyosine synthase 22
TAZ:	tafazzin
TCD1/2:	threonylcarbamoyladenosine dehydratase 1 or 2
TcdA:	threonylcarbamoyladenosine dehydratase A
Tcs1/2:	threonylcarbamoyladenylate synthase 1 or 2
TermY:	tRNA m^1^Ψ54-methyltransferase
TET:	ten-eleven translocation methylcytosine dioxygenase
TET1:	ten-eleven translocation methylcytosine dioxygenase 1
Tgs1:	trimethylguanosine synthase 1
TGT:	queuine tRNA-ribosyltransferase
ThiI:	tRNA sulfurtransferase
TiaS:	tRNA^Ile^-agmatidine synthetase
TilS:	tRNA^Ile^-lysidine synthetase
Tit1:	tRNA isopentenyltransferase 1
tmRNA:	transfer-messenger RNA
TOR:	target of rapamycin
TP53:	tumor protein P53
TPA:	12-*O*-tetradecanoylphorbol-13-acetate
TPP:	thiamin pyrophosphate
TrhOP:	metallophos domain-containing protein O or P
TRIT1:	tRNA isopentenyltransferase 1
Trm-G10:	tRNA (guanine-10-*N*^2^)-dimethyltransferase
Trm1:	tRNA methyltransferase 1
Trm1p:	tRNA methyltransferase 1 protein
TRM4B:	tRNA cytosine-C^5^-methyltransferase
Trm5:	tRNA methyltransferase 5
Trm6-Trm61:	tRNA methyltransferases 6 and 61
Trm8p/Trm82p:	tRNA (guanine-*N*^7^-)-methyltransferase
Trm9/Trm112:	tRNA (carboxymethyluridine(34)-5-*O*)-methyltransferase protein
Trm10:	tRNA (guanine(9)-*N*^1^)-methyltransferase
Trm11p/112p:	tRNA (guanine(10)-*N*^2^)-methyltransferase
Trm140:	tRNA(Thr) (cytosine(32)-*N*^3^)-methyltransferase
TrmA:	tRNA/tmRNA (uracil-C^5^)-methyltransferase
TrmD:	tRNA (guanine-*N*^1^-)-methyltransferase
TrmFO:	methylenetetrahydrofolate-tRNA-(uracil-5-)-methyltransferase
TrmI:	tRNA m^1^A58/m^7^G46 methyltransferase
TrmM:	tRNA (adenosine(37)-*N*^6^)-methyltransferase or YfiC or TrmN6
TrmO:	tRNA methyltransferase O or tRNA (adenine(37)-*N*^6^-methyltransferase
TrmU:	tRNA 5-methylaminomethyl-2-thiouridylate methyltransferase
TRMT:	tRNA methyltransferase
TRMT1:	tRNA methyltransferase 1 or tRNA (guanine(26)-*N*(2))-dimethyltransferase
TRMT10:	tRNA methyltransferase 10
TRMT2A:	tRNA methyltransferase 2A or tRNA (uracil-5-)-methyltransferase
TRMT6:	tRNA methyltransferase 6 or tRNA (adenine(58)-*N*(1))-methyltransferase
TRMT12:	tRNA methyltransferase 12 homolog or tRNA wybutosine-synthesizing protein 2 homolog
TRMT61A:	tRNA methyltransferase 61A or tRNA (adenine(58)-*N*(1))-methyltransferase
TRMT61B:	tRNA methyltransferase 61B or tRNA (adenine(58)-*N*(1))-methyltransferase
TRMT112:	tRNA methyltransferase 112
TruA:	tRNA pseudouridine synthase A or Pus1
TruB:	tRNA pseudouridine synthase B or Pus4
TruD:	tRNA pseudouridine synthase D or Pus7
TtcA:	tRNA-cytidine-32 2-sulfurtransferase
TsaBCDE:	tRNA adenosine(37) threonylcarbamoyltransferase complex, dimerization subunit type 1; threonylcarbamoyladenylate synthase; tRNA adenosine(37) threonylcarbamoyltransferase complex, transferase subunit; tRNA adenosine (37) threonylcarbamoyltransferase complex, ATPase subunit type 1
TsaC2:	threonylcarbamoyladenylate synthase
Tsr3:	acp transferase ribosome maturation factor
TuaA:	tRNA U47 acp transferase A or YfiP
Tum1:	thiosulfate sulfurtransferase
Tus:	sulfur carrier protein or Moa
TYW1–5:	SAM-dependent tRNA 4-demethylwyosine synthases 1–5
UTR:	untranslated region
WBSCR22:	Willams-Beuren syndrome chromosomal region 22 protein
WTAP:	Wilms Tumor 1-Associating Protein
X-DC:	X-linked dyskeratosis congenita
YbbB:	tRNA 2-selenouridine/geranyl-2-thiouridine synthase
YbeA:	m^3^Ψ synthase or RlmH
YhhQ:	queuosine precursor transporter
YrvO:	cysteine desulfurase
YTH:	YT521-B protein domain
YTHDF1–3:	YTH *N*^6^-methyladenosine RNA binding proteins 1–3
ZCCHC4:	zinc finger CCHC-type containing 4

## 3 | DISCOVERY OF MODIFIED RIBONUCLEOSIDES: AN HISTORICAL RETROSPECTIVE ON PSEUDOURIDINE, INOSINE, AND DIHYDROURIDINE

*Nucleoside modification (IUPAC abbreviation)*: pseudouridine (Ψ); inosine (I); dihydrouridine (D).

*Domain assignments*: A, B, E.

*RNA classes*: ♣, 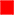, 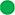 (Ψ and I), 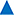 (Ψ and I).

tRNAs play a central role in translation. These adaptor molecules serve as links between the informational level of codons in mRNA and the functional level of amino acids incorporated into a growing polypeptide chain (Lorenz et al., 2017). From the initial total sequence determination of tRNA^Ala^, the specific locations of seven different modified nucleosides, including Ψ, inosine, and D, were described by Holley (1965). Since then, tRNAs have been recognized as the RNA class containing the highest percentage of post-transcriptional modifications (Figure 4, Table 2), which are mostly found in the tRNA loops (D- and TΨC-loops) and the tRNA anticodon loop. Currently, 111 distinct modifications have been identified in tRNAs from all three domains of life (Figure 3, Table 2). One such modification, and the first RNA modification to be discovered, is Ψ.

In 1951, Ψ was discovered as the first post-transcriptional modification in RNA and today is recognized as the most abundant RNA modification (Cohn & Volkin, 1951; reviewed in Spenkuch, Motorin, & Helm, 2014). Ψ is an isomer of uridine, where the nitrogen–carbon glycosidic bond is replaced by a carbon–carbon bond and an imine group is projected into the major groove (Figure 2). Ψ and U have identical molecular weights, making discerning Ψ and U problematic using size-based methods (L. Johnson & Söll, 1970). However, the use of CMCT and reverse transcription or the use of SCARLET are two methods frequently used to find Ψ in RNAs (Adachi, DeZoysa, & Yu, 2019; X. Li et al., 2015; N. Liu et al., 2013; Penzo, Guerrieri, Zacchini, Treré, & Montanaro, 2017). Ψ-seq uses CMCT to modify Ψ into *N*^3^-CMC-Ψ, which can terminate reverse transcription when preparing RNAs for RNA-seq (Carlile et al., 2014; Schwartz et al., 2014; B. S. Zhao & He, 2015). Because *N*^3^-CMC-Ψ blocks reverse transcription, the Ψ nucleotide is identified as one nucleotide downstream of the 3′ end of the fragment (Ofengand, Del Campo, & Kaya, 2001).

Pseudouridine synthases isomerize the U incorporated within RNA transcripts rather than individual ribonucleosides (L. Johnson & Söll, 1970). The synthases known to date are divided into five families (RluA, RsuA, TruA, TruB, and TruD) in bacteria. In archaeal and eukaryotic organisms, Pus family enzymes write pseudouridine marks (Hamma & Ferré-D’Amaré, 2006). The Pus enzymes have a conserved enzymatic core and use various protein domains to recognize different RNA targets (Hamma & Ferré-D’Amaré, 2006). However, the pseudouridine synthase NAP57, dyskerin, and other homologs of pseudouridine synthase, coupled with other core proteins, use H/ACA snoRNAs as guides to position a uridine at the active site of the machinery which introduces Ψ marks in rRNA and snRNAs (Ganot, Bortolin, & Kiss, 1997; reviewed in Y. T. Yu & Meier, 2014). Ψ is found nearly ubiquitously throughout various classes of RNAs (lncRNAs, mRNAs, miRNAs, mitochondrial tRNAs, rRNAs, small Cajal-body specific RNAs, snRNA, snoRNAs, and tRNAs) and in all three domains of life (reviewed in Penzo et al., 2017). However, it is unknown if any enzymes exist that revert Ψ back to U or completely remove Ψ from RNAs (reviewed in Eyler et al., 2019).

Nearly all tRNAs are populated with some abundance of Ψ, although some exceptions exist, such as the initiator tRNA in *Saccharomyces cerevisiae* (Boyer et al., 1970). Ψ55 is found in nearly all tRNAs within the TΨC stem-loop while other sites, such as the anticodon stem-loop and D-arm, also possess Ψ (Charette & Gray, 2000) (Figure 4, Table 2). U55 is modified to Ψ55 by tRNA Ψ55 synthase, an enzyme from the TruB family (Nurse, Wrzesinski, Bakin, Lane, & Ofengand, 1995). Ψ55 is proposed to stabilize the tertiary structure of tRNA, particularly under extreme thermal stress (Kinghorn, O’Byrne, Booth, & Stansfield, 2002). Within rRNA, Ψ can be found in both the small and large subunits of the ribosome in prokaryotes and eukaryotes as well as in chloroplasts and mitochondria (Charette & Gray, 2000). Ψ can be found in locations crucial for peptide bond formation, such as the PTC region (Ofengand, 2002). The inclusion of Ψ in various regions of tRNA and rRNA provides structural stabilization to enable the formation of highly ordered structures. snRNAs have Ψ within both the major (U1, U2, U4, U5, and U6) and minor (U12, U4atac, and U6atac) spliceosomal complexes (A. T. Yu, Ge, & Yu, 2011). Ψ in spliceosomal RNAs plays a crucial role in the assembly and function of snRNPs or the spliceosome via intermolecular RNA–RNA and RNA–protein interactions (A. T. Yu et al., 2011).

Ψ can be found in mRNA (5′ UTRs, coding sequences, and 3′ UTRs) (Carlile et al., 2014). Additionally, pseudouridylation of mRNA was found to have a role in translation. Ψ-containing mRNA impede *Escherichia coli* translation elongation by preventing the canonical interactions between the CCA-end of a tRNA and the A site of the PTC in ribosome (Eyler et al., 2019). Further, Ψ promoted aberrant amino acid substitutions, yielding multiple peptide products from one luciferase transcript in *E. coli* and human cells (Eyler et al., 2019). While many of these substitutions were anticipated not to have deleterious effects on luciferase, the authors also hypothesized that these substitutions could be harmful to host organisms under stress conditions (Eyler et al., 2019). As pseudouridylation helps stabilize RNA structures, Ψ is crucial in forming the active structure of the SRA lncRNA, which promotes transcription of several genes (X. Zhao et al., 2007; Y. Zhao, Dunker, Yu, & Karijolich, 2018). Moreover, mutating the pseudouridylation site to adenosine in mice causes SRA to become hyperpseudouridylated and act as a dominant negative suppressor of gene transcription, notably *c-myc* (X. Zhao et al., 2007; Y. Zhao, Dunker, et al., 2018).

Ψ has relevance in human disease, but its roles are not clearly defined in most diseases. Urine samples contain Ψ and could be used as a potential biomarker for cancer due to elevated levels in some cancer patients (Penzo et al., 2017; Seidel, Brunner, Seidel, Fritz, & Herbarth, 2006). Correlations between defects in Ψ formation and X-DC and Hoyeraal–Hreidarsson syndrome have been characterized (Penzo et al., 2017). Both diseases involve a mutation in the *dkc1* gene, leading to a decrease of Ψ in mRNA and many epidermal complications such as unusual skin pigmentation and white patches lining the oral cavity (leukoplakia) (Penzo et al., 2017). However, some individuals with either disease have been found to have normal levels of Ψ in mRNA, while telomerase impairment and shortened telomeres result in the diseases (Penzo et al., 2017). Separately, abrogation of Pus1p-mediated pseudouridylation of SRA has also been linked to MLASA in humans (Bykhovskaya, Casas, Mengesha, Inbal, & Fischel-Ghodsian, 2004; X. Zhao et al., 2007). Further studies are required to determine if pseudouridylation has a direct role in these diseases.

As early as 1957, inosine was discovered as a biosynthetic precursor to purine synthesis in every domain of life (Brown, Hoopes, White, & Sarisky, 2011; Graupner, Xu, & White, 2002; Hartman & Buchanan, 1958; Levin & Magasanik, 1961; Marolewski, Smith, & Benkovic, 1994; E. Meyer, Leonard, Bhat, Stubbe, & Smith, 1992; Nygaard & Saxild, 2000; Ownby, Xu, & White, 2005; L Warren, Flaks, & Buchanan, 1957). Inosine, which occurs in RNA and DNA, is the product of hydrolytic deamination at the C6 position of adenosine (Alseth, Dalhus, & Bjørås, 2014). In DNA, inosine formation can be caused by nonenzymatic processes, such as spontaneous hydrolysis, endogenous or environmental factors, or exposure to nitrosative compounds (Alseth et al., 2014). While these nonenzymatic processes can also occur in RNA, deaminase enzymes can also introduce inosine into RNAs (Alseth et al., 2014). In DNA, inosine preferentially base pairs with cytidine, where A-to-I deamination results in miscoding and is considered a premutagenic event (Alseth et al., 2014; Nordmann, Makris, & Reznikoff, 1988). In RNA, inosine can base pair to A, C, or U, allowing inosine to function as a Wobble base in the anticodon loop of tRNA or within other RNAs, such as mRNAs (reviewed in Wright, Force, & Znosko, 2018). Inosine was found in the Wobble position (I34) of tRNA^Ala^ (Holley, 1965) and in several tRNAs (Figure 4) (reviewed in Rubio et al., 2017; Torres et al., 2014). I34 was later determined to be essential in bacteria and eukaryotes, but not in archaea (reviewed in Rubio et al., 2017; Torres et al., 2014).

In contrast to DNA, enzymatically derived inosine modifications found in double-stranded RNA (dsRNA) occur via ADARs (Bass et al., 1997; Bass & Weintraub, 1988; Rebagliati & Melton, 1987) or ADATs (Bass et al., 1997; Danan-Gotthold & Levanon, 2017). Deamination of A-to-I has been reported in tRNAs (reviewed in Torres et al., 2014), recently for the first time in the small subunit of mitochondrial rRNA from the eukaryote *Diplonema papillatum* (Moreira, Valach, Aoulad-Aissa, Otto, & Burger, 2016), mRNAs (Paul & Bass, 1998), ncRNAs (Kawahara et al., 2007; Luciano, Mirsky, Vendetti, & Maas, 2004; Y. Yang, Zhou, & Jin, 2013), and viral RNAs (Samuel, 2011). Translational and splicing machinery recognize inosine as G, resulting in the altered informational content of the RNA. When A-to-I editing occurs in the coding region of an mRNA, the corresponding codon is altered, which can change the sequence of an encoded protein. Though recoding is rare, A-to-I editing events predominantly occur in the noncoding intronic or untranslated terminal regions of mRNAs and in ncRNAs (Athanasiadis, Rich, & Maas, 2004; D. D. Y. Kim et al., 2004; E. Y. Levanon et al., 2005; Y. Yang et al., 2013). For example, A-to-I editing events have been observed at repeated Alurich regions in RNAs from multiple classes (Levanon et al., 2004). Additionally, with several A-to-I editing events in the primary transcript of mouse miRNA-142 (pri-miR-142), both pri-miR-142 and the mature miR-142 levels are repressed (W. Yang et al., 2006). Consequently, in *adar1* or *adar2* null mice, both pri-miR-142 and mature miR-142 are increased (W. Yang et al., 2006). Interestingly, ADAR-catalyzed A-to-I conversion of DNA bases in DNA/RNA hybrids has been recently described, which may expand the spectrum of biological functions of ADARs (Y. Zheng, Lorenzo, & Beal, 2017).

While A-to-I editing is widespread across Metazoa, the number of edits can vary considerably across species (Porath, Knisbacher, Eisenberg, & Levanon, 2017). Three classes of ADAR proteins are involved in the regulation of pre-mRNA A-to-I editing in vertebrates. ADAR1 and ADAR2 are expressed in most tissues, while ADAR3 is expressed only in the central nervous system (C. X. Chen et al., 2000; Oakes, Anderson, Cohen-Gadol, & Hundley, 2017). Importantly, while ADAR1 and ADAR2 catalyze A-to-I editing (Zinshteyn & Nishikura, 2009), ADAR3 competitively interferes with the activity of RNA-bound ADAR1 and ADAR2, suggesting ADAR3 has a regulatory role (C. X. Chen et al., 2000). ADAT, a class of deaminases that are specific to tRNAs, are related to ADAR but are inactive on known pre-mRNA substrates and extended dsRNAs (Maas, Gerber, & Rich, 1999; Torres et al., 2014). ADAT1, or its homolog Tad1p, catalyzes the A-to-I conversion at position 37 in the anticodon loop of eukaryotic tRNA^Ala^ (Figure 4, Table 2) (A. Gerber, Grosjean, Melcher, & Keller, 1998), while the ADAT2/ADAT3 heterodimer or its homolog heterodimer Tad2p/Tad3p catalyzes the Wobble position A-to-I conversion of eukaryotic tRNAs (A. P. Gerber & Keller, 1999). The formation of I34 in bacterial tRNA^Arg^ is catalyzed by the TadA enzyme (A. A. H. Su & Randau, 2011; Wolf, Gerber, & Keller, 2002). Inosine at position 57 in the TΨC loop of tRNA has been reported for only archaea, where it occurs as *N*^1^-methylinosine (m^1^I57) (Figure 4) (Yamaizumi et al., 1982). Here, the first step of A-to-I conversion involves methylation of A57 by the SAM-dependent methyltransferase TrmI; this methylated adenosine is then deaminated to m^1^I57 by an unknown enzyme (Grosjean, Constantinesco, Foiret, & Benachenhou, 1995; Roovers et al., 2004; reviewed in Torres et al., 2014).

The effects of A-to-I editing have been observed primarily in brain tissue or neurons and can exhibit different phenotypes in these organs or cellular environments. A-to-I editing can affect brain tissue or neurons through dampening signaling through G-protein receptors, enhancing the activity of voltage-gated ion channels, promoting proper brain and neuron function, or proper splicing of glutamate-gated cation selective channels, among other observed functions (Burns et al., 1997; Hoopengardner, Bhalla, Staber, & Reenan, 2003; Levanon et al., 2005; Seeburg, Higuchi, & Sprengel, 1998; Sommer, Köhler, Sprengel, & Seeburg, 1991). ADAR activity plays an essential role in nervous system function by restoring or establishing neuronal growth (Benowitz, Goldberg, & Irwin, 2002). Further, mutations in ADARs have been shown to cause neural and behavioral defects in *Drosophila melanogaster* and *Caenorhabditis elegans* (Goldstein et al., 2017). Disrupted ADAR activity is associated with brain cancers, prostate cancer, hepatocellular carcinoma, and chronic myeloid leukemia (Fritzell, Xu, Lagergren, & Öhman, 2018; L.-D. Xu & Öhman, 2018). ADAR2 is essential for editing some mature miRNAs in glioblastoma cells and ADAR2 can also reduce the expression of numerous oncogenic miRNAs (Tomaselli et al., 2015). Astrocytoma and glioblastoma in humans have been correlated with an overexpression of ADAR3, in which ADAR3 likely competes with ADAR2 for a binding site in the *gria2* mRNA (Oakes et al., 2017). This competition prevents RNA editing and promotes cell migration as well as tumor invasion in glioblastoma (Oakes et al., 2017). A-to-I editing involvement in cancer, as well as neuronal behavior studies, remains an area of active research and could potentially lead to drug targets for cancer or behavioral treatments.

Another modification that was among the first found in total RNA was D in 1952 from beef spleen, though D had been synthesized previously (Funk, Merritt, & Ehrlich, 1952; Weidel & Roithner, 1896). D is a product of the post-transcriptional enzymatic reduction of the C5–C6 double bond in uridine to a single bond (Figures 1 and 2). Present in all three domains of life, D is a common modification in tRNA, where the D-loop was aptly named because it has several D modifications (Figure 4) (Sprinzl, Horn, Brown, Ioudovitch, & Steinberg, 1998). However, D also resides in the variable loop (V-loop) of tRNAs and in the *E. coli* 23S rRNA (Bishop, Xu, Johnson, Schimmel, & de Crécy-Lagard, 2002; Kowalak, Bruenger, & McCloskey, 1995; Xing, Hiley, Hughes, & Phizicky, 2004). D typically destabilizes stacking interactions with other nucleosides and D destabilizes the C3′-*endo* sugar conformation of its ribose, which is a hallmark for the formation of the A-RNA double helix (J. Dalluge, Hashizume, Sopchik, McCloskey, & Davis, 1996). Instead, D prefers a C2′-*endo* conformation, resulting in increased flexibility and dynamic motion where RNA tertiary interactions need to be accommodated (J. Dalluge et al., 1996). In psychrophiles, the increased usage of D is thought to be helpful in maintaining conformational flexibility of RNA under instances where thermal-based motion is limited or compromised (J. J. Dalluge et al., 1997). Finally, D is an important factor in ensuring the recognition of tRNA by specific aminoacyl-tRNA synthetases and the fidelity of translation (Hendrickson, 2001).

Though D is an abundant modification in all three domains of life, Dus enzymes that catalyze the formation of D belong to a broad family of FMN-dependent enzymes (Bishop et al., 2002). Three classes of Dus enzymes are found in *E. coli* (DusA, DusB, and DusC), four classes are found in *S. cerevisiae* (Dus1p, Dus2p, Dus3p, and Dus4p), and one class is archaeal (Dus) (Bishop et al., 2002; Kasprzak, Czerwoniec, & Bujnicki, 2012). Thus far, one homolog has been discovered in humans (hDus2p), though homologs for Dus1, Dus3, and Dus4 may also exist in humans (Kasprzak et al., 2012; Whelan et al., 2015). By conducting a structural analysis of bacterial DusABC, it was determined that DusB is the ancestral Dus enzyme (Bou-Nader et al., 2018). Additional structural analyses of DusABC proteins revealed that each Dus enzyme has distinct substrate specificity on tRNAs (Bou-Nader et al., 2018). Further analysis of the otherwise evolutionarily conserved double-stranded RNA-binding domain of hDus2p revealed a novel N-terminal extension in the hDus2p enzyme, which confers the ability to recognize specifically tRNAs, instead of nonspecifically recognizing double-stranded RNAs (Bou-Nader et al., 2019).

Because elevated levels of dihydrouridine were correlated with Novikoff hepatoma and in Ehrlich ascites previously (Kuchino & Borek, 1978), subsequent research sought to determine involvement of hDus2p in cancer (T. Kato et al., 2005). In lung cancer, there is an increased amount of hDus2p, leading to greater amounts of D, which in turn increases conformational flexibility of tRNAs and enhances translational efficiencies (T. Kato et al., 2005). Concordantly, overexpression of hDus2p was correlated with a poor prognosis in lung cancer patients (T. Kato et al., 2005). Separately, hDus2p interacts with PKR, inhibiting its kinase activity and interferon-induced antiviral innate immunity (Mittelstadt et al., 2007). Overexpression of hDus2p also inhibits stress-induced apoptosis in sarcoma cells (Mittelstadt et al., 2007). The discovery of additional Dus homologs in humans may provide further clues in its involvement in human health. Moreover, the natural occurrence of D in psychrophilic organisms may provide useful applications or insights into synthetic biological applications at colder temperatures. The discovery and characterization of the modifications Ψ, I, and D served as a basis for the subsequent discovery and characterization of modifications in RNA from all three domains of life.

## 4 | ADENOSINE-DERIVED MODIFICATIONS

### 4.1 | The roles of m^6^A in biology and its metabolic breakdown products of hm^6^A and f^6^A

*Nucleoside modification (IUPAC abbreviation)*: *N*^6^-methyladenosine (m^6^A); *N*^6^-hydroxymethyladenosine (hm^6^A); *N*^6^-formyladenosine (f^6^A).

*Domain assignments*: A (m^6^A), B (m^6^A), E.

*RNA classes*: ♣ (m^6^A), 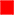 (m^6^A), 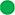, 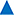 (m^6^A).

The biological function of *N*^6^-methyladenosine (m^6^A) represents one of the most prolific areas of recent research in modified ribonucleosides. Although m^6^A was originally discovered in 1974 in mRNA (Perry & Kelley, 1974), it was 25 years later when the first enzyme that could synthesize m^6^A (known as an m^6^A writer) was discovered. For eukaryotic mRNAs, this writer was identified as METTL3, which exists in a complex with the catalytically inactive METTL14 and WTAP (Bokar, Shambaugh, Polayes, Matera, & Rottman, 1997; Ping et al., 2014; Shi et al., 2019). METTL3/14 methylate RNAs at a consensus nucleotide sequence: 5′-RRACH-3′ (R = purine, H = A, C, or U nucleotides) (Dominissini et al., 2012; K. D. Meyer et al., 2012). Subsequent m^6^A methyltransferases were discovered in humans and *E. coli* acting on different RNA classes. One of these methyltransferases is human METTL16, which modifies the U6 snRNA, the MAT2A mRNA, and likely other RNAs (Koh, Goh, & Goh, 2019; Pendleton et al., 2017; Warda et al., 2017). Recently, the heterodimer complex METTL5 and Trmt12 was characterized as an m^6^A methyltransferase that modifies A1832 in the human 18S rRNA (van Tran et al., 2019) and ZCCHC4 was recently characterized as an m^6^A methyltransferase that modifies A4220 in the human 28S rRNA (Pinto et al., 2020; van Tran et al., 2019). RlmF and RlmJ place two distinct m^6^A marks (A1618 and A2030, respectively) on the *E. coli* 23S rRNA (Golovina et al., 2012; Sergiev, Serebryakova, Bogdanov, & Dontsova, 2008). Although m^6^A is present in bacterial mRNAs, the methyltransferase(s) that confer m^6^A marks remains elusive. The consensus sites for *E. coli* and *Pseudomonas aeruginosa*, respectively, are 5′-UGCCAG-3′ or 5′-GGYCAG-3′, which are distinct from the 5′-RRACH-3′ sequence recognized by METTL3/14 in humans (Deng et al., 2015). Moreover, m^6^A modifications have been detected in one tRNA^Val^ species in *E. coli* (Saneyoshi, Harada, & Nishimura, 1969) and its writer was determined to be TrmM (Golovina et al., 2009). Further, an m^6^A modification at A1500 has been detected in the small ribosomal subunit of the crenarchaeote *Sulfolobus solfataricus* and *Haloferax volcanii* (Kowalak, Bruenger, Crain, & McCloskey, 2000; Noon, Bruenger, & McCloskey, 1998), although it is currently unknown which enzymes confer these modifications.

For some RNAs from bacterial and eukaryotic organisms, there are validated demethylases that can remove m^6^A marks from RNAs (known as m^6^A erasers) (Fedeles, Singh, Delaney, Li, & Essigmann, 2015). One such demethylase is the ALKBH5 protein, which specifically removes m^6^A marks from mRNAs (G. Zheng et al., 2013). Another demethylase is the FTO protein, which demethylates m^6^A, m^6^Am, and m^1^A marks in mRNA, snRNA, and tRNA, respectively (Shi et al., 2019; Wei et al., 2018; X. Zhang, Wei, et al., 2019). Instead of a direct demethylation reaction, human and mouse FTO can demethylate m^6^A marks by oxidizing them to yield hm^6^A, which is further oxidized to yield f^6^A (Fu et al., 2013). The half-lives of these modifications, whose biological roles remain unexplored, is ~3 hr in vitro, and then both modifications nonenzymatically decompose back to adenosine (Fu et al., 2013). The reversibility of m^6^A marks in mRNAs remains controversial, for a small percent of m^6^A marks vary during the life of an mRNA (Darnell, Ke, & Darnell, 2018; Zaccara, Ries, & Jaffrey, 2019; B. S. Zhao, Nachtergaele, Roundtree, & He, 2018). Further, the role of m^6^A in splicing has been questioned because mouse embryonic stem cells without *Mettl3* continue to grow and properly splice mRNAs (Darnell et al., 2018). However, there is agreement that nascent mRNAs are cotranscriptionally modified with m^6^A marks and that m^6^A-containing mRNAs are more expeditiously turned over than non-m^6^A mRNAs (Darnell et al., 2018; B. S. Zhao, Nachtergaele, et al., 2018). Regardless, the removal of m^6^A is a controlled process, though it is unclear what significance hm^6^A and f^6^A have in biology.

In addition to m^6^A writers and erasers, several proteins have been characterized whose binding activity depends on the presence or absence of an m^6^A mark (known as m^6^A readers). For example, m^6^A marks may weaken secondary structure to create new protein-binding sites, such as hnRNPC binding to a U-rich loop in an m^6^A-disrupted hairpin (Alarcón, Goodarzi, et al., 2015; N. Liu et al., 2015, 2017; Shi et al., 2019; B. Wu et al., 2018). These findings led to the model of an m^6^A switch, whereby the absence of an m^6^A mark may result in a stable hairpin that obscures protein binding sequences (N. Liu et al., 2015, 2017; X. Wang et al., 2014). Another possible m^6^A switch regulates miRNA-binding site accessibility in MALAT1. Here, the absence of an m^6^A mark in MALAT1 may prevent the formation of a long-range pseudoknot and, concordantly, expose two miRNA-binding sites that could sequester miRNAs away from their oncogenic mRNA targets (McCown, Wang, Jaeger, & Brown, 2019). Proteins containing the YTH domain, such as YTHDF2, can recognize and bind to m^6^A marks on mRNAs and ncRNAs in humans. These binding events destabilize the structure of their RNA targets and then lead these YTHDF2-bound RNAs to P bodies for degradation (H. Du et al., 2016; Shi et al., 2017, 2019; X. Wang et al., 2014, 2015). IGF2BP1–3 proteins increase the mRNA half-life of mRNAs with m^6^A marks, while PRRC2A also increases mRNA half-life of an m^6^A-containing transcript that is necessary for myelination (H. Huang et al., 2018; Shi et al., 2019; R. Wu et al., 2019). The binding of the splicing-factor hnRNPA2B1 enables m^6^A-dependent nuclear processing, especially for pri-miRNAs (Alarcón, Goodarzi, et al., 2015). METTL3 and its corresponding m^6^A marks are sufficient to permit proper pri-miRNA processing by DROSHA in a non-cell-type specific fashion in human cells (Alarcón, Lee, Goodarzi, Halberg, Tavazoie, 2015). hm^6^A and f^6^A are not recognized by the YTHDF2 protein, suggesting that they could either bind to different RNA-binding proteins or serve as markers for newly synthesized RNAs, among other possibilities (Fu et al., 2013). Recently, it was discovered that YTHDF1–3 facilitates phase separation of m^6^A-marked mRNAs and influences the transport of these mRNAs into different phase-separated compartments: P bodies for nonstress conditions, or stress granules for heat shock or arsenite stress (Ries et al., 2019).

The involvement of m^6^A in human health is a highly active area of research that cannot be adequately addressed in this review. Several recent reviews capture the trends on m^6^A in disease (X.-Y. Chen, Zhang, & Zhu, 2019; K. Du, Zhang, Lee, & Sun, 2019; Lan et al., 2019; Tong, Flavell, & Li, 2018). Nonetheless, we highlight some of the more striking discoveries involving m^6^A in human disease. In select cancer cells, an abundance of METTL3 was found to promote A549 lung adenocarcinoma cancer cell growth, survival, and invasion phenotypes, while the methylation activity of METTL3 was found to regulate the protein expression of several oncogenes, including EGFR, TAZ, MK2, and DNMT3A (S. Lin, Choe, Du, Triboulet, & Gregory, 2016). METTL3 enhances the translation of these oncogenes and others by physically interacting with eIF3H, which increases the formation of polysomes on the EGFR, TAZ, MK2, and DNMT3A mRNAs (Choe et al., 2018). Small molecule ligands that bind to the METTL3–METTL14–WTAP complex activate this complex and may aid in cell survival after UV exposure (Selberg et al., 2019; Xiang et al., 2017). YTHDF2 is often overexpressed in AML and is required for AML initiation and propagation (J. Paris et al., 2019). YTHDF2 was found to be dispensable in noncancerous hematopoietic stem cell function, yet essential for leukemic stem cell function. Concordantly, YTHDF2 represents an attractive therapeutic target for AML, which also permits hematopoietic stem cell expansion after treatment (J. Paris et al., 2019). Small molecule inhibitors targeting FTO have eradicated AML cells in vitro and in mouse xenograft models of AML (Y. Huang et al., 2019). In neurology, m^6^A marks were shown to shorten the radial glial cell cycle, while also extending cortical neurogenesis beyond birth in mouse brains (Yoon et al., 2017). Moreover, m^6^A marks are significant in memory formation in mice, as a knockout of the *ythdf1* gene in mice showed learning and memory deficits (Shi et al., 2018). In cardiology, m^6^A marks have been shown to be crucial in proper heart development and in heart function; for an siRNA knockdown of METTL3 and a cardiomyocyte-specific knockout mouse line of *mettl3* resulted in the depletion of m^6^A, which also resulted in increased rates of heart failure in mice and possibly in humans (Dorn et al., 2019). Much research has been conducted on the significance of the presence or absence of m^6^A marks to human health. However, further research is needed to determine precisely how m^6^A, hm^6^A, and f^6^A affect human health instead of correlative observations based on their global alterations in knocking down or overexpressing enzymes that write or erase these modifications. Also, research into the significance of hm^6^A and f^6^A remains almost nonexistent and may further elucidate the temporal presence of m^6^A in RNAs.

### 4.2 | The methyl groups of m^1^A and its derivative m^1^I disrupt hydrogen bonding along the Watson–Crick face of their host ribonucleotides

*Nucleoside modification (IUPAC abbreviation)*: *N*^1^-methyladenosine (m^1^A); *N*^1^-inosine (m^1^I).

*Domain assignments*: A, B, E.

*RNA classes*: ♣, 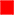 (m^1^A), 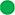 (m^1^A), 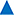 (m^1^A).

m^1^A is a prevalent, dynamic base modification found in tRNAs, rRNAs, mRNAs, and ncRNAs (Bar-Yaacov et al., 2016, 2017; Dominissini et al., 2016; Khoddami et al., 2019; Kirpekar, Hansen, Rasmussen, Poehlsgaard, & Vester, 2005; Koscinski, Feder, & Bujnicki, 2007; X. Li et al., 2016; Maden, 1990; McCown et al., 2019; Oerum, Dégut, Barraud, & Tisné, 2017; Roovers et al., 2004; Safra et al., 2017; Schwartz, 2018; Sharma, Watzinger, Kötter, & Entian, 2013; Wachino et al., 2007). m^1^A disrupts U-A Watson–Crick base pairing and introduces a positive charge at physiological pH (Dominissini et al., 2016; Parsyan et al., 2011; Roundtree, Evans, Pan, & He, 2017; Zur & Tuller, 2013). m^1^A marks have been found at several positions in tRNAs in all three domains of life (Figure 4, Table 2) (Oerum et al., 2017; Roovers et al., 2008; Sprinzl et al., 1998). The two most well-studied m^1^A modifications in tRNA are m^1^A9 and m^1^A58, which have been related to proper tRNA folding and structural stability (reviewed in Oerum et al., 2017). The m^1^A9 modification has been found in archaeal and mitochondrial mammalian tRNAs (reviewed in Oerum et al., 2017). In human mitochondrial tRNA^Lys^, tRNA^Leu(UUR)^, and tRNA^Asp^, the m^1^A9 modifications disrupt Watson–Crick interactions that would otherwise render these tRNAs as hairpins. Instead, these tRNAs possess the functional L-shape structure seen in other tRNAs (Figure 4, Table 2) (Helm et al., 1998; Helm, Giegé, & Florentz, 1999; Helm & Attardi, 2004; Oerum et al., 2017; Voigts-Hoffmann et al., 2007). Recently, the enzyme hTRMT10B was indicated as the tRNA^Asp^-specific m^1^A9 methyltransferase in humans (Howell, Jora, Jepson, Limbach, & Jackman, 2019). The m^1^A58 modification in tRNAs can be found in all three domains of life, though it is found in both cytosolic and mitochondrial tRNAs of eukaryotes (reviewed in Oerum et al., 2017). For the tRNA-specific m^1^A58 modification, prokaryotes use the enzyme TrmI to methylate A58, while the enzyme complex Trm6–Trm61 (previously known as Gcd10p/Gcd14p) methylates this adenosine in *S. cerevisiae* (Droogmans et al., 2003; Oerum et al., 2017; Roovers et al., 2004). Among prokaryotic organisms such as *Thermus thermophilus*, m^1^A58, Gm18, and m^5^s^2^U54 have been linked to the structural thermostability of tRNA as these modifications increase the melting temperature of tRNA by 10°C when compared with unmodified tRNAs (Droogmans et al., 2003; Oerum et al., 2017; Yokoyama, Watanabe, & Miyazawa, 1987). In *S. cerevisiae*, m^1^A58 has been shown to be important for the maturation of the initiator tRNA^Met^ (Anderson et al., 1998). In humans, m^1^A58 situated in tRNA^Lys3^ appears to be essential for the fidelity and efficiency of plus-strand DNA transfer during HIV-1 reverse transcription (Auxilien, Keith, Le Grice, & Darlix, 1999). m^1^A marks have also been discovered in rRNA in archaeal and aminoglycoside-resistant bacteria. The m^1^A1408 modification in 16S rRNA disrupts the binding of aminoglycosides to rRNA in *E. coli* (Kirpekar et al., 2005; Koscinski et al., 2007; Wachino et al., 2007). Like prokaryotes, m^1^A modifications can be found in eukaryotic rRNA, such as A645 in *S. cerevisiae* 25S rRNA and A1322 in human 28S rRNA. This modification functions in the biogenesis of the human 60S ribosome through methylation of A1322, as catalyzed by NML (Peifer et al., 2013; Sharma et al., 2018; Waku et al., 2016). In the mitochondrial 16S rRNA, the enzyme TRMT61B confers this methyl mark (Bar-Yaacov et al., 2016, 2017).

For m^1^A modifications in human mRNAs, TRMT6/TRMT61A has been shown to confer this mark (Schwartz, 2018). For m^1^A erasers, FTO and ALKBH3 have been shown to demethylate m^1^A marks in human tRNAs and mRNAs (Wei et al., 2018; Woo & Chambers, 2019). The presence of m^1^A in mRNAs increases the efficiency of translation initiation, early elongation, and enhances translation rates (Dominissini et al., 2016; Roundtree et al., 2017). m^1^A has been determined to be involved in cancer, as either drastic upregulation or downregulation of m^1^A levels and m^1^A writer proteins are found in patients with poor prognoses in several cancer types, including gastrointestinal cancers, hepatocellular carcinoma, pancreatic adenocarcinoma, and colorectal adenocarcinoma (Y. Zhao et al., 2019).

In archaeal tRNAs, m^1^A marks are present at positions 57 and 58, where m^1^A57 is an obligatory precursor in the formation of m^1^I (Droogmans et al., 2003; Grosjean et al., 1995; Roovers et al., 2004; Yamaizumi et al., 1982). Adenosine methylation occurs first via the TrmI enzyme to form the m^1^A marks; m^1^A57 is further deaminated by an unknown enzyme to form m^1^I (Grosjean et al., 1995, 1996; Roovers et al., 2004). m^1^I has also been found in eukaryotic cytosolic tRNA^Ala^ at position m^1^I37 (Grosjean et al., 1995, 1996; Holley, 1965). The synthesis of m^1^I in eukaryotes differs from archaea, however. In *S. cerevisiae* and animal cells, the homodimeric Tad1p and ADAT1 enzymes, respectively, catalyze the conversion of A37 to I37 (A. Gerber et al., 1998; Maas et al., 1999). I37 is then methylated to m^1^I by Trm5 in a SAM-dependent reaction (Björk et al., 2001). In the autoimmune disease polymyositis, antibodies were detected and could target m^1^I-containing tRNAs and stem-loops (Grosjean et al., 1996). Though m^1^A-related health research is being conducted, further m^1^I-related research may also yield interesting discoveries in human health that are currently unknown.

### 4.3 | i^6^A and the related ribonucleosides io^6^A, ms^2^i^6^A, ms^2^io^6^A, and msms^2^i^6^A have roles in tRNA formation, translation, cytokinin formation, and prion development

*Nucleoside modification (IUPAC abbreviation)*: *N*^6^-isopentenyladenosine (i^6^A); *N*^6^-(*cis*-hydroxyisopentenyl)-adenosine (io^6^A); 2-methylthio-*N*^6^-isopentenyladenosine (ms^2^i^6^A); 2-methylthio-*N*^6^-(*cis*-hydroxyisopentenyl)-adenosine (ms^2^io^6^A); 2-methylthiomethylenethio-*N*^6^-isopentenyl-adenosine (msms^2^i^6^A).

*Domain assignments*: B, E (not msms^2^i^6^A).

**FIGURE 5:**
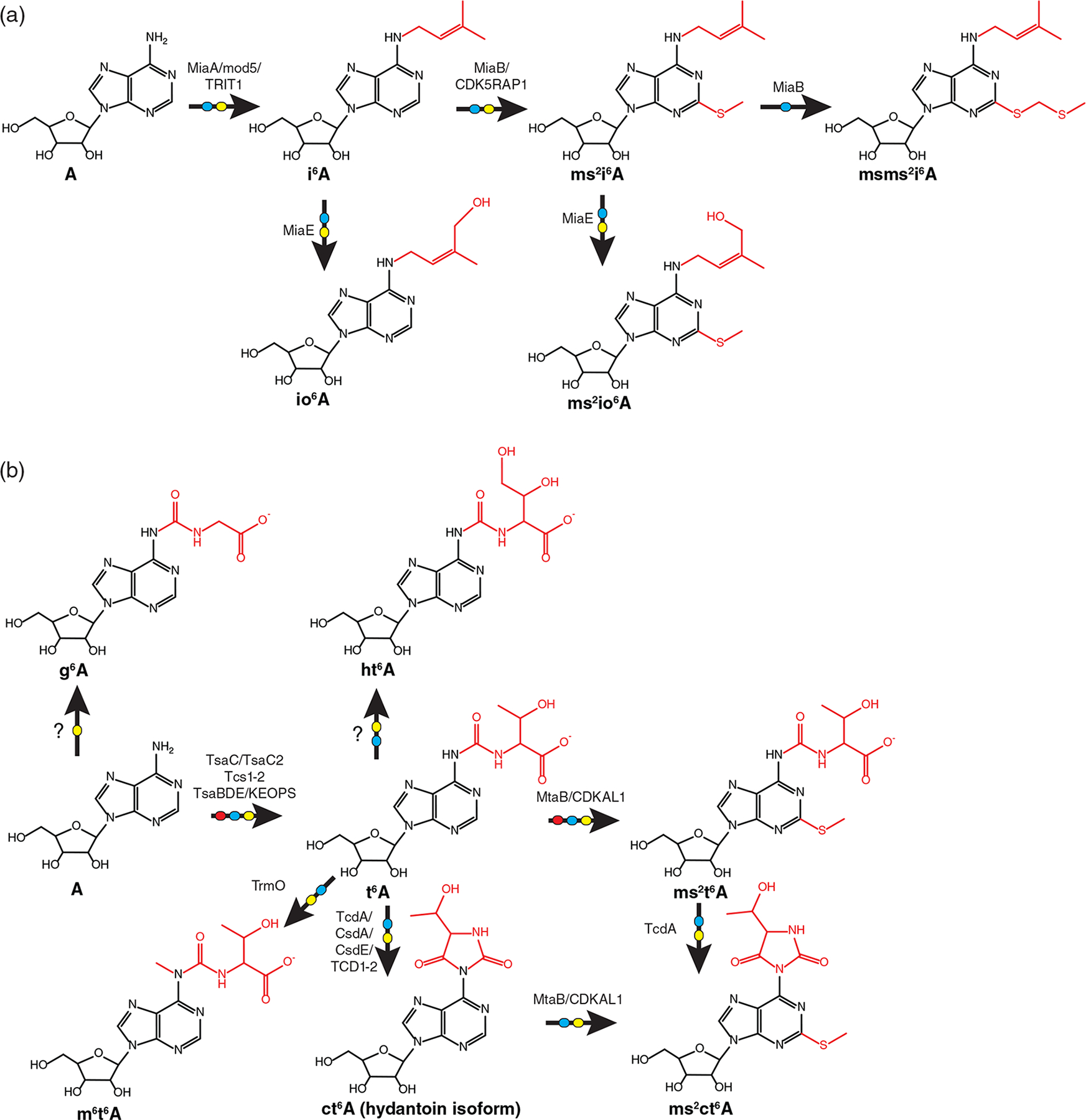
Schemes showing the production of i^6^A and t^6^A derivatives of adenosine. (a) Modifications that occur in the production of i^6^A, io^6^A, ms^2^i^6^A, ms^2^io^6^A, and msms^2^i^6^A. (b) Modifications that occur in the production of g^6^A, t^6^A, ht^6^A, ms^2^t^6^A, m^6^t^6^A, ct^6^A, and ms^2^ct^6^A. In both panels, arrows denote enzymatic reactions and enzyme(s) that participate in each reaction are abbreviated if known (see abbreviations list) or denoted by a question mark (?) if unknown. Red moieties represent the modification(s) added to the adenosine molecule. Arrow tails containing red, blue, or yellow ellipses denote reactions that occur in archaea, bacteria, or eukaryotes, respectively

*RNA classes*: ♣.

i^6^A appears at position 37 in many tRNA molecules, corresponding to the anticodon loop (nt 37 in Figure 4) (U. Schweizer, Bohleber, & Fradejas-Villar, 2017). In bacteria and eukaryotes, the synthesis of i^6^A within a tRNA is accomplished by conjugating an isopentenyl pyrophosphate (IPP) molecule to A37 via the bacterial miaA enzyme, the *S. cerevisiae* mod5 enzyme, the human TRIT1 enzyme, or organism-relevant homologs (Figure 5a) (U. Schweizer et al., 2017). In select plants, i^6^A can also exist as an unincorporated ribonucleoside excised from a recycled tRNA molecule or synthesized as a ribonucleotide for use as a hormone (Hwang & Sakakibara, 2006; Riefler, Novak, Strnad, & Schmülling, 2006). i^6^A can be hydroxylated by the bacterial MiaE enzyme and its eukaryotic homologs to yield io^6^A (U. Schweizer et al., 2017).

Further modifications of i^6^A are established by bacterial MiaB and mammalian CDK5RAP1 enzymes, which perform a thiomethylation of i^6^A to form ms^2^i^6^A (U. Schweizer et al., 2017). ms^2^i^6^A can be further modified by the miaE enzyme, which hydroxylates the ms^2^i^6^A modification to form ms^2^io^6^A. While ms^2^io^6^A is chemically distinct from ms^2^i^6^A, the two appear to be functionally interchangeable in stabilizing weak base pairing between the mRNA codon and the tRNA anticodon (Persson, Esberg, Ólafsson, & Björk, 1994). The sulfur in ms^2^i^6^A and ms^2^io^6^A comes from cysteine and is delivered by the cysteine desulfurase IscS, itself a hub of seven different pathways in which sulfur can be utilized in the biogenesis of ms^2^i^6^A/ms^2^io^6^A, s^2^U, s^4^U, s^2^C, thiamin, Moco, and additional Fe-S proteins (Shigi, 2014). msms^2^i^6^A, predominantly found in *E. coli* tRNA^Ser^ and tRNA^Tyr^, was determined to be synthesized from the MiaB enzyme in a sequential thioacetylation reaction using ms^2^i^6^A as a precursor in vitro (Dal Magro et al., 2018). However, it is unknown whether msms^2^i^6^A is found in other organisms and its function remains unknown.

At position 37 in the anticodon loop, the modified ribonucleosides i^6^A, ms^2^i^6^A, m^1^G, inosine, m^1^A, m^1^I, m^2^A, and yW and its derivatives disrupt potential base pairing with a neighboring uridine residue (U33) that is close to A37 in the tertiary structure of several tRNA species (U. Schweizer et al., 2017). These modifications also strengthen anticodon–codon pairings of adjacent tRNA nucleotides to their cognate mRNA targets (U. Schweizer et al., 2017). i^6^A and ms^2^i^6^A have significant roles in proper codon recognition in tRNA molecules, particularly in the stationary phase sigma factor in *E. coli* (U. Schweizer et al., 2017). Moreover, i^6^A formation on tRNA^Trp^ was shown to optimize *trp* operon attenuation in bacteria (Eisenberg, Yarus, & Soll, 1979). In bacteria, ms^2^i^6^A limits P-site slippage in the ribosome, while also increasing the synthesis of an iron chelator (Buck & Griffiths, 1982; reviewed in Pollo-Oliveira & De Crécy-Lagard, 2019; Urbonavicius, Qian, Durand, Hagervall, & Björk, 2001). On elucidating its role in iron chelation, it was discovered that MiaB is dependent upon an iron–sulfur cluster (Pierrel, Björk, Fontecave, & Atta, 2002). Further, the translation of the major iron homeostasis regulator in *E. coli*, Fur, is coupled to translation of a leader peptide that is properly decoded by a tRNA^Ser^-UGA which is modified at A37 to contain an ms^2^i^6^A mark (Pollo-Oliveira & De Crécy-Lagard, 2019; Veĉerek, Moll, & Bläsi, 2007). In *S. cerevisiae*, a reduction in the efficiency of the tRNA^Tyr(UAA)^ suppressor was attributed to lack of i^6^A (Laten, Gorman, & Bock, 1978; U. Schweizer et al., 2017). Further, *S. cerevisiae* strains that were homozygous for a deletion of *mod5* fail to sporulate (Laten et al., 1978). The mod5 protein in *S. cerevisiae* can also form prions, which were shown to regulate the sterol biosynthesis pathway and ultimately led to an acquired resistance against several azole-derived antifungal agents (G. Suzuki, Shimazu, & Tanaka, 2012). Prion formation of mod5 also resulted in a decrease of i^6^A production (G. Suzuki et al., 2012). In *Schizosaccharomyces pombe*, the *mod5* homolog *tit1* renders resistance to rapamycin, an inhibitor of mTOR (U. Schweizer et al., 2017).

Within other eukaryotes, mod5 or its homologs can modify cytosolic and mitochondrial tRNAs. Both *S. cerevisiae* and *S. pombe* retain a preference for using i^6^A in mitochondrial tRNAs. However, the preferential modification in mammals shifts to using the ms^2^i^6^A modification in mitochondrial tRNAs; ms^2^i^6^A may also be absent in cytosolic tRNAs. Finally, in mammals, selenoprotein expression is dependent upon proper formation of i^6^A and mcm^5^U in tRNA^[Ser]Sec^ (U. Schweizer et al., 2017; Sierant et al., 2018).

Within plants, i^6^A functions as a cytokinin, a plant hormone with regulatory roles in plant growth and proper plant development (Riefler et al., 2006). The i^6^A receptors CRE1/AHK4 and AHK3 are expressed significantly in root tissue and aerial portions of *Arabidopsis*, respectively (Riefler et al., 2006). CRE1/AHK4 has roles in phosphate starvation responses and sulfate acquisition, in addition to the formation of significant defects in the formation and development of plant vascular tissues (Riefler et al., 2006). Further, these receptors have roles in regulating germination rates, light needed for germination, and sensitivity to far-red light (Riefler et al., 2006). Within humans, the role of i^6^A as a cytokinin has been investigated as a potential antitumor treatment for the last 50 years (Dassano et al., 2014). Though the initial investigation into i^6^A in clinical trials failed, the development of i^6^A analogs has yielded compounds that inhibit growth of the MCF7 breast cancer cell line and activate the NRF2-mediated oxidative stress response, which is triggered typically by hydrogen peroxide or TPA (Dassano et al., 2014). What roles i^6^A or its derivatives may have as an anticancer drug remain unknown but are quite exciting. Further, while IPP is used to generate i^6^A, it remains unknown if any additional IPP derivatives are used in organisms to modify adenosine molecules. Specifically, as a geranyl moiety can be used in modifying uridine molecules (Sierant et al., 2016), it would be fascinating to see if geranyl pyrophosphate or another lipid molecule can similarly be incorporated into an adenosine molecule.

### 4.4 | The biological roles of t^6^A and its derivatives or relatives: ct^6^A, m^6^t^6^A, ms^2^t^6^A, ms^2^ct^6^A, ht^6^A, ac^6^A, g^6^A, hn^6^A, and ms^2^hn^6^A

*Nucleoside modification (IUPAC abbreviation)*: *N*^6^-threonylcarbamoyladenosine (t^6^A); cyclic *N*^6^-threonylcarbamoyladenosine (ct^6^A); *N*^6^-methyl-*N*^6^-threonylcarbamoyladenosine (m^6^t^6^A); 2-methylthio-*N*^6^-threonylcarbamoyladenosine (ms^2^t^6^A); cyclic 2-methylthio-*N*^6^-threonylcarbamoyladenosine (ms^2^ct^6^A); *N*^6^-hydroxythreonylcarbamoyladenosine (ht^6^A); *N*^6^-acetyladenosine (ac^6^A); *N*^6^-glycinylcarbamoyladenosine (g^6^A); *N*^6^-hydroxynorvalylcarbamoyladenosine (hn^6^A); 2-methylthio-*N*^6^-hydroxynorvalylcarbamoyladenosine (ms^2^hn^6^A).

*Domain assignments*: A (t^6^A, ac^6^A, hn^6^A, ms^2^t^6^A, and ms^2^hn^6^A), B (not g^6^A, ht^6^A), E (not ac^6^A, hn^6^A, and ms^2^hn^6^A).

*RNA classes*: ♣.

t^6^A and subsets of its derivatives (Figure 5b) are essential modified bases found in all three domains of life at position 37 of tRNAs (Figure 4, Table 2), comparable with i^6^A and its derivatives above (Kang et al., 2017). Several tRNAs that read codons starting with a U residue are universally modified to contain t^6^A or its derivatives, while various tRNAs that read codons that start with an A residue are modified to contain i^6^A or its derivatives (U. Schweizer et al., 2017). To create t^6^A, all three domains of life undergo a two-step process to covalently attach threonine to the N6 position of A37 in tRNA molecules. In bacteria, the TsaC or the TsaC2 protein adds a threonylcarbamoyl group onto an ATP molecule, whereas archaea and eukaryotes use Tcs1 or Tcs2 (reviewed in Thiaville, Iwata-Reuyl, & de Crécy-Lagard, 2014). Once the resulting threonylcarbamoyl-AMP molecule is formed, bacteria use the TsaBDE proteins to transfer the threonylcarbamoyl moiety into A37 to form t^6^A37, while archaea and eukaryotes use the KEOPS macromolecular complex to form t^6^A37 (reviewed in Thiaville et al., 2014; Wan et al., 2013). Instead of incorporation with threonine into A37 in tRNAs, a glycine residue can occasionally be placed on the tRNA instead, generating g^6^A through an unknown enzymatic process (M. P. Schweizer, McGrath, & Baczynskyj, 1970; Wan et al., 2013). t^6^A can be cyclized into ct^6^A by the *E. coli* enzymes TcdA, CsdA, and CsdE or the *S. cerevisiae* homologs TCD1-TCD2 (Miyauchi, Kimura, & Suzuki, 2013). t^6^A can also be further methylated or hydroxylated, forming m^6^t^6^A or ht^6^A via the enzyme TrmO and by an unknown enzyme, respectively (Figure 5b) (Kang et al., 2017; Nagao et al., 2017). In all three domains of life, a thiomethyl group from SAM can also be added onto t^6^A by the bacterial MtaB enzyme or the eukaryotic enzyme CDKAL1, forming ms^2^t^6^A (Arragain et al., 2010). ms^2^t^6^A can be further cyclized into ms^2^ct^6^A by the enzyme TcdA (Kang et al., 2017). However, ms^2^ct^6^A can be generated directly from ct^6^A by CDKAL1, as the necessary 2-methylthiolation and cyclization events are independent of each other (Kang et al., 2017). Further, while the cyclic modifications ct^6^A and ms^2^ct^6^A can be artificially synthesized in an oxazolone isoform, the hydantoin isoforms are the biologically relevant forms (Figure 5b) (Kang et al., 2017). ct^6^A, m^6^t^6^A, ms^2^t^6^A, ms^2^ct^6^A, ht^6^A, and g^6^A do not appear to be found in archaeal species (Boccaletto et al., 2018; Kang et al., 2017).

While archaeal tRNAs contain t^6^A and some related derivatives, ac^6^A has been hypothesized to be a structural analog of t^6^A and related molecules within archaeal tRNA (Sauerwald, Sitaramaiah, McCloskey, Söll, & Crain, 2005). ac^6^A is the most abundant modified t^6^A-like derivative in tRNA molecules from *Methanopyrus kandleri* (Sauerwald et al., 2005). Further, the t^6^A derivative hn^6^A and its relative ms^2^hn^6^A were also present in the characterization studies of ac^6^A (Reddy et al., 1992; Sauerwald et al., 2005). As was seen with ac^6^A, both hn^6^A and ms^2^hn^6^A were found in thermophilic archaeal species (Sauerwald et al., 2005). However, their roles and precise nucleotide positions within archaea tRNA remain unknown.

As with i^6^A, t^6^A and its derivatives are essential in decoding and recognizing codons. However, each t^6^A derivative has unique specificity. t^6^A and ct^6^A decode ANN (N represents any nucleotide) codons, m^6^t^6^A decodes ACY codons, ms^2^t^6^A and ms^2^ct^6^A decode AAR codons, and ht^6^A decodes the AAA codon in sea urchins and reads as asparagine instead of lysine (Kang et al., 2017; Miyauchi et al., 2013; Nagao et al., 2017). t^6^A is necessary for proper and efficient aminoacylation of tRNAs, efficient translocation, proper scanning of initiation codons, and proper read-through of stop codons (Kang et al., 2017). While t^6^A is essential in *E. coli* and many bacteria, it is not essential to *Deinococcus radiodurans*, *Thermus thermophilus*, *Synechocystis* PCC6803, and *Streptococcus mutans* (Thiaville et al., 2015). While dispensable in *D. radiodurans*, t^6^A-deficient strains of *D. radiodurans* were more sensitive to the DNA crosslink inducing drug mitomycin C and had a marked increase in protein expression of stress response genes related to protein homeostasis (Onodera, Satoh, Ohta, & Narumi, 2013; Thiaville et al., 2015). In *E. coli* and *S. mutans*, it was determined that t^6^A was a positive determinant for the PrrC toxins in both organisms (Bacusmo et al., 2017). In *S. cerevisiae*, t^6^A37 is not essential, though lack of t^6^A results in substantial growth defects. These defects include an increase in sensitivity to select stresses (i.e., heat, ethanol, and salt), an increase in sensitivity to TOR pathway inhibitors, and an increase in rates of translation at non-AUG start codons upstream of the actual translation start site (Thiaville et al., 2016). In *D. melanogaster*, a homozygous mutation of one component of the KEOPS complex results in larvae that are significantly smaller than wild type and which do not pupate, ultimately dying in larval stages (Rojas-Benitez, Thiaville, Valérie De Crécy-Lagard, & Glavic, 2015). In humans, mutations in *CDKAL1* revealed a risk association with type 2 diabetes (Kang et al., 2017). Further, mutations in several KEOPS genes result in Galloway–Mowat syndrome, a disease characterized by microcephaly and nephrotic syndrome (Arrondel et al., 2019; Braun et al., 2017; Edvardson et al., 2017). Further, t^6^A deficiency in human mitochondria due to TsaC knockout results in a MERRF-like phenotype (H. Lin et al., 2018). What additional roles or modifications may be generated from t^6^A remain an unclear, though some comparisons of t^6^A and its derivatives may be made with k^2^C and C^+^, as k^2^C and C^+^ are composed of cytidine residues that have been attached to a lysine or an arginine, respectively (Phillips & de Crécy-Lagard, 2011; Soma et al., 2003). This strategy of attaching an amino acid to the nitrogenous base of a ribonucleotide may be a relic from the RNA world (Ikeuchi et al., 2010; Soma et al., 2003) and may be used as a strategy for finding additional unknown modifications throughout biology.

### 4.5 | m^6,6^A plays an important structural role in rRNA and can be found in tRNAs

*Nucleoside modification (IUPAC abbreviation)*: *N*^6^,*N*^6^-dimethyladenosine (m^6,6^A or m^6^_2_A).

*Domain assignments*: A, B, E.

*RNA classes*: ♣, 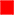.

In its capacity as a structurally important modification, m^6,6^A can be found in the final helix (helix 45) at A1518 and A1519 of the small ribosome subunit of *E. coli* and *T. thermophilus*. m^6,6^A1518 and m^6,6^A1519 are formed by RsmA (previously KsgA) in prokaryotes. In eukaryotes, the corresponding enzyme is Dim1 (Chan et al., 2011; Demirci et al., 2010; Seistrup et al., 2017). RsmA/Dim1 catalyze both dimethylation events at m^6,6^A1518 and m^6,6^A1519, which are necessary for assembling the minimal, functional ribosome (Grosjean et al., 2014; Seistrup et al., 2017). Owing to the steric interference of the dimethyl groups and the removal of hydrogen bond donors at m^6,6^A1518 and m^6,6^A1519, the normally structured tetraloop of helix 45 is weakened and results in decreased errors in translation and decreased non-AUG translation initiation (Demirci et al., 2010). However, inactivation of RsmA can occur in some organisms, resulting in impaired organismal growth and the removal of a late-stage checkpoint in 30S ribosome biosynthesis. In mammalian cells, a buildup of unprocessed small ribosomal subunits that occurs with Dim1 inactivation is lethal to the cells (Seistrup et al., 2017). However, there are a few archaeal organisms that do not have RsmA/Dim1 and have fully functional ribosomes, though RsmA/Dim1 is normally found in archaeal species (Seistrup et al., 2017). While m^6,6^A modifications were initially found in the ribosome, m^6,6^A has also been found in tRNA from the bacteria *Mycobacterium bovis*, though its location within tRNA and its function remain presently unsolved (Chan et al., 2011). Moreover, while the loss of a hydrogen bond donor in m^6,6^A is exploited in the ribosome for proper function, it remains to be seen if the loss of the hydrogen bond donor has similar effects in other RNAs, such as mRNAs and other ncRNAs.

### 4.6 | The roles of m^2^A, m^8^A, and m^2,8^A in translational fidelity and antibiotic resistance in bacteria

*Nucleoside modification (IUPAC abbreviation)*: 2-methyladenosine (m^2^A); 8-methyladenosine (m^8^A); 2,8-dimethyladenosine (m^2,8^A).

*Domain assignments*: B.

*RNA classes*: ♣ (m^2^A), 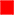.

The adenosine modifications m^2^A and m^8^A are conferred by RlmN and Cfr, respectively. In some RNAs, m^2^A and m^8^A can be further methylated at either position 8 or position 2 by Cfr or RlmN, respectively. Regardless of which singly methylated adenosine precursor is methylated, this additional methylation results in the formation of m^2,8^A. The methylation order of m^2,8^A is currently not known to be of biological significance (Yan & Fujimori, 2011). RlmN confers an m^2^A mark on tRNA species in *E. coli* at position 37 (Benítez-Páez, Villarroya, & Armengod, 2012). m^2^A37 is reported to occur in at least six *E. coli* tRNAs (tRNA^Arg^_ICG_, tRNA^Asp^_QUC_, tRNA^Gln^_cmnm5sUUG_, tRNA^Gln^_CUG_, tRNA^Glu^_mnm5s2UUC_, and tRNA^His^_QUG_) (Schwalm, Grove, Booker, & Boal, 2016). Also, m^2^A, m^8^A, and m^2,8^A can be found, separately, at A2503 in the PTC of the 23S rRNA (Yan & Fujimori, 2011). RlmN and Cfr can utilize protein-free 23S rRNA as a substrate, but the enzymes cannot utilize the fully assembled large ribosomal subunit. This observation suggests that methylation events occur during formation of the ribosome instead of after its full assembly (Yan et al., 2010). The presence of a methyl group on C2 of A2503 improves translational fidelity (Schwalm et al., 2016). The presence of a methyl group on A2503 confers resistance to more than five classes of ribosomal-targeting antibiotics (Long, Poehlsgaard, Kehrenberg, Schwarz, & Vester, 2006). As A2503 is located at the PTC of the ribosome, m^2^A may influence contacts between the exit tunnel and the nascent peptide chain and further strengthens the structure of the PTC (Polikanov, Melnikov, Söll, & Steitz, 2015; Toh, Xiong, Bae, & Mankin, 2008). Also, it is not specifically indicated if the dimethylation seen in m^2,8^A exerts both translational fidelity and antibiotic resistance effects. It is known that antibiotic resistance from C8 methylation is independent of methylation of C2 (Giessing et al., 2009). While there has not been further experimentation that has been published about these methylation marks, the methylases that confer these marks are potentially interesting antibiotic targets of interest that may be of use in future drug discovery.

## 5 | GUANOSINE-DERIVED MODIFICATIONS

### 5.1 | Alterations of the base-pairing capabilities of RNAs by m^2^G and m^2,2^G may be crucial in proper translation, while m^2^G can undergo hydroxymethylation to form hm^2^G

*Nucleoside modification (IUPAC abbreviation)*: *N*^2^-methylguanosine (m^2^G); *N*^2^, *N*^2^-dimethylguanosine (m^2,2^G or m^2^_2_G); *N*^2^-hydroxymethylguanosine (hm^2^G).

*Domain assignments*: A (not hm^2^G), B (not hm^2^G), E.

*RNA classes*: ♣ (not hm^2^G), 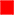.

m^2^G and m^2,2^G are two common modifications. m^2^G marks can be frequently found in archaeal and eukaryotic tRNAs but more rarely found in bacterial tRNAs at positions 10 or 26 (Table 2). However, the role of m^2^G within tRNA remains elusive (Purushothaman, Bujnicki, Grosjean, & Lapeyre, 2005; Roovers et al., 2012). While the methyl mark on m^2^G is found on the Watson–Crick face of guanosine, m^2^G base pairing to C in an RNA duplex occurs with the same energetics as G base pairing to C (Rife, Cheng, Moore, & Strobel, 1998). However, m^2^G-U Wobble base pairs are slightly more energetically stabilizing than G-U Wobble base pairs and do not affect the energetics of GNRA tetraloops (Rife et al., 1998). One methyltransferase complex that is known to form m^2^G10 marks in eukaryotes is the Trm11p/Trm112p complex (Purushothaman et al., 2005). The methyltransferase Trm-G10 was characterized in *Pyrococcus furiosus* to confer m^2^G marks, while homologs of the *trm-G10* gene were determined to be nearly ubiquitous in archaea and eukaryotes (Armengaud et al., 2004). Homologs of the Trm-G10 methyltransferase could also confer m^2,2^G marks, as seen in *Pyrococcus abyssi* (Gabant et al., 2006). A separate methyltransferase, Trm1, was found to confer m^2^G marks in *Aquifex aeolicus*, though Trm1 also conducts a second methylation event to confer m^2,2^G marks (Awai et al., 2009). In *S. cerevisiae* and other eukaryotes, the enzyme Trm1p or its homologs writes an m^2,2^G mark at G26 on numerous tRNAs (Dewe, Fuller, Lentini, Kellner, & Fu, 2017; Hopper, Furukawa, Pham, & Martin, 1982; K. Zhang et al., 2020). m^2,2^G prevents a potential G26-A44 base pair in tRNAs that could form with unmodified guanosine (Figure 4, Table 2) (Pallan, Kreutz, Bosio, Micura, & Egli, 2008). The dimethyl modification at G26 permits base pairing with any nucleotide except cytidine, thereby allowing base pairing between the m^2,2^G-D pair (D representing every nucleotide other than cytidine, not the modification dihydrouridine). m^2,2^G also prohibits the misfolding of eukaryotic tRNAs containing this modification (Figure 4) (Pallan et al., 2008).

The *E. coli* methyltransferases RlmG, RlmL, RsmC, and RsmD and their bacterial homologs are responsible for forming m^2^G in rRNA, where m^2^G occurs at five different positions in *E. coli* rRNA (Pallan et al., 2008; Sergiev, Bogdanov, & Dontsova, 2007). m^2^G marks in rRNA are hypothesized to be involved in codon–anticodon recognition, formation of the binding pocket for A-site-bound tRNA, translocation, overall ribosome structure formation, and formation of the PTC within the large ribosomal subunit (Polikanov et al., 2015; Sergiev et al., 2007). In *E. coli*, *Clostridium acetobutylicum*, and *T. thermophilus* 16S rRNA, the m^2,2^G966 mark contacts the Wobble base pair and enhances translation initiation (Burakovsky et al., 2012; Emmerechts, Barbé, Herdewijn, Anné, & Rozenski, 2007; Polikanov et al., 2015; Woese, Gutell, Gupta, & Noller, 1983).

Recently, a total RNA extraction from HEK293T cells resulted in the discovery of hm^2^G (You et al., 2019). While it is unclear which RNAs contain hm^2^G, which enzymes synthesize hm^2^G, and what additional organisms may have hm^2^G, it was discovered that hm^2^G levels are statistically higher in thyroid carcinoma samples compared to tumor-adjacent noncancerous thyroid tissue (You et al., 2019). This finding suggests that hm^2^G may be useful as a cancer biomarker, but also that cancer-related processes may drive hm^2^G expression. In humans, RNA Pol III mutations associated with hypomyelinating leukodystrophy result in an increase in the presence of m^2,2^G in tRNAs, yet also a decrease in overall tRNA transcription that can be mimicked by addition of rapamycin (Arimbasseri et al., 2015). Further, a missense variant of TRMT1, the human homolog of Trm1p, resulted in a deficiency of m^2,2^G marks in tRNAs and may be linked to intellectual disability, developmental delay, and epilepsy in some patients (K. Zhang et al., 2020). Additional studies in this area of human health and in discovering the functionalities of m^2^G, m^2,2^G, and hm^2^G in other organisms may yield additional functionalities of these modifications and their cognate enzymes.

### 5.2 | The many roles of m^7^G: from capping mRNAs and presence in mRNAs, to their presence in rRNAs and tRNAs, to forming the precursor molecule for m^2,7^G and m^2,2,7^G within spliceosomal snRNAs

*Nucleoside modification (IUPAC abbreviation)*: 7-methylguanosine (m^7^G); *N*^2^,7-dimethylguanosine (m^2,7^G); *N*^2,2^-7-trimethylguanosine (m^2,2,7^G).

*Domain assignments*: A, B (m^7^G), E.

*RNA classes*: ♣ (m^7^G), 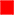 (m^7^G), 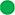 (m^7^G), 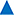.

5′-capping of mRNAs is usually accomplished by the addition of an m^7^G to the 5′-most nucleotide of a nascent mRNA transcript. The eukaryotic m^7^G-containing cap is used in the recruitment of pre-mRNA splicing and initiation factors to further mature mRNA transcripts. The eukaryotic m^7^G-containing cap is added co-transcriptionally to the 5′ end of newly transcribed mRNA in an unusual inverted 5′-5′ triphosphate linkage with a GTP molecule that is subsequently methylated by RNMT or its homologs (reviewed in Galloway & Cowling, 2019; Topisirovic, Svitkin, Sonenberg, & Shatkin, 2011). Once the m^7^G cap is added, the next first nucleotide in the nascent transcript is methylated to contain an Nm mark (Akichika et al., 2019; Boulias et al., 2019; Sendinc et al., 2019). The cap also allows for further maturation of mRNAs by polyadenylation and eventually nuclear export. Once successfully spliced and formed as mRNA, cap-dependent initiation of protein synthesis of mRNAs occurs. Further, this cap is used to protect mRNAs from 5′-to-3′ exoribonuclease activity and degradation of mRNAs, while also looping mRNAs throughout translation (Ramanathan, Robb, & Chan, 2016). While m^7^G is found in the majority of 5′ caps in eukaryotic mRNAs, caps exist that are formed from chemical groups other than m^7^G. These include NAD^+^, NADH, and dpCoA (Bird et al., 2016; Cahová, Winz, Höfer, Nübel, & Jäschke, 2015; Y. G. Chen, Kowtoniuk, Agarwal, Shen, & Liu, 2009; Julius & Yuzenkova, 2017; Kowtoniuk, Shen, Heemstra, Agarwal, & Liu, 2009).

Until 2019, m^7^G in mRNAs was thought to be found exclusively within the 5′ cap of mRNAs. However, m^7^G marks have since been identified in the CDS, 3′ UTR, and more rarely in the 5′ UTR of human mRNAs (L.-S. Zhang, Liu, et al., 2019). Additionally, m^7^G marks have been found in human ncRNAs (McCown et al., 2019; L.-S. Zhang, Liu, et al., 2019). At least 90 internal m^7^G methylation sites have been identified in human mRNA, with high degrees of conservation of the m^7^G marks found across murine and human cell lines (L.-S. Zhang, Liu, et al., 2019). While m^7^G marks occur at several motifs within mRNA (AGm^7^GA, GGm^7^GAA, AUCGm^7^GA, and UUm^7^GAU), the enzyme or enzymes that confer these marks remain uncharacterized (L.-S. Zhang, Liu, et al., 2019).

In bacteria and *S. cerevisiae*, m^7^G marks in tRNAs are conferred by Trm8p/Trm82p. The m^7^G mark at m^7^G46 extends tRNA half-life (Alexandrov et al., 2006). In archaeal tRNAs, m^7^G is present but the precise nucleotide location and tRNA identity are unknown (Edmonds et al., 1991; Tomikawa, 2018). Within pre-rRNA transcripts in eukaryotes, m^7^G marks are catalyzed by Bud23 (*S. cerevisiae*) (Létoquart et al., 2014) or WBSCR22 (human) (Zorbas et al., 2015). In humans, m^7^G marks are required for proper nuclear export of premature 18S ribosome complexes (Sloan et al., 2017), while rRNA is modified in the cytosol in *S. cerevisiae* (Létoquart et al., 2014).

Further hypermethylation of the guanosine in the 5′ m^7^G cap can occur, as is the case in eukaryotic spliceosomal snRNAs, some snoRNAs, and telomerase RNA (Monecke, Dickmanns, & Ficner, 2009). Sequential methylation of the m^7^G cap via Tgs1 gives rise to m^2,7^G, then m^2,2,7^G (Monecke et al., 2009). The trimethylated guanosine residue marks the maturation of spliceosomal RNAs, which has further roles in enhancing the nuclear receptor signal transduction cascade, in enhancing transcriptional regulation, and in mitigating spinal muscular atrophy (reviewed in Monecke et al., 2009). While it remains unknown whether these modifications affect the structural stability of mRNAs, m^7^G and its derivatives have a few roles in human health. Overexpression of RNMT, which stimulates mRNA cap formation, is sufficient to induce tumor formation in human mammary epithelial cells (reviewed in Galloway & Cowling, 2019). Additional discoveries in characterizing writers of m^7^G may yield further insights into the significance of the m^7^G marks in mRNAs and in human health.

### 5.3 | Biosynthetic pathways of Q and G^+^ and their function in tRNAs

*Nucleoside modification (IUPAC abbreviation)*: pre-queuosine_0_ (preQ_0_); pre-queuosine_1_ (preQ_1_); epoxyqueuosine (oQ); queuosine (Q); galactosyl-queuosine (GalQ); mannosyl-queuosine (ManQ); glutamyl-queuosine (GluQ); archaeosine (G^+^).

*Domain assignments*: A (preQ_0_ and G^+^), B (not G^+^, ManQ, and GalQ), E (not preQ_0_, preQ_1_, oQ, G^+^, and GluQ).

*RNA classes*: ♣.

**FIGURE 6:**
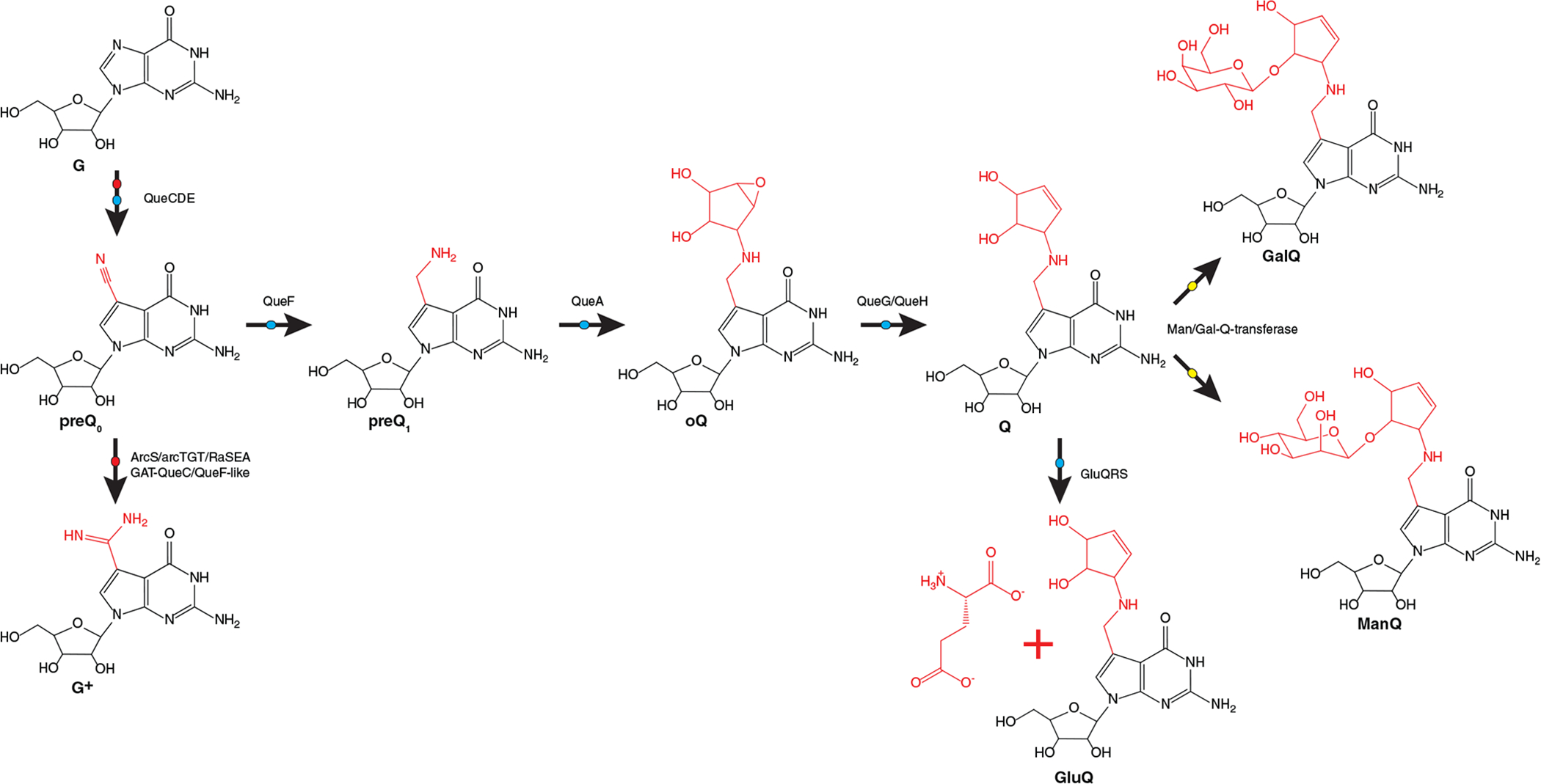
Scheme for queuosine derivatives. Pathway depicts the modifications that occur during the production of queuosine derivatives preQ_0_, preQ_1_, oQ, Q, GalQ, ManQ, GluQ, and G^+^. Red color indicates the modification added to the guanosine molecule. Enzymes that catalyze each reaction are adjacent to arrow. The plus sign by GluQ indicates that glutamate is incorporated onto one of the hydroxyl groups from Q, though it is unknown at present which of the two hydroxyl groups receives the glutamate. Arrow tails containing red, blue, or yellow ellipses denote reactions that occur in archaea, bacteria, or eukaryotes, respectively

Q and G^+^ represent two modified ribonucleosides that are derived from a split in a central biosynthetic pathway in archaea and bacteria, following the processing of guanosine into preQ_0_ via the QueCDE enzyme complex (Figure 6) (Iwata-Reuyl, 2008; Reader, Metzgar, Schimmel, & de Crécy-Lagard, 2004). In bacterial species, the nitrile group on the preQ_0_ molecule is reduced into an amine to form preQ_1_ by the QueF enzyme (Van Lanen et al., 2005). The resulting preQ_1_ molecule is incorporated into a tRNA molecule at position 34 by TGT synthase (Iwata-Reuyl, 2008; Reader et al., 2004). Once incorporated, the preQ_1_ moiety on tRNA is further modified to contain a cyclopentane ring with two hydroxyl groups and an epoxy ring into oQ34 by the enzyme QueA. The epoxide is removed from oQ34 by the enzyme QueG to form Q34 (Miles, McCarty, Molnar, & Bandarian, 2011; Zallot, Ross, et al., 2017). Separately, Q34 was also determined to be the product of a reaction catalyzed by a nonhomologous enzyme, QueH (Miles et al., 2011; Zallot, Ross, et al., 2017). Once Q34 is formed, Q34 can be conjugated to a mannose (ManQ) or galactose (GalQ) via a Man/Gal-Q-transferase in eukaryotes, respectively (Okada & Nishimura, 1977). Q34 can also be further modified to contain a glutamine (GluQ) moiety via the bacterial GluQRS enzyme (Campanacci et al., 2004; Ehrenhofer-Murray, 2017; Salazar, Ambrogelly, Crain, McCloskey, & Soll, 2004). In some archaeal species, preQ_0_ is incorporated into a tRNA molecule at G15 and subsequently modified to become G^+^15 by the enzymatic complex consisting of the ArcS/arcTGT enzyme and the RaSEA enzyme (Mohammad et al., 2017; Phillips et al., 2010; Yokogawa et al., 2019). However, numerous Crenarchaeota species lack ArcS and could instead form G^+^ with either the GAT-QueC or QueF-like protein families (Phillips et al., 2012).

While most bacteria and eukaryotes contain Q or its derivatives, Q biosynthesis genes are not found in many bacterial species and are absent in eukaryotes. In bacteria that cannot synthesize Q and in eukaryotes, Q or its corresponding nucleobase queuine are imported or salvaged. One class of bacterial transport proteins, known as COG1738, QueT, or YhhQ, is involved in transporting preQ_0_ or preQ_1_ (reviewed in McCown, Liang, Weinberg, & Breaker, 2014; Zallot, Yuan, & De Crécy-Lagard, 2017). Three additional transport proteins in *Clostridioides difficile* were also characterized in transporting preQ_1_, queuine, and Q (Yuan et al., 2019). Owing to the incredible metabolic demand to synthesize Q, at least two salvage pathways exist to salvage or scavenge Q from the gut microbiota. The first bacterial pathway involves homologs of the YhhQ and TGT enzymes that transport or utilize queuine as a substrate (Yuan et al., 2019). The second bacterial pathway utilizes a queuosine hydrolase to generate queuine from queuosine; the cyano group in queuine is then converted into an aminomethyl group, forming preQ_1_ via a queuine lyase (Yuan et al., 2019). However, it is presently unknown how queuine or other Q-related molecules are transported into eukaryotes. Regardless of how Q or related molecules enter into or are synthesized by organisms, the presence of Q34 in tRNAs improves their ability to read degenerate codons (Meier, Suter, Grosjean, Keith, & Kubli, 1985). The absence of enzymes that incorporate Q into tRNA or enzymes that synthesize preQ_1_ have different deleterious phenotypes in a wide array of bacterial and eukaryotic organisms (Durand, Dagberg, Uhlin, & Bjork, 2000; Rakovich et al., 2011). Further, in *S. pombe* and in other eukaryotes, Q-containing tRNA^Asp^ further enhances rates of m^5^C38 formation, while enhancing the translation speed for C-ending codons and reducing the translation speed for U-ending codons (Müller et al., 2019; Tuorto et al., 2018). Separately, G^+^ is used in the D-loops of archaeal tRNAs to stabilize the overall tRNA structure (Iwata-Reuyl, 2008; Lorenz et al., 2017).

Several of the Q-derived modifications have effects on the health or functionality of organisms that use Q. preQ_1_ and Q biosynthesis genes are required for effective nitrogen-fixing symbiosis between *Sinorhizobium meliloti* and the barrel clover plant (Marchetti et al., 2013). Q deficiency halts tyrosine production in germ-free mice (Rakovich et al., 2011). In several human and murine cancer cell lines, lack of Q modifications in tRNAs has been associated with cell proliferation, anaerobic metabolism, and malignancy (Elliott, Katze, & Trewyn, 1984; Pathak, Jaiswal, & Vinayak, 2008). The presence of Q was shown to directly affect m^5^C levels in *S. pombe* tRNA^Asp^ (Ehrenhofer-Murray, 2017). Further, a queuosine-deficient diet results in endoplasmic reticulum stress and an unfolded protein response in HeLa cells and in germ-free mice (Tuorto et al., 2018). Additionally, Q-containing tRNAs in HeLa cells were recently demonstrated to protect tRNA fragmentation from oxidative stress and angiogenin (Xiaoyun Wang et al., 2018). As tRNA fragments generate functional small RNA molecules similar to miRNAs, this protective mechanism may be helpful to cellular survival or a viable drug target in oncology studies (Xiaoyun Wang et al., 2018). GluQ has been linked to effective stress response in *Shigella flexneri* (Caballero et al., 2012). While previous experimental work has determined the biosynthetic enzymes involved in Q and G^+^ biosynthesis, the roles of ManQ and GalQ remain unknown. However, further appreciation of Q as a micronutrient (Fergus, Barnes, Alqasem, & Kelly, 2015) may further elucidate the roles of these modified Qs and other molecules in this pathway.

### 5.4 | m^1^G has effects in neurodegenerative diseases and glucose metabolism

*Nucleoside modification (IUPAC abbreviation)*: 1-methylguanosine (m^1^G).

*Domain assignments*: A, B, E.

*RNA classes*: ♣, 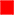.

m^1^G occurs in several paradigms. m^1^G acts as a precursor to yW and imG, as discussed below. At G9 in tRNAs from all domains of life, the enzyme Trm10 or homologous enzymes confer the m^1^G mark (Howell et al., 2019; Jackman, Montange, Malik, & Phizicky, 2003). Separately, m^1^G can also be found at G37 in tRNAs. While m^1^G37 occurs in archaeal and eukaryotic tRNAs as a precursor for yW and imG, TrmD can also confer an m^1^G37 mark in bacteria, though this mark is not further modified (Goto-Ito, Ito, & Yokoyama, 2017). Despite this research on m^1^G marks in tRNAs, it is unclear what effects m^1^G has on tRNA structure. In the *E. coli* 23S ribosome, an m^1^G mark was found at G745, which supports proper polysome formation on mRNAs and sensitizes the *E. coli* ribosome to the ribosome-binding drug viomycin (Gustafsson & Persson, 1998; Hsuchen & Dubin, 1980; Ishiguro, Arai, & Suzuki, 2019). The enzyme that confers the m^1^G745 mark was determined to be RlmA (Gustafsson & Persson, 1998). There are biologically relevant phenotypes that are associated with m^1^G. In *S. cerevisiae*, deletion strains of Trm10 are hypersensitive to 5-fluorouracil (Krishnamohan & Jackman, 2017). In humans, two mutations in hTRMT10A were determined to be causative in neurodegenerative defects and in glucose metabolism disorders (Krishnamohan & Jackman, 2017). Further effects of m^1^G in a biological context may further elucidate previously unknown mechanisms of neuron formation and may be of significance in central metabolism.

### 5.5 | The yW and imG biosynthesis pathways start with m^1^G biosynthesis

*Nucleoside modification (IUPAC abbreviation)*: 4-demethylguanosine (imG-14); wyosine (imG); isowyosine (imG2); methylwyosine (mimG); 7-aminocarboxypropyl-demethylwyosine (yW-86); 7-aminocarboxypropylwyosine (yW-72); 7-aminocarboxypropylwyosine methyl ester (yW-58); wybutosine (yW); undermodified hydroxywybutosine (OHyW*); methylated undermodified hydroxywybutosine (OHyWy); peroxywybutosine (o_2_yW); hydroxywybutosine (OHyW).

*Domain assignments*: A (not yW-58, yW, OHyW*, OHyWy, o_2_yW, and OHyW), E (not imG2 and mimG).

*RNA classes*: ♣.

**FIGURE 7:**
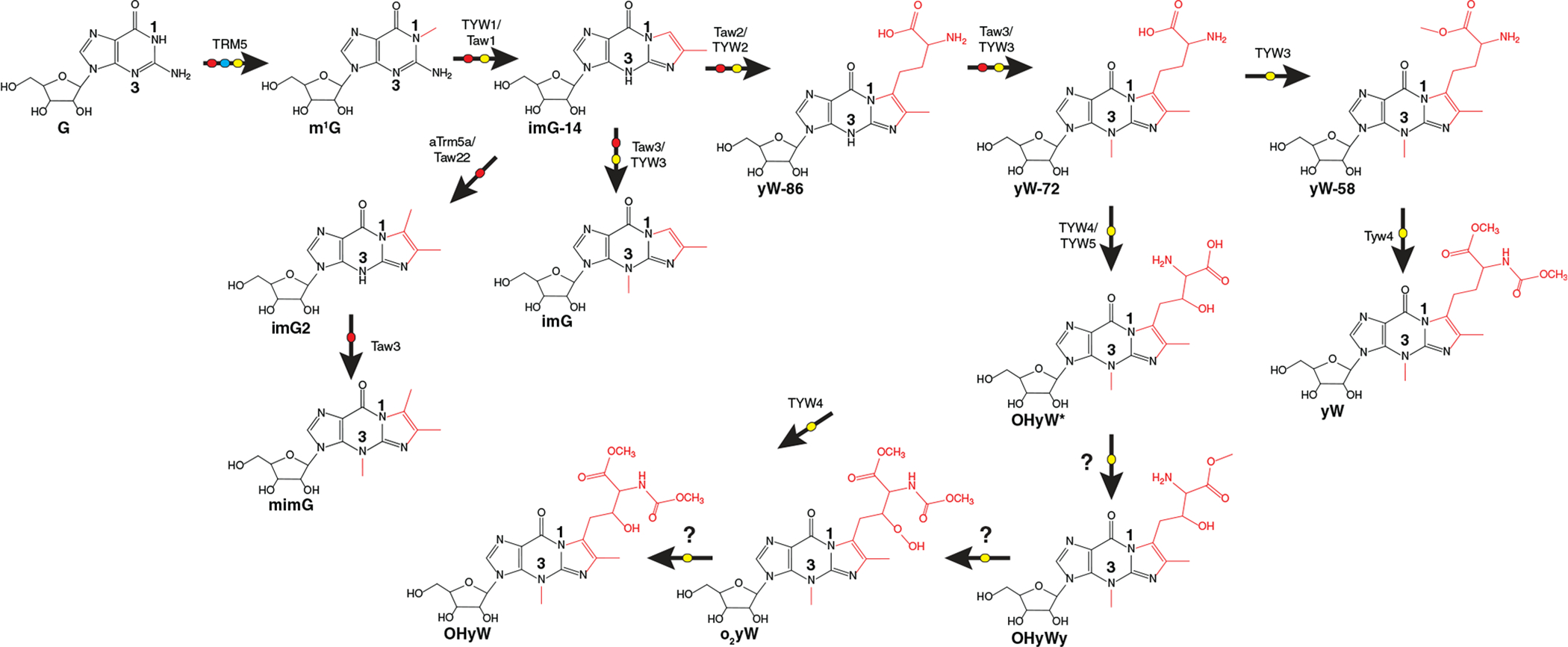
Scheme for wybutosine derivatives. Chemical modifications (red) that occur in the production of wybutosine derived from guanosine (G) include: m^1^G, imG-14, imG, imG2, mimG, yW-86, yW-72, yW-58, yW, OHyW*, OHyWy, o_2_yW, and OHyW. Enzymes that catalyze each reaction are adjacent to arrow, otherwise a question mark (?) indicates enzyme is unknown. Arrow tails containing red, blue, or yellow ellipses denote reactions that occur in archaea, bacteria, or eukaryotes, respectively. The bolded numbers 1 and 3 are added to indicate position N1 and N3, respectively, in guanosine

yW and related analogs are essential in the stabilization of codon–anticodon pairing in tRNA^Phe^ within eukaryotic and archaeal species (Noma, Kirino, Ikeuchi, & Suzuki, 2006). Synthesis of yW and imG represents a complicated biosynthesis pathway within many archaeal and eukaryotic organisms (Figure 7). The biosynthesis pathway begins with the formation of m^1^G at G37 in tRNA^Phe^, as catalyzed by the TRM5 protein (Urbonavičius, Meškys, & Grosjean, 2014). The subsequent reactions are catalyzed by TYW1–4 proteins in eukaryotes and Taw1–3 proteins in Euryarchaeota (Noma et al., 2006; Urbonavičius et al., 2014). Further, the modifications imG-14, yW-86, yW-72, and yW-58 are abbreviated based on how many fewer Daltons of mass are observed with these modifications than imG or yW (Noma et al., 2006). For example, imG-14 has 14 Da less mass than imG.

Using TYW1/Taw1 enzymes, a ring closure occurs on the nucleobase of m^1^G, forming imG-14 (Figure 7). From here, there are no fewer than three outcomes of imG-14, due to the genetic differences in archaeal and eukaryotic species. For eukaryotes (e.g., such as *S. cerevisiae*) and archaea of the phylum Euryarchaeota, imG-14 is methylated by TYW3/Taw3 at N3 into imG or attached to an α-amino-α-carboxypropyl group from SAM by TYW2/Taw2 to form yW-86. yW-86 can then be methylated at N3 into yW-72. Euryarchaeota do not appear to conduct any further reactions on yW-72. All eukaryotes can carboxymethyate yW-72 to form yW-58 with the TYW3 enzyme. yW-58 can then be carboxymethylated again by the enzyme TYW4 to form yW. However, in higher order eukaryotes, yW-72 can also be hydroxylated into OHyW* by the TYW4 and TYW5 enzymes. OHyW* is then further carboxymethylated into OHyW by TYW4 (M. Kato et al., 2011; Noma et al., 2010). While the synthesis reactions involving OHyWy and o_2_yW have not been determined, these molecules may be precursors of OHyW. Separately, Crenarchaeota conduct a completely different set of enzymatic reactions on imG-14. Instead, imG-14 is methylated at a carbon placed next to position N1 by the enzymes aTrm5a/Taw22 to form imG2. Taw3 can subsequently methylate imG2 at position N3 to form mimG (Urbonavičius et al., 2014). Some of these enzymes or the overall yW biosynthetic process may be of significance to biology, as overexpression of the TYW2 gene was found in breast cancer cell lines. However, yW-containing modifications were not significantly increased in tRNA modifications (Rodriguez et al., 2012). This possibly means that TYW2 has additional functions in breast cancer that are presently unknown (Rodriguez et al., 2012). Lack of yW modifications in tRNA^Phe^ may cause an activation of the replication of HIV RNA and human T-cell lymphotrophic virus 1 due to enhancing codon frameshifting in cells (Landgraf, McCarthy, & Booker, 2016; Y. Suzuki, Noma, Suzuki, Ishitani, & Nureki, 2009). There have been instances where a lack of yW modifications has been correlated with rat and mouse tumors and Ehrlich ascites in mice, and represent a point of interest in future oncology research (reviewed in Landgraf et al., 2016). At present, none of the yW-related modifications themselves are found to be directly culpable in any human diseases, though they may have additional functions that are presently unknown. Moreover, given the complexity of the yW synthesis pathway, it is also possible that additional yW-derived modifications exist which may reveal yW derivatives in bacteria or may add to further complexity of yW biosynthesis.

## 6 | CYTIDINE-DERIVED MODIFICATIONS

### 6.1 | The dual lives of m^5^C, hm^5^C, and f^5^C as DNA- and RNA-based modifications

*Nucleoside modification (IUPAC abbreviation)*: 5-methylcytidine (m^5^C); 5-hydroxymethylcytidine (hm^5^C); 5-formylcytidine (f^5^C).

*Domain assignments*: A (m^5^C), B (m^5^C), E.

*RNA classes*: ♣ (m^5^C and f^5^C), 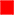 (m^5^C), 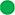 (m^5^C and hm^5^C), 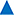 (m^5^C).

While the DNA forms of m^5^C, hm^5^C, and f^5^C have significant roles in epigenetics (Hashimoto, Zhang, Vertino, & Cheng, 2015), the RNA forms of these nucleosides have distinct roles (Cui et al., 2017; Delatte et al., 2016; Nakano et al., 2016; Trixl & Lusser, 2019; R. Wang et al., 2016). m^5^C can be found in archaeal and eukaryotic tRNAs (Dunn, 1960; Helm, 2006; Motorin & Helm, 2010), rRNAs from all three domains of life (Edelheit, Schwartz, Mumbach, Wurtzel, & Sorek, 2013; Squires & Preiss, 2010), archaeal and eukaryotic mRNAs (D. T. Dubin & Taylor, 1975; Edelheit et al., 2013), human lncRNAs (Amort et al., 2013; McCown et al., 2019), human vault RNAs (Hussain et al., 2013; Squires et al., 2012), and in the HIV-1 viral genome (Courtney et al., 2019). The TRM4B protein was determined to have m^5^C RNA methyltransferase activity in *S. pombe* and *Arabidopsis* (Cui et al., 2017). NSUN1–6 and other close homologs have also been attributed to the genesis of m^5^C in numerous eukaryotes, including humans (Heissenberger et al., 2019; Van Haute, Powell, & Minczuk, 2017).

In tRNAs, it has been proposed that the formation of m^5^C48 contributes to the L-shape of tRNAs in vivo (Väre, Eruysal, Narendran, Sarachan, & Agris, 2017). m^5^C2278 in the 25S rRNA of *S. cerevisiae* is one modification that serves as an anchor for the ribosomal protein eL41. Once secured by m^5^C2278, eL41 forms a bridge between the large and small ribosomal subunits (Sharma & Lafontaine, 2015). Loss of m^5^C2278 in *S. cerevisiae* results in oxidative stress-induced refolding of rRNA (Schosserer et al., 2015). Recently, it was discovered that NSUN5 confers an m^5^C mark on C3782 in human 28S rRNA, while the mouse homolog of NSUN5 confers a related m^5^C mark on C3438 in mouse 28S rRNA (Heissenberger et al., 2019). It appears that the m^5^C3782 and m^5^C3438 marks in human and mice 28S rRNA are analogous to m^5^C2278 in *S. cerevisiae* ribosomes. Likewise, m^5^C3782 in human 28S rRNA is necessary for anchoring the eL41 protein, which then forms a bridge between the large and small ribosomal subunits (Heissenberger et al., 2019). While the precise number of m^5^C sites in mRNAs is currently under debate, its structural roles are less well-understood (reviewed in Trixl & Lusser, 2019). In *Arabidopsis*, m^5^C is present in mRNAs that are associated with low translational activity during the early stages of plant development, while mutated TRM4B proteins result in defects in root development and a decreased abundance of m^5^C (Cui et al., 2017). In lncRNAs, m^5^C in the A-region of *Xist* interferes with binding to PRC2 via an unknown mechanism (Amort et al., 2013). m^5^C in vault RNAs is necessary for the maturation of smaller miRNA-like RNAs, but it is unclear if m^5^C confers additional functionalities to vault RNAs (Hussain et al., 2013).

hm^5^C and f^5^C are oxidized from m^5^C in humans by TET or ABH1 enzymes (Van Haute et al., 2017; R. Wang et al., 2016). Though it is unknown which enzymes generate hm^5^C, this modification is found in archaea and bacteria (Huber et al., 2015). hm^5^C-containing mRNAs have increased rates of translation in vitro, while also promoting brain development in *D. melanogaster* (Delatte et al., 2016). However, the recent discovery of hm^5^Cm has suggested that the antibodies used in identifying hm^5^C marks may not be able to distinguish between hm^5^C or hm^5^Cm, as the anti-hm^5^C antibody was not validated for being incapable of binding to hm^5^Cm (Huber, van Delft, Tanpure, Miska, & Balasubramanian, 2017). f^5^C is necessary for deciphering the tRNA^Met^ that aids in translating the AUA and AUG codons in mitochondrial peptide synthesis (Van Haute et al., 2017).

Lack of appropriate methylation of C at target sites and/or oxidation of m^5^C marks results in mitochondrial-associated diseases, Leber’s hereditary optic neuropathy, myopathy, and hypotonia (reviewed in Van Haute et al., 2017). These diseases occur concordantly with knockdown of either *nsun3* or *abh1* genes (Haag et al., 2016; Van Haute et al., 2017). A knockout of *nsun5* results in a decrease in mouse weight, lean mass, and overall protein synthesis, while a depletion of NSUN5 in cancer cells results in a reduction of cell proliferation and size due to aberrant ribosome formation (Heissenberger et al., 2019). Further, a heterozygous microdeletion within chromosome 7 that includes *nsun5* results in Williams–Beuren syndrome, characterized by cardiac defects, lack of weight gain, and mental deficits (Heissenberger et al., 2019). Deletion or mutation of *nsun2* in T cells results in a loss of m^5^C marks on HIV-1 genomic RNA that consequently inhibits HIV-1 mRNA translation, alternative splicing of viral RNAs, and viral replication (Courtney et al., 2019). Further research into the roles of m^5^C in mRNAs, the roles of hm^5^C and f^5^C, and the impact of the enzymes that generate these marks may glean further insights into areas of significant impact on human and organismal biology.

### 6.2 | m^3^C marks are formed by four methyltransferases

*Nucleoside modification (IUPAC abbreviation)*: 3-methylcytidine (m^3^C).

*Domain assignments*: E.

*RNA classes*: ♣, 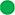.

m^3^C was first discovered in *S. cerevisiae* tRNA (Hall, 1963; Iwanami & Brown, 1968; Maden & Salim, 1974). Subsequently, m^3^C was found to be synthesized by at least four enzymes, with each enzyme having specificity for a particular RNA class (Noma et al., 2011; L. Xu et al., 2017). METTL2 and METTL6 methylate C32 on tRNA^Thr(UGU)^, tRNA^Arg(CCU)^, and several tRNA^Ser^ eukaryotic isoacceptors (Noma et al., 2011; L. Xu et al., 2017). METTL6 interacts with seryltRNA synthetase in an RNA-dependent fashion, which may mean that METTL6 is involved in the methylation of serine tRNA isoacceptors (L. Xu et al., 2017). However, only METTL8 can form m^3^C marks on mRNA in murine and human cell lines (L. Xu et al., 2017). ALKBH1 was subsequently identified as a demethylase for m^3^C marks on mRNAs (Ma, Ding, Ye, Yuan, & Feng, 2019). Further, knockouts of *mettl2*, *6*, and *8* did not demonstrate any observable developmental defects in mice (L. Xu et al., 2017). However, it is possible that deficiencies in METTL8 increase ribosome stalling in colon cancer cells (L. Xu et al., 2017). METTL2 and METTL6 account for only 20–40% of m^3^C in tRNAs from HeLa and HCT116 cells and mouse liver extracts, indicating that there are likely more than two enzymes that methylate cytidine residues in tRNA (L. Xu et al., 2017). As METTL8 accounts for ~100% of m^3^C marks in mRNAs from HeLa and HCT116 cells, it is very likely that METTL8 may be the major or the only methyltransferase that confers m^3^C marks in mRNAs (L. Xu et al., 2017). Additionally, m^3^C levels in mRNAs were lower in hepatocellular carcinoma biopsied tissues compared to noncancerous tissues, indicating m^3^C is a potential liver cancer diagnostic marker (Ma et al., 2019). Moreover, the same study noted that m^3^C levels in cancerous and noncancerous tissues were significantly higher than m^3^C levels in cultured cancerous and noncancerous human cell lines that they had examined (Ma et al., 2019), which may indicate biological roles involving m^3^C may be missed in only examining human cell lines.

Above, A-to-I editing was discussed as one type of RNA editing. Another type of RNA editing is C-to-U editing, which can use m^3^C as an intermediate modification. In *Trypanosoma brucei*, the enzyme TRM140 methylates C32 into m^3^C in tRNA^Thr(AGU)^ as part of a pathway to convert C32 to U32 (Rubio et al., 2017). Co-expression of TRM140 and ADAT2/3 prevents wholesale deamination of the trypanosomal genome, while also providing a key insight into the functionality of AID in antibody class diversification (Rubio et al., 2017). While C-to-U editing has been observed in several eukaryotes and archaea, it is unclear whether the trypanosomal process involving m^3^C is present in these organisms (Rubio et al., 2017). While the structural roles of m^3^C in RNA remain unstudied, additional studies in how m^3^C levels can be used as a cancer diagnostic and whether m^3^C is involved in C-to-U editing in other organisms remains two areas of research that may elucidate more functions and unknown biology involved with m^3^C.

### 6.3 | s^2^C allows for anticodon flexibility and enhanced codon binding in bacterial and archaeal tRNAs and can be methylated to form ms^2^C

*Nucleoside modification (IUPAC abbreviation)*: 2-thiocytidine (s^2^C); 2-methylthiocytidine (ms^2^C).

*Domain assignments*: A (not ms^2^C), B.

*RNA classes*: ♣.

s^2^C is present in archaeal and bacterial tRNAs, specifically tRNA^Ser(GCU)^ and three tRNA^Arg^ molecules (Jager, Leipuviene, Pollard, Qian, & Bjork, 2004; Shigi, 2014). The sulfur for s^2^C is derived from cysteine via the IscS enzyme, which serves as a central sulfur hub. IscS then moves the cysteine-derived sulfur to the IscU enzyme and then the TtcA enzyme. The thiolated TtcA incorporates sulfur into position C32 on tRNA, generating s^2^C32 (Shigi, 2014). While there were no observed growth deficits found in mutagenized strains of *E. coli* or *Salmonella enterica* that lacked TtcA, s^2^C modifications were determined to have a role in increasing the selection rate by ~30% of the ternary complex of aminoacylated-tRNA/EF-Tu/GTP within the A-site of the ribosome (Jager et al., 2004). Further, s^2^C in tRNA interferes with codon recognition in the A-site of the ribosome and decreases the translation rate of some mRNAs (Jager et al., 2004). Lack of s^2^C corresponds to an increase in frameshifting rates during translation (Jager et al., 2004). Additional studies have determined that s^2^C modifications allow for flexibility in the anticodon stem and loop structures, which is necessary for select tRNAs to properly decode mRNAs (Cantara et al., 2012).

ms^2^C was recently discovered in *E. coli* and *P. aeruginosa* tRNAs that had been previously determined to have s^2^C marks (Reichle, Petrov, Weber, Jung, & Kellner, 2019). While it does not appear that there are any known methyltransferases that confer the ms^2^C mark, it appears that the modification is the result of methylating agents or damage that occurs on tRNAs (Reichle et al., 2019). At present, it does not appear that all s^2^C marks have the capability of being methylated. Moreover, AlkB seems to be at least one enzyme that can revert the ms^2^C mark back to s^2^C (Reichle et al., 2019). However, there is a separate AlkB-independent process that removes the 2-methylthio group and rethiolates C to form s^2^C, though it is unclear how this process occurs (Reichle et al., 2019). ms^2^C marks may reduce the structural flexibility in tRNAs that would otherwise have the s^2^C mark and the presence of ms^2^C marks in tRNA reduces translational efficiency (Reichle et al., 2019). With the presence of ms^2^C and the extensive modifications featuring s^2^U below, it is curious to see if a similar myriad of modifications can also be found with thiolated cytidine nucleotides.

### 6.4 | m^4^C, m^4^Cm, ac^4^C, and ho^5^C are within rRNA and m^4,4^C is present in viral-infected human cells

*Nucleoside modification (IUPAC abbreviation)*: *N*^4^-methylcytidine (m^4^C); 4,2′-*O*-dimethylcytidine (m^4^Cm); *N*^4^,*N*^4^,2′-*O*-trimethylcytidine (m^4,4^Cm); *N*^4^-acetylcytidine (ac^4^C); 5-hydroxycytidine (ho^5^C); *N*^4^,*N*^4^-dimethylcytidine (m^4,4^C).

*Domain assignments*: A (m^4,4^Cm), B (m^4^C, m^4^Cm, and ho^5^C), E (ac^4^C, m^4,4^C, m^4,4^Cm).

*RNA classes*: ♣ (ac^4^C, m^4,4^Cm), 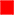 (not m^4,4^Cm), 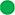 (ac^4^C).

Extensive research on the DNA form of *N*^4^-methylcytidine (m^4^C) has shown its involvement in the epigenetic regulation of genes (W. Chen, Yang, Feng, Ding, & Lin, 2017). However, current knowledge of the RNA form of m^4^C appears to be limited to its presence within bacterial rRNA and its writer, the RsmH enzyme (Kimura & Suzuki, 2010). The initial discovery of m^4^C was considered a possible side effect of treating RNA samples with acid, with m^4^Cm possibly being the native modified ribonucleoside (Limbach, Crain, & McCloskey, 1994). However, subsequent analysis indicated that the RsmH protein is a methyltransferase that methylates the N4 position of C1402 in the *E. coli* 16S rRNA. The enzyme RsmI encodes for a methyltransferase that confers the 2′-*O*-methyl mark to make m^4^Cm at C1402 (Kimura & Suzuki, 2010). Subsequently, both the m^4^C1402 and m^4^Cm1402 modifications were determined to prevent non-AUG initiation in *E. coli* by decreasing read-through effects of the UGA stop codon (Jiang, Seo, & Chow, 2016; Kimura & Suzuki, 2010). m^4^Cm1402 in the 30S small ribosomal subunit stabilizes helix 44 and may assist in interactions between the ribosome, P-site tRNA docking, and mRNA interactions (Jiang et al., 2016). Determining how widespread m^4^C and m^4^Cm marks are in bacteria may be helpful in determining if the RsmH or RsmI proteins can be a viable option for antibiotic targeting. Separately, m^4,4^Cm has been identified in the tRNAs of several archaeal species (Noon et al., 2003).

Previously, ac^4^C exists within several tRNAs and the 18S rRNA of eukaryotes and both marks are formed by the enzyme Tan1 (Johansson & Byström, 2004; Parthasarathy, Ginell, De, & Chheda, 1978). Deletion of *tan1* in *S. cerevisiae* results in a decrease in A-site occupancy of the ribosome (Chou, Donnard, Gustafsson, Garber, & Rando, 2017). However, P-site occupancy rates increase (Chou et al., 2017). It was hypothesized that ac^4^C could have roles in positioning peptidyl-tRNA molecules within the ribosome, enhancing the translocation of codons of mRNA from the P-site to the E-site of the ribosome, or in delaying the progression of mRNA when translocating from the A-site to the P-site (Chou et al., 2017). This hypothesis was confirmed in an RNA-seq-based analysis of the transcriptome-wide presence of ac^4^C marks in HeLa cells (Arango et al., 2018). The enzyme NAT10 was determined to catalyze the formation of ac^4^C in a variety of mRNAs, where the presence of these marks in mRNAs was shown to promote translation and mRNA stability (Arango et al., 2018). It remains to be seen if the ac^4^C mRNA modifications exist in other eukaryotes or if this modification is seen in bacteria or archaea. Also, it is possible that NAT10 may present a viable anticancer therapeutic target and could be pursued in further research.

Like m^4^C, ho^5^C has a DNA counterpart and is studied in the context of C-to-T transition mutations in DNA (Zahn, Averill, Wallace, & Doublié, 2011). The enzyme that synthesizes the RNA form of ho^5^C is RlhA and AroD, which generates ho^5^C from chorismate and iron–sulfur clusters (Kimura, Sakai, Ishiguro, & Suzuki, 2017; Popova & Williamson, 2014). The role of ho^5^C in biology was obscured until recently. ho^5^C was identified at position 2501 in the 23S rRNA of the *E. coli* ribosome and at an equivalent position within the 23S ribosome within *Deinococcus radiodurans* (Havelund et al., 2011). As ho^5^C2501 localizes to the PTC and forms a base triple that interacts with P-site tRNA, it is quite likely that the structural function of ho^5^C2501 plays significant roles in translation (Kimura et al., 2017). As methods to extract ho^5^C from nascent RNA would often obscure the modification, the presence of this modification in other RNA classes should be re-examined (Havelund et al., 2011). The function of ho^5^C in the ribosome appears to have some connection with environmental iron, as the ho^5^C levels were found to be directly proportional to the amount of environmental iron (Kimura et al., 2017). Given the sensitivity of ho^5^C to purification methods, careful re-examination of RNAs in other species could be conducted to determine if this modification is seen in other domains of life.

Though presently unknown how the m^4,4^C modification is generated, it is detected in the human liver cell line Huh7 after exposure to one of the following RNA viruses: Zika virus, Dengue virus, Hepatitis C virus, or poliovirus (McIntyre et al., 2018). It is unclear whether this modification is due to host methyltransferase(s) or if the viral genomes themselves have a factor that could methylate m^4^C or demethylate the 2′-*O*-methyl group in cellular m^4,4^Cm. Of note, arsenite exposure to Huh7 cells caused the appearance of m^4,4^C in total RNA samples, suggesting m^4,4^C formation could occur via a general stress response mechanism in humans (McIntyre et al., 2018). The enzymes RsmH, METTL15, and the DEAD-box RNA helicase DDX6 may also have roles in the formation of m^4,4^C, based on homology searches and siRNA depletion of the DDX6 mRNA having a deleterious effect on m^4,4^C levels (McIntyre et al., 2018). Further analysis may reveal additional properties of m^4,4^C that have gone previously undetected in human cells.

### 6.5 | k^2^C and C^+^ are modified cytidines that use amino acid residues to switch codon recognition in tRNA^Ile^

*Nucleoside modification (IUPAC abbreviation)*: lysidine (k^2^C); agmatidine (C^+^).

*Domain assignments*: A (C^+^), B (k^2^C).

*RNA classes*: ♣.

Codons in mRNA for isoleucine represent a challenge for proper decoding due to the Wobble effect. For codons whose sequence is AUN, three of the possible codons (AUU, AUC, and AUA) encode for isoleucine incorporation into nascent peptide chains while the remaining codon (AUG) encodes for methionine incorporation into nascent peptides (Agris, Vendeix, & Graham, 2007; Crick, 1966; Köhrer et al., 2014). If a tRNA has a guanosine residue in the first position of the anticodon, this guanosine residue can read codons that end in uridine or cytidine (AUU and AUC for isoleucine). However, if the first anticodon residue is uridine, this residue can read codons ending in adenosine or guanosine (AUA for isoleucine and AUG for methionine). Eukaryotes solve this problem of amino acid misincorporation by using either IAU (inosine-adenosine-uridine) or ΨAΨ (pseudouridine-adenosine-pseudouridine) anticodon sequences in tRNAs (Crick, 1966; Senger, Auxilien, Englisch, Cramer, & Fasiolo, 1997).

However, prokaryotes employ a different approach to overcoming amino acid misincorporation. Almost all bacteria utilize k^2^C in place of inosine for the first anticodon sequence (k^2^CAU instead of IAU) (Harada & Nishimura, 1974; Nakanishi et al., 2005; Senger et al., 1997; Soma et al., 2003). However, archaea make use of C^+^ in this location (C^+^AU instead of IAU) (Ikeuchi et al., 2010; Mandal et al., 2010). In the case of k^2^C, a lysine residue is added onto the C34 residues of tRNA^Ile^_2_ at what would be the C2-oxo position by the TilS enzyme. In the case of C^+^, a decarboxylated arginine residue is placed at the same position on a cytidine residue by the TiaS enzyme (Ikeuchi et al., 2010; Köhrer et al., 2014; Mandal et al., 2010). While TilS and TiaS are essential for their host organisms, there are exceptions. These organisms do not have tRNA molecules with a CAU sequence in their anticodon loops; instead, they have UAU sequences (Köhrer et al., 2014). However, a strain of *Bacillus subtilis* was mutated to remove its copy of the *tilS* gene. The resulting viable strain of *B. subtilis* had a low level of misreading AUG codons and possessed a strong binding preference in tRNA^Ile^ for AUA codons (Köhrer et al., 2014). Additional roles that k^2^C and C^+^ may have in biology remain unclear. However, it has been speculated that k^2^C, C^+^, and other RNA–amino acid conjugates may be remnants from the RNA world (Soma et al., 2003) and may have additional aspects of their biology and chemistry that remains unknown. Further, different amino acids may also be conjugated to nucleotides in ways similar to k^2^C and C^+^ that are currently unknown.

## 7 | URIDINE-DERIVED MODIFICATIONS

### 7.1 | cnm^5^U modifications in tRNA^Ile^ avoid mistranslation at the Wobble position

*Nucleoside modification (IUPAC abbreviation)*: 5-cyanomethyluridine (cnm^5^U).

*Domain assignments*: A.

*RNA classes*: ♣.

As discussed in the previous section, many prokaryotes can use a modified cytidine residue in the anticodon loop of tRNA^Ile^ to appropriately translate AUU, AUA, and AUC codons as isoleucine, while AUG codons are translated as methionine (Mandal et al., 2014). However, mistranslation occurred when the first nucleotide in the anticodon loop of tRNA^Ile^ was mutated to a uridine residue (i.e., CAU to UAU) (Mandal et al., 2014). Surprisingly, this mutation allowed for retention of wild-type binding of tRNA^Ile^ to AUA codons to be preserved. However, the mutant tRNA^Ile^ binds to AUU (isoleucine) and AUG (methionine) codons, which poses a problem in translation. Eventually, this mutagenized uridine in UAU was determined to be modified into cnm^5^U through unknown mechanisms (Mandal et al., 2014). While this modification appears to be in haloarchaea, in the euryarchaeote *Methanococcus maripaludis*, and in the euryarchaeote *Methanocaldococcus jannaschii*, it is absent in other archaea, *E. coli*, and *S. cerevisiae* (Mandal et al., 2014; N. Yu, Jora, et al., 2019). How widely distributed this modification is in archaea, which enzyme or enzymes are involved in its synthesis, and its broader roles in biology remain currently unknown.

### 7.2 | m^5^U/rT marks are essential for proper tRNA formation

*Nucleoside modification*: 5-methyluridine (m^5^U) or ribothymidine (rT).

*Domain assignments*: A, B, E.

*RNA classes*: ♣, 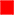, 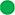, 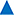.

m^5^U, also referred to as rT, is a common modification in the tRNAs of bacteria and eukaryotes, but limited to the order Thermococcales within Euryarchaeota and the phylum Nanoarchaeota in archaea (Auxilien et al., 2011). In *E. coli* tRNAs, TrmA catalyzes the formation of m^5^U. A mammalian homolog of TrmA is TRMT2A (Chang et al., 2019). m^5^U is normally found in the TΨC-loop of tRNAs at position 54 as part of the G-m^5^U-Ψ-C motif, where m^5^U54 aids in the stabilization of tRNA tertiary structure (Figure 4, Table 2) (Auxilien et al., 2011; Björk & Neidhardt, 1975; Hur, Stroud, & Finer-Moore, 2006; Urbonavicius, Skouloubris, Myllykallio, & Grosjean, 2005). One enzyme that catalyzes the formation of m^5^U54 in tRNA is TrmFO, while another is TrmA (Lartigue et al., 2014; Urbonavicius et al., 2005). Further, a TrmFO homolog in *Mycoplasma capricolum* subsp. *capricolum*, renamed to be RlmFO, was also discovered to confer m^5^U1939 marks in the large ribosomal subunit (Lartigue et al., 2014). However, there are other enzymes that form m^5^U marks in rRNA. In the large ribosomal subunit in *E. coli*, RlmC catalyzes m^5^U747 while RlmD catalyzes m^5^U1939 (Auxilien et al., 2011; Madsen, Mengel-Jørgensen, Kirpekar, & Douthwaite, 2003). These m^5^U methyltransferases are fairly universal in bacteria and eukaryotes, but are unique to Thermococcales and Nanoarchaeota. It is possible that the common ancestor of the two archaeal groups may have received an *rlmD*-like gene of bacterial origin via horizontal gene transfer (Auxilien et al., 2011). m^5^U can also be found on the mitochondrial small rRNA in hamster and human at m^5^U429 and the 23S rRNA large subunit in *E. coli* at m^5^U747 and m^5^U1939 (Baer & Dubin, 1981; D. Dubin, 1974; Madsen et al., 2003; Rebelo-Guiomar, Powell, Van Haute, & Minczuk, 2019). TrmA catalyzes the formation of m^5^U marks on *E. coli* ribosomes (Ranaei-Siadat et al., 2013). However, the roles of m^5^U in structural features of rRNA remain unclear. Also, two recent high-throughput sequencing projects examined m^5^U in RNAs from human cells (Carter et al., 2019; Cheng et al., 2020). While the vast majority of m^5^U marks were detected in tRNAs, several mRNAs and lncRNAs also contained m^5^U marks (Carter et al., 2019; Cheng et al., 2020). Importantly, several RNAs were bound to TRMT2A, but did not contain methylated uridines, suggesting that TRMT2A has additional biological roles that remain currently unknown (Carter et al., 2019). Moreover, in human cells, the methyl group donor for m^5^U in mRNAs is SAM (Cheng et al., 2020). In tmRNA, an m^5^U mark is positioned within the tRNA-like domain at the location that would correspond to the TΨC-loop in tRNA (Ranaei-Siadat et al., 2013).

The protein TRMT2A has also been studied for its effects in cancer. Ectopic expression of TRMT2A has been shown to have an inhibitory effect on cell growth and TRMT2A was subsequently determined to be a cell cycle regulator (Chang et al., 2019). While TRMT2A is found in nuclei of HeLa cells (Chang et al., 2019), the cytosolic localization of TRMT2A in HER2+ breast cancer patients represents a high degree of likelihood that a recurrence of breast cancer would occur (Hicks et al., 2010). Further research into the functionality of m^5^U and whether its overabundance can be useful in cancer diagnostics remains an intriguing avenue of research.

### 7.3 | m^1^Ψ marks are essential for proper tRNA formation in archaea and can be found in rRNA

*Nucleoside modification (IUPAC abbreviation)*: *N*^1^-methylpseudouridine (m^1^Ψ).

*Domain assignments*: A, E.

*RNA classes*: ♣, 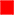.

m^1^Ψ and m^5^U modifications have been found in multiple archaeal tRNAs in the TΨC-loop at position 54 (Gupta, 1984; Pang et al., 1982). m^1^Ψ54 is formed in two steps. First, Ψ54 is generated by Pus10 (Gurha & Gupta, 2008). Subsequently, Ψ54 is methylated by TermY (Chatterjee et al., 2012). Notably, the residue identity at position 55 affects the methylation of Ψ54. The formation of Ψ55 by Pus10 enables the generation of m^1^Ψ54, while a cytidine at nucleotide 55 decreases methylation efficiency (Chatterjee et al., 2012; Gurha & Gupta, 2008). However, a purine at nucleotide 55 abrogates the formation of m^1^Ψ54 from Ψ54 (Chatterjee et al., 2012). m^1^Ψ54/m^5^U54 and the adjacent Ψ55 in tRNAs likely have roles in the formation of a stable tertiary structure of host tRNAs. Ψ55 strengthens a tertiary interaction with the conserved G18 and facilitates intra-loop stacking with a conserved purine at position 57 and with m^1^Ψ54/m^5^U54, which pairs via a reverse-Hoogsteen interaction with the conserved A58 (Chatterjee et al., 2012; Romby et al., 1987).

m^1^Ψ forms via a two-step mechanism in archaeal (16S) and eukaryotic (18S) rRNAs (Brand, Klootwijk, Planta, & Maden, 1978; B. Meyer et al., 2016; Wurm et al., 2010). In the first step, an snR35 H/ACA snoRNP guides the conversion of U54 into Ψ54. Next, the eukaryotic enzyme Nep1/Emg1 and archaeal homologs of Nep1 methylate Ψ54 to form m^1^Ψ54 (B. Meyer et al., 2016; Wurm et al., 2010). In eukaryotes, m^1^Ψ is also a precursor of m^1^acp^3^Ψ (Brand et al., 1978). For example, methylation of Ψ1191 takes place shortly after transcription, whereas the installation of the *N*^3^-acp group via the enzyme Tsr3 possibly occurs before the final cleavage of pre-rRNA into mature 18S rRNA in the cytoplasm (Brand et al., 1978; B. Meyer et al., 2016). Of note, a mutation in the *nep1* gene causes the developmental disorder called Bowen-Conradi syndrome (Armistead et al., 2009; Bowen & Conradi, 1976; B. Meyer et al., 2011). With a homozygous mutation of *nep1* in mice, reduced cell proliferation in brain and other tissues was seen in addition to severe defects in overall growth and in neural tube formation (Armistead et al., 2015). While Bowen–Conradi syndrome is characterized as a ribosomopathy (Armistead et al., 2015), it is unclear how m^1^Ψ marks play roles in the structure of ribosomes or in this syndrome and in other ribosomopathies. Bowen-Conradi syndrome or m^1^Ψ could provide further insights into ribosomopathies in general or perhaps also in additional cellular processes with further research.

### 7.4 | acp^3^U and related marks acp^3^D, acp^3^Ψ, and m^1^acp^3^Ψ are found in either tRNA or rRNA

*Nucleoside modification (IUPAC abbreviation)*: 3(3-amino-3-carboxypropyl) uridine (acp^3^U); 3(3-amino-3-carboxypropyl)-5,6-dihydrouridine (acp^3^D); 3(3-amino-3-carboxypropyl) pseudouridine (acp^3^Ψ); 1-methyl-3(3-amino-3-carboxypropyl) pseudouridine (m^1^acp^3^Ψ).

*Domain assignments*: A (acp^3^U), B (acp^3^U), E.

*RNA classes*: ♣ (acp^3^U, acp^3^D), 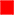 (acp^3^U, acp^3^Ψ, m^1^acp^3^Ψ).

acp^3^U was first detected in *E. coli* tRNA^Phe^ and was found to be in a signature sequence (D[dihydrouridine]-acp^3^U-A) in the D-loop of prokaryotic and eukaryotic tRNAs (Nishimura, Taya, Kuchino, & Oashi, 1974; Stuart et al., 1996). The enzyme in *E. coli* that synthesizes acp^3^U on tRNA was recently determined to be YfiP, which was renamed as TuaA or TapT (B. Meyer et al., 2020; Takakura, Ishiguro, Akichika, Miyauchi, & Suzuki, 2019). The human homologs of TapT, DTWD1 and DTWD2, confer acp^3^U marks at U20 in human tRNA (Takakura et al., 2019). Moreover, the effects of acp^3^U were determined to promote regional conformational shifting in the D-loop (acp^3^U20) and the variable loop (acp^3^U47) of tRNAs containing acp^3^U (Stuart et al., 1996). The presence of this modification in the tRNAs of several additional eukaryotic species, including *D. melanogaster*, wheat germ, lupine seed, rat, cow (B. Meyer et al., 2020; Takakura et al., 2019), and human, suggests that this modification is conserved in eukaryotes (Chheda et al., 1988). Outside of eukaryotes, there has been an acp^3^U modification observed in the rRNA at U966 of the 16S rRNA of *Haloferax volcanii* and it is conferred by the archaeal homolog of the SAM-dependent enzyme Tsr3 (Kowalak etal., 2000; B. Meyer et al., 2020). The archaeal homologs of Tsr3 were found in the archaeal species *Vulcanisaeta distributa* and *Sulfolobus solfataricus*, suggesting that the acp^3^U mark may be widely distributed in archaea (B. Meyer et al., 2020). Of interest to human health, a decreased abundance of acp^3^U was noted in cancer patient urine, similar to the decreased abundance of Q derivatives in cancer cell lines (Chheda et al., 1988; Elliott et al., 1984; Pathak et al., 2008). This decrease of acp^3^U in urine may be used as a biomarker for cancer diagnostics. Further, a double knockout of *DTWD1/2* in HeLa cells exhibited a severe growth phenotype that did not reveal a loss of tRNA stability, suggesting that DTWD1 and DTWD2 have redundant roles in normal cellular growth in human cells (Takakura et al., 2019).

acp^3^D has been found only in *T. brucei* tRNA^Lys(UUU)^, although the synthesis, function, and distribution of acp^3^D remain to be elucidated (Krog et al., 2011). Separately, a chemically related molecule, m^1^acp^3^Ψ, was discovered. At U1191 on helix 31 of eukaryotic 18S rRNA, the parent uridine molecule is converted into Ψ by the snRNA35 H/ACA snoRNP (Samarsky, Balakin, & Fournier, 1995; Sloan et al., 2017). Next, Nep1/Emg1 methylates this pseudouridine into m^1^Ψ (B. Meyer et al., 2011, 2016). Finally, Tsr3 confers the acp group from SAM onto m^1^Ψ, giving rise to m^1^acp^3^Ψ (B. Meyer et al., 2016). If Nep1 is missing or deficient, the Ψ can still have an acp group attached, yielding acp^3^Ψ (B. Meyer et al., 2011). Neither acp^3^Ψ nor m^1^acp^3^Ψ has been detected in tRNA. Given the conservation of Tsr3 in archaea and throughout eukaryotes, it is quite likely that archaea also have some form of acp^3^Ψ or m^1^acp^3^Ψ, though this proposed distribution of acp^3^Ψ or m^1^acp^3^Ψ remains speculative. Several phenotypes emerged with knockouts of *tsr3* in *S. cerevisiae* and knockdowns of *tsr3* in HCT116 human cells. In *S. cerevisiae*, the *Δtsr3* mutant resulted in hypersensitivity to the protein synthesis inhibitor paromomycin (B. Meyer et al., 2016). The depletion of Tsr3 in human cells caused a buildup of 20S pre-rRNA, suggesting that Tsr3 is crucial in 18S rRNA processing (B. Meyer et al., 2016). Further, as mentioned above, specific *tsr3* mutations result in the formation of Bowen-Conradi syndrome (Wurm et al., 2010). However, what additional functions acp-derived nucleotides may have in overall biology remain unclear.

### 7.5 | m^3^U and m^3^Ψ marks may affect rRNA and tRNA functionality

*Nucleoside modification (IUPAC abbreviation)*: 3-methyluridine (m^3^U); 3-methylpseudouridine (m^3^Ψ).

*Domain assignments*: A (m^3^U), B, E (m^3^U).

*RNA classes*: ♣ (m^3^U), 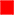.

While m^3^U and m^3^Ψ are isomers of each other, their functionalities in RNAs are strikingly different. In *T. brucei* tRNA^Thr^, m^3^C is deaminated into m^3^U by Trm140 and ADAT2/3 during C-to-U editing (Rubio et al., 2017). The association of Trm140 to ADAT2/3 prevents massive genome- and transcriptome-wide deamination by the ADAT2/3 enzyme (Rubio et al., 2017). Both m^3^U and m^3^Ψ have been identified in the rRNA of bacteria. However, only m^3^U has been found in archaeal and eukaryotic rRNAs. First discovered in *S. cerevisiae* cells (Hall, 1963), m^3^U modifications have since been found in 25S rRNA of *S. cerevisiae*, 23S rRNA of *S. solfataricus*, and 16S rRNA of *E. coli*, among others (J. D. Johnson & Horowitz, 1971; Noon et al., 1998). Though originally found in 18S rRNA of HeLa cells, later studies did not find evidence for the existence of m^3^U there (Iwanami & Brown, 1968; Klagsbrun, 1973). Several enzymes that catalyze this m^3^U modification in rRNAs have been identified. In *S. cerevisiae*, U2634 and U2843 of 28S rRNA are methylated by the SAM-dependent methyltransferases BMT5 and BMT6, respectively (Sharma et al., 2014). In *E. coli*, the only known m^3^U site (U1498 in 16S rRNA) is methylated by the site-specific SAM-dependent methyltransferase RsmE (Basturea, Rudd, & Deutscher, 2006). Interestingly, the human obesity-linked FTO protein is capable of demethylating m^3^U sites in vitro (Jia et al., 2008).

The sole m^3^Ψ mark discovered in *E. coli* occurs at the highly conserved Ψ1915 position in hairpin 69 of the 23S rRNA (Kowalak et al., 1996; Ofengand, Del Campo, et al., 2001; Ofengand, Malhotra, et al., 2001). To make the m^3^Ψ mark, the uridine is first pseudouridylated by the RluD enzyme before being methylated by the SAM-dependent methyltransferase RlmH, also known as YbeA (Ero, Peil, Liiv, & Remme, 2008; Purta, Kaminska, Kasprzak, Bujnicki, & Douthwaite, 2008; Raychaudhuri, Conrad, Hall, & Ofengand, 1998). This methylation of Ψ1915 stabilizes hairpin 69 relative to the pseudouridine alone (Chui, Desaulniers, Scaringe, & Chow, 2002). The m^3^Ψ1915 mark in 23S rRNA is conserved in other bacteria, including the 23S analogs in *D. radiodurans*, *B. subtilis*, and the chloroplast of *Zea mays* (Del Campo et al., 2005). Pseudouridylation, and possibly methylation, of this site may be essential for proper *E. coli* growth and development since knockouts of *rluD* showed growth rates reduced by ~50% when compared with wild-type *E. coli*. However, knockouts of *rlmH* also showed slightly slower growth rates when compared with wild-type *E. coli* (Purta et al., 2008; Raychaudhuri et al., 1998). m^3^Ψ1915 may have a further important role in large ribosomal subunit structure and function. The loop of hairpin 69, which contains m^3^Ψ1915 in *E. coli*, interacts with anticodon arm of A-site tRNA (Yusupov et al., 2001). Additionally, m^3^Ψ1915 has been shown to directly contact residues of the RRF protein, which disassembles the post-translational termination complex of mRNA, tRNA, and rRNA (Agrawal et al., 2004). Additional biological roles of m^3^U and m^3^Ψ remain unclear, though further discovery of these marks in previously unknown RNAs may elucidate novel functions. Further, the role of m^3^U in C-to-U editing may also be more prevalent in eukaryotic organisms or in bacteria and archaea, which may elicit unexpected functions of m^3^U.

### 7.6 | ho^5^U and its derivatives mo^5^U, cmo^5^U, and mcmo^5^U affect base-pairing specificity in tRNAs that contain these modifications

*Nucleoside modification (IUPAC abbreviation)*: 5-hydroxyuridine (ho^5^U); 5-methoxyuridine (mo^5^U); uridine 5-oxyacetic acid (cmo^5^U); uridine 5-oxyacetic acid methyl ester (mcmo^5^U).

*Domain assignments*: B, E (ho^5^U).

*RNA classes*: ♣.

Under nonenzymatic processes, ho^5^U is commonly found to spontaneously occur due to oxidative damage to cytosines in DNA or RNA. However, ho^5^U can be enzymatically synthesized at U34 of tRNAs via the TrhOP enzyme complex (Sakai, Kimura, & Suzuki, 2019). Homologs of TrhO were found in bacterial and eukaryotic organisms, while TrhP was exclusive to bacterial species (Sakai et al., 2019). Regardless of origin, ho^5^U remains capable of base pairing with guanosine by adopting a normal Watson–Crick base pair arrangement instead of a Wobble base pair arrangement (Sakai et al., 2019). Knocking out the TrhOP enzymes introduces a temperature-sensitive phenotype in *E. coli* and decreases the efficiency of decoding codons ending in G, specifically the GCG and UCG codons (Sakai et al., 2019).

ho^5^U is often utilized as an intermediate for mo^5^U, cmo^5^U, and mcmo^5^U in bacteria (Sakai et al., 2019). The enzyme CmoB carboxymethylates ho^5^U34 in tRNA into cmo^5^U34 using a highly unusual SCM-SAH molecule, which is generated when the enzyme CmoA uses a carboxyl group from prephenate to carboxylate SAH (J. Kim et al., 2013). The cmo^5^U34 modification can be further methylated by the SAM-dependent enzyme CmoM into mcmo^5^U (Sakai, Miyauchi, Kimura, & Suzuki, 2016). The mcmo^5^U mark in one tRNA, tRNA^Pro3^, is dynamic, as its abundance is positively correlated with the number of *E. coli* and perhaps in other organisms (Sakai et al., 2016). The presence of cmo^5^U and mcmo^5^U appears to be exclusive to bacteria, possibly due to the requirement of prephenate, one of the intermediates in the chorismate–shikimate pathway (Sakai et al., 2016, 2019). In a prior observation, ho^5^U can also slow the rate of RNA and protein synthesis in bacteria, viruses, and tumor cells if introduced in media as a mononucleotide (D. A. Smith & Visser, 1965). Additional research is underway for finding previously unknown ways in which ho^5^U participates in cellular processes, especially with its role in oxidative damage and whether ho^5^U and its derivatives have any roles in RNA structure (Sakai et al., 2019). Perhaps these ho^5^U-related processes could become a new drug target or be useful diagnostics for diseases related to oxidative damage.

### 7.7 | cmnm^5^U, cm^5^U, and their derivatives decorate tRNA molecules

*Nucleoside modification (IUPAC abbreviation)*: 5-carboxymethylaminomethyluridine (cmnm^5^U); 5-methylaminomethyluridine (mnm^5^U); 5-aminomethyluridine (nm^5^U); 5-carboxymethyluridine (cm^5^U); 5-methoxycarbanylmethyluridine (mcm^5^U); 5-carboxyhydroxymethyluridine (chm^5^U); 5-carbamoylmethyluridine (ncm^5^U)5-carboxyhydroxymethyluridine methyl ester (mchm^5^U); 5-carbamoylhydroxymethyluridine (nchm^5^U).

*Domain assignments*: A (not cmnm^5^U, mnm^5^U and nm^5^U), B (cmnm^5^U, mnm^5^U, and nm^5^U), E (not mnm^5^U, nm^5^U, and nchm^5^U).

*RNA classes*: ♣.

**FIGURE 8:**
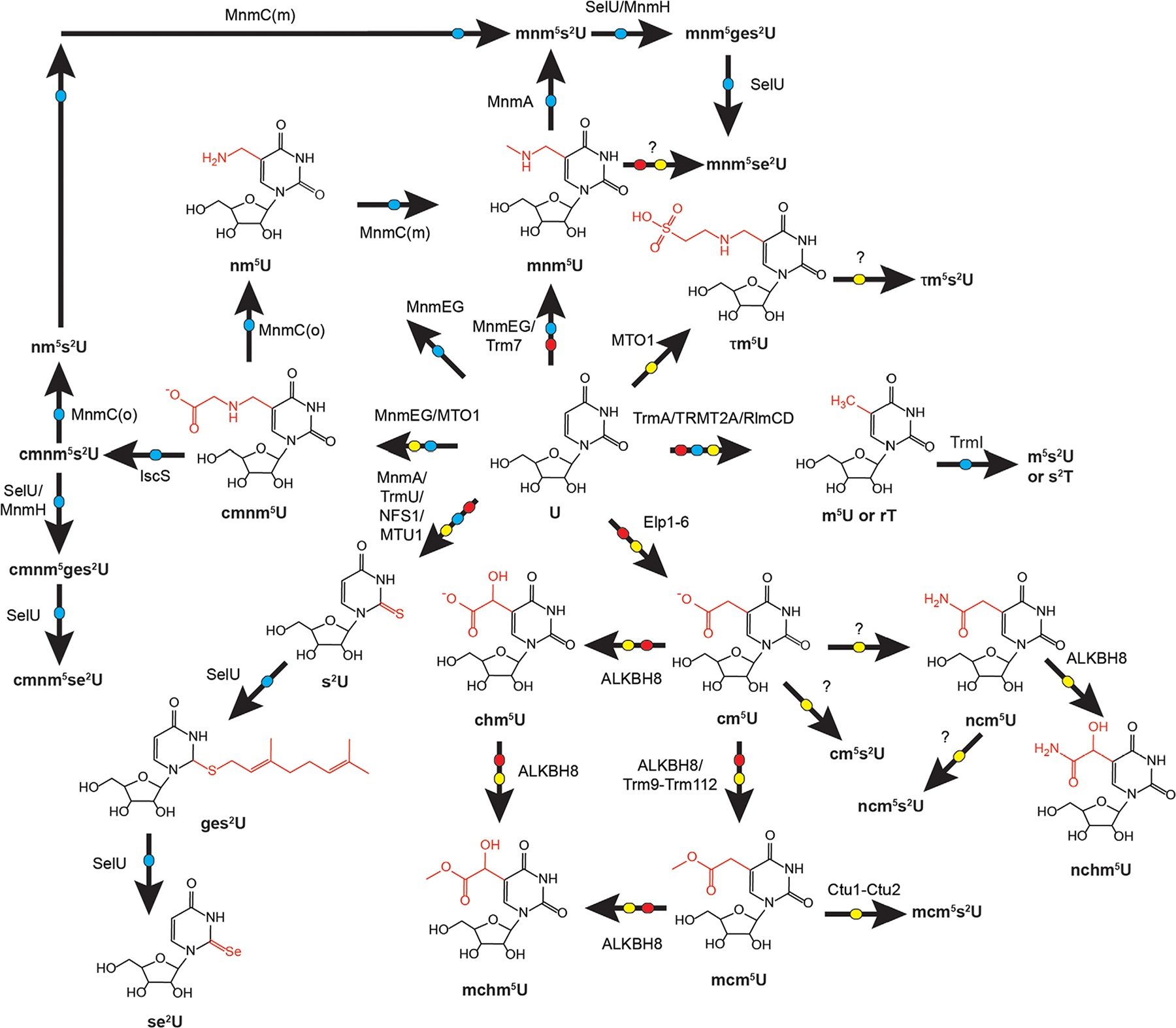
Scheme for cmnm^5^U, cm^5^U, τm^5^U, s^2^U, se^2^U, and related derivatives. Shown in red are chemical modifications that occur in the production of 5-methyluridine or thiolated uridine derivatives: m^5^U or rT, cmnm^5^U, mnm^5^U, nm^5^U, cm^5^U, mcm^5^U, chm^5^U, mchm^5^U, nchm^5^U, ncm^5^U, τm^5^U, s^2^U, m^5^s^2^U or s^2^T, cmnm^5^s^2^U, mnm^5^s^2^U, nm^5^s^2^U, τm^5^s^2^U, mcm^5^s^2^U, ncm^5^s^2^U, cm^5^s^2^U, se^2^U, ges^2^U, cmnm^5^ges^2^U, mnm^5^ges^2^U, cmnm^5^se^2^U, and mnm^5^se^2^U. Modifications containing a 2-thio group, a 2-geranyl group, or a 2-seleno group are listed with only abbreviations, while the structures for s^2^U, ges^2^U, and se^2^U are shown as exemplars for each of these modification types. Enzymes that catalyze each reaction are adjacent to arrow, otherwise a question mark (?) indicates enzyme is unknown. Arrow tails containing red, blue, or yellow ellipses denote reactions that occur in archaea, bacteria, or eukaryotes, respectively

Uridines in the anticodon loop (positions 34–37) of select tRNA molecules can be modified with methyl (m) or carboxymethyl (cm) derivatives (Figure 8) (C. Chen, Huang, Anderson, & Byström, 2011). U5 in bacterial tRNA is modified by the bacterial enzymatic complex MnmE and MnmG/GidA to contain either a carboxymethylaminomethyl (cmnm) or a aminomethyl (nm) group resulting in the formation of either cmnm^5^U or nm^5^U, respectively (Ayadi, Galvanin, Pichot, Marchand, & Motorin, 2019; Mihara et al., 2002; Moukadiri et al., 2009; Moukadiri, Villarroya, Benítez-Páez, & Armengod, 2018; Sierant et al., 2018). The formation of cmnm^5^U makes use of a 5-formyluridine intermediate (J. Kim & Almo, 2013). An additional interconversion can occur from cmnm^5^U into mnm^5^U. Here, the carboxymethyl group is cleaved off cmnm^5^U via the oxidoreductase domain of the MnmC(o) enzyme, yielding nm^5^U (J. Kim & Almo, 2013; Moukadiri et al., 2018). nm^5^U, either newly formed or originating from cmnm^5^U, can then be methylated by the methyltransferase domain of the MnmC(m) enzyme to yield mnm^5^U (J. Kim & Almo, 2013; Moukadiri et al., 2018). In an unrelated reaction mechanism in *S. cerevisiae*, MTO1 adds a glycine residue to U34, resulting in the formation of cmnm^5^U (Fakruddin et al., 2018; Umeda et al., 2005). However, it is unknown if cmnm^5^U can also interconvert into mnm^5^U or nm^5^U in eukaryotes using enzymatic processes similar to bacteria.

The Elongator complex (Elp1–6), which is highly conserved in eukaryotes and supports mRNA transcription elongation by RNA Pol II, carboxymethylates uridine residues and forms cm^5^U (Figure 8) (Karlsborn et al., 2014). Archaeal homologs of the third subunit of the Elongator complex, Elp3, have also been shown to carboxymethylate uridine residues in tRNAs (Selvadurai, Wang, Seimetz, & Huang, 2014). Once cm^5^U forms, it can undergo one of several synthetic pathways. cm^5^U can be methylated with a methyl group from SAM by the enzyme ALKBH8 to form mcm^5^U (Songe-Møller et al., 2010). In archaea and in eukaryotes, methylation of cm^5^U to mcm^5^U is catalyzed by the Trm9–Trm112 complex (van Tran et al., 2018). cm^5^U can also be hydroxylated by the ALKBH8 enzyme to form chm^5^U (van den Born et al., 2011). Both chm^5^U and mcm^5^U can be independently methylated or carboxyhydroxylated, respectively, by ALKBH8 to form mchm^5^U (Karlsborn et al., 2014; van den Born et al., 2011). Finally, cm^5^U can be modified by an unknown enzyme to contain a carbamoylmethyl group to form ncm^5^U and hydroxylated to form nchm^5^U with the eukaryotic ALKBH8 enzyme (C. Chen et al., 2011; van den Born et al., 2011). Altogether, these modifications have roles in proper decoding of codons and specificity of tRNA-binding events to mRNA (C. Chen et al., 2011). However, many of the implications of these modifications in biology remain unknown.

### 7.8 | τm^5^U is important to mitochondrial tRNAs

*Nucleoside modification (IUPAC abbreviation)*: 5-taurinomethyluridine (τm^5^U).

*Domain assignments*: E.

*RNA classes*: ♣.

τm^5^U was discovered in mammalian tRNA^Lys^ (Fakruddin et al., 2018). The enzyme MTO1 adds the taurine modification in mitochondrial tRNAs and can be found in position U34 of the anticodon loop within tRNA^Lys^ (Figures 4 and 8) (Fakruddin et al., 2018). An siRNA knockdown of a mitochondrial homolog of MTO1 resulted in the absence of τm^5^U and reduced translational activity of UUG codon (Umeda et al., 2005). The lack of τm^5^U results in a deficiency of mitochondrial complex I that is seen in patients with MELAS and MERRF (Umeda et al., 2005). MELAS and MERRF result from mitochondrial distress, as indicated by the severely impaired translation and respiration events within the mitochondria (Fakruddin et al., 2018). Additionally, trafficking of mitochondrial proteins encoded by nuclear DNA was negatively affected by the resulting abnormal mitochondrial shape (Fakruddin et al., 2018). Inner membrane proteins of the mitochondria, such as Opa1, are unable to localize to the inner membrane; therefore, they aggregate in the cytosol, inducing a cytotoxic unfolded protein response (Fakruddin et al., 2018). This response has been studied as a drug target, where a taurine-conjugated bile acid molecule was discovered to possibly supply the missing taurine in mouse livers and could alleviate this unfolded protein response (Fakruddin et al., 2018). However, as mitochondrial proteins are still mistranslated, further research is necessary to elucidate a cure for these diseases (Fakruddin et al., 2018).

### 7.9 | Sulfur-containing modifications, such as s^2^U and its derivatives and s^4^U, are essential for tRNA structural stability as well as translational fidelity and efficiency

*Nucleoside modification (IUPAC abbreviation)*: 2-thiouridine (s^2^U); 5-methyl-2-thiouridine (m^5^s^2^U); 5-carboxymethylaminomethyl-2-thiouridine (cmnm^5^s^2^U); 5-methylaminomethyl-2-thiouridine (mnm^5^s^2^U); 5-aminomethyl-2-thiouridine (nm^5^s^2^U); 5-taurinomethyl-2-thiouridine (τm^5^s^2^U); 5-methoxycarbonylmethyl-2-thiouridine (mcm^5^s^2^U); 5-carbamoylmethyl-2-thiouridine (ncm^5^s^2^U); 5-carboxymethyl-2-thiouridine (cm^5^s^2^U); 5-cyanomethyl-2-thiouridine (cnm^5^s^2^U); 4-thiouridine (s^4^U).

*Domain assignments*: A (s^2^U, m^5^s^2^U, cm^5^s^2^U, cnm^5^s^2^U, s^4^U), B (not cm^5^s^2^U, mcm^5^s^2^U, ncm^5^s^2^U, τm^5^s^2^U, cnm^5^s^2^U), E (not m^5^s^2^U, mnm^5^s^2^U, nm^5^s^2^U, cmnm^5^s^2^U, cnm^5^s^2^U, s^4^U).

*RNA classes*: ♣.

The formation of s^2^U involves an array of enzymatic processes, ultimately resulting in a modification that is found in nine other uridine modifications (Figure 8, Table 1). The modified nucleoside derivatives featuring an *x*s^2^U (where *x* denotes one of several possible modifications listed above) are covered more in-depth in sections below. However, the common feature for all these modifications is the substitution of a sulfur at O2 of uracil. To add this sulfur, a cysteine is dethiolated by either IscS or YrvO in *E. coli* or *B. subtilis*, respectively (Black & Dos Santos, 2015; Lauhon, 2002; Mihara et al., 2002; Shigi, 2014), or by homologs of NFS1/Ncs6/Ctu1 in archaea and eukaryotes (Y. Liu, Long, Wang, Söll, & Whitman, 2014). *E. coli* continues shuttling the sulfur molecule through TusABCDE proteins before transferring the sulfur to the MnmA protein, which thiolates the uridine at nt 34 in tRNA (Black & Dos Santos, 2015). *B. subtilis* does not have the TusABCDE operon; therefore, YrvO directly passes the sulfur molecule to MnmA, which then thiolates tRNAs (Black & Dos Santos, 2015). In archaea and possibly eukaryotes, NFS1/Ncs6/Ctu1 first adenylates U34 in select tRNAs prior to thiolation from NFS1/Ncs6/Ctu1 (Y. Liu et al., 2014). While it is not known precisely which type of sulfur modification is directly replaced on uridine residues in eukaryotic tRNAs, mitochondrial NFS1, Isu1/2, and Atm1 in *S. cerevisiae* and Tum1, a persulfide mediator and activator of NFS1, have roles in forming and transporting s^2^U marks (Nakai et al., 2004; Noma, Sakaguchi, & Suzuki, 2009; Pandey et al., 2018). Moreover, the MTU1 enzyme is a mitochondrial-specific 2-thiouridylase that is a homolog of the MnmA enzyme (Black & Dos Santos, 2015; Noma et al., 2009). Humans use a homolog of MTU1, also known as TrmU, to thiolate uridine residues in mitochondrial tRNAs (Black & Dos Santos, 2015; Umeda et al., 2005).

The functions of the 2-thio group in numerous uridine nucleotides depend on where the modified ribonucleotide occurs within the tertiary structure of tRNA. For example, s^2^U in the acceptor stems (TΨC-loop and D-loop) is important for ensuring tRNA structural stability and recognition by specific enzymes (reviewed in C. Zheng, Black, & Dos Santos, 2017). Sulfur-containing modifications within the anticodon stem-loop (nts 32, 33, 34, 37) are usually important for translation. s^2^U33 can have roles in negatively regulating C34-to-U34 editing and in subsequent translation (reviewed in Čavužić & Liu, 2017; Z. Paris, Fleming, & Alfonzo, 2012). The s^2^U33 mark has been found in *T. brucei* and *Leishmania spp*. mitochondrial tRNA^Trp-CCA^ (Crain et al., 2002; Z. Paris et al., 2012). s^2^U and its derivatives that are present at nt 34 within tRNA^Glu^, tRNA^Gln^, and tRNA^Lys^ ensure specificity or flexibility in the discrimination of multiple codons (reviewed in Čavužić & Liu, 2017; Lim & Curran, 2001; C. Zheng, Black, et al., 2017). Early studies indicated that the presence of s^2^U34 maintains a Watson–Crick base pair with A and restricts Wobble pairing with guanosine, though s^2^U34 can still pair with both A and G. However, additional modifications to position C5 of s^2^U34 can affect the preference for U–A pairing and favored U–G interactions (Agris et al., 1992; Egert, Lindner, Hillen, & Boehm, 1980; Krüger & Sørensen, 1998). The 2-thio group of m^5^s^2^U34 and its derivatives enhances translation specificity, contributing to the recognition of tRNAs by aminoacyl-tRNA synthetases (Madore et al., 1999; Rodriguez-Hernandez et al., 2013; Sylvers, Rogers, Shimizu, Ohtsuka, & Söll, 1993; Tamura, Himeno, Asahara, Hasegawa, & Shimizu, 1992). These modifications ensure preferential base pairing with purines in the codons of mRNAs and prevent misreading caused by interactions with pyrimidines (reviewed in Shigi, 2014; C. Zheng, Black, et al., 2017). s^2^U and its derivatives are involved in codon recognition within the ribosome and prevent frameshifting during translation (Rodriguez-Hernandez et al., 2013; Shigi, 2014; C. Zheng, Black, et al., 2017). For example, m^5^s^2^U (also known as s^2^T) at nt 54 plays an important role in protein expression in thermophilic organisms such as the bacteria *T. thermophilus* or the archaea *P. furiosus* (Kowalak, Dalluge, McCloskey, & Stetter, 1994; Shigi, 2014; Shigi et al., 2006; Shigi, Sakaguchi, Asai, Suzuki, & Watanabe, 2008; Yokoyama et al., 1987). The rigid structure of this modification increases tRNA thermostability through stabilization of the D- and TΨC-loops (reviewed in Shigi, 2014).

Thiolation events can be found in other modified uridine residues on tRNAs. cmnm^5^U and mnm^5^U can be thiolated at C2 via the bacterial enzymes IscS and MnmA to form cmnm^5^s^2^U or mnm^5^s^2^U (Mihara et al., 2002; Sierant et al., 2018). cmnm^5^s^2^U can also be converted into mnm^5^s^2^U or nm^5^s^2^U via the bacterial MnmC enzyme (Bujnicki et al., 2004). Though it is unclear which thiolase forms cm^5^s^2^U from cm^5^U, mcm^5^U can be thiolated to form mcm^5^s^2^U using the eukaryotic Ctu1–Ctu2 enzymatic complex (Dewez et al., 2008; Lentini, Ramos, & Fu, 2018). Also, once ncm^5^U is formed, it can be thiolated through an unknown thiolase to form ncm^5^s^2^U (C. Chen et al., 2011). Through an unknown mechanism, τm^5^U can be thiolated in human mitochondrial tRNA to form τm^5^s^2^U (Fakruddin et al., 2018). τm^5^s^2^U-deficient human mitochondrial tRNAs were also found in patients who had MERRF and cannot translate the codons AAA or AAG (Umeda et al., 2005). Through an unknown mechanism, cnm^5^U can be thiolated in tRNA^Leu^ from euryarchaeote *M. jannaschii* to form cnm^5^s^2^U (N. Yu, Jora, et al., 2019). While cnm^5^s^2^U has been found only in *M. jannaschii* to date, *M. jannaschii* and *Haloferax volcanii* represent the only two archaeal species to have had their tRNAs analyzed for the presence of modified ribonucleosides. This dearth of knowledge of archaeal tRNA modifications and their distributions within archaea may lead to further discoveries of previously unknown modified ribonucleosides. Moreover, given the large number of s^2^U derivatives, it would be interesting to see if there are additional thiolated cytidine derivatives beyond s^2^C and ms^2^C.

Like s^2^U modifying enzymes, there are enzymes specific to the Gram-negative and Gram-positive bacteria synthetic reactions of s^4^U. In the Gram-negatives *E. coli* and *S. enterica*, IscS removes a sulfur atom from cysteine while ThiI thiolates U8 in tRNA (Rajakovich, Tomlinson, & Dos Santos, 2012). However, in Gram-positive *B. subtilis* and the other Gram-positives in Firmicutes, NifZ instead removes the sulfur from cysteine and directly thiolates the uridine residue in tRNA with a ThiI homolog (Rajakovich et al., 2012). The archaeal homologs of ThiI also incorporate s^4^U in tRNA. Eukaryotes do not appear to have s^4^U, though homologs of ThiI have been found in some eukaryotic genomes (reviewed in Čavužić & Liu, 2017; Kotera et al., 2010). s^4^U can be located at nt 8 in tRNA, which is at the intersection of the tRNA acceptor- and D-arms (Figure 4, Table 2). Upon exposure to near-UV light, s^4^U crosslinks to C13 in tRNA (reviewed in Palenchar, Buck, Cheng, Larson, & Mueller, 2000). The s^4^U-C13 crosslink prevents proper tRNA aminoacylation, leading to uncharged tRNA accumulation and translational pausing (Čavužić & Liu, 2017; Favre, Yaniv, & Michels, 1969). These effects then activate the SOS response, which mitigates damage to DNA due to UV-light exposure (Björk & Hagervall, 2014; Čavužić & Liu, 2017; Thomas & Favre, 1980). The thiolation of s^4^U8 of tRNAs has also been shown to positively maintain tRNA homeostasis in *Vibrio cholerae* (Kimura & Waldor, 2019). In *thiI* deletion strains of *V. cholerae*, several tRNAs were decreased, as it was determined that the lack of s^4^U8 designates these hypomodified tRNAs for degradation by the RNA degradosome (Kimura & Waldor, 2019). While s^4^U is used in PAR-CLIP at high concentrations that are in excess of 100 μM, this high concentration has been shown to interfere with proper 47S rRNA processing in U2OS and 2fTGH human cell cultures; therefore, caution is warranted when using s^4^U in PAR-CLIP or other related experiments (Burger et al., 2013). Further roles of sulfur-containing ribonucleotides and the enzymes involved may have further unknown roles in structural formation and biological relevance to thermophilic organisms.

### 7.10 | Selenium-modified uridines and their precursors are thought to aid in translational accuracy and efficiency

*Nucleoside modification (IUPAC abbreviation)*: 2-selenouridine (se^2^U); 2-geranylthiouridine (ges^2^U); 5-carboxymethylaminomethyl-2-geranylthiouridine (cmnm^5^ges^2^U); 5-methylaminomethyl-2-geranylthiouridine (mnm^5^ges^2^U); 5-carboxymethylaminomethyl-2-selenouridine (cmnm^5^se^2^U); 5-methylaminomethyl-2-selenouridine (mnm^5^se^2^U).

*Domain assignments*: A (se^2^U and mnm^5^se^2^U), B, E (mnm^5^se^2^U).

*RNA classes*: ♣.

While there are several sulfur-modified uridine residues currently known, selenium-containing ribonucleosides can also be found in tRNAs, specifically in the anticodon loop at position 34 (Figures 4 and 8, Table 1). In bacteria, converting s^2^U to se^2^U is a two-step process catalyzed by SelU (Sierant et al., 2018). In the first step, the sulfur of s^2^U is geranylated to yield ges^2^U. By itself, ges^2^U is a stable modification that is thought to shift codon bias and frameshifting rates during translation, which is possibly due to a preference of ges^2^U to base pair with guanosine and not adenosine (Sierant et al., 2016). Once ges^2^U is formed on tRNA, the second step results in a selenation by replacing the geranylsulfate with a selenide group, thus forming se^2^U (Sierant et al., 2016). In archaea, a YbbB-like enzyme forms se^2^U, though it is unknown if this requires a geranylated intermediate (D. Su, Ojo, Söll, & Hohn, 2012). Separately, once cmnm^5^s^2^U and mnm^5^s^2^U are thiolated in bacteria, these modified uridines can be geranylated by SelU or by MnmH, yielding cmnm^5^ges^2^U or mnm^5^ges^2^U (Jäger, Chen, & Björk, 2016; Sierant et al., 2016, 2018). These modifications can be subsequently selenated by the SelU enzyme to yield cmnm^5^se^2^U or mnm^5^se^2^U (Dumelin, Chen, Leconte, Chen, & Liu, 2012; Sierant et al., 2018). mnm^5^se^2^U is the most abundant selenonucleoside, found in all three domains of life, and commonly occurs in the Wobble position of the anticodon of three tRNA acceptors (tRNA^Glu^, tRNA^Gln^, and tRNA^Lys^) (D. Su et al., 2012). Se2 of mnm^5^se^2^U residues discriminates against the formation of G–U Wobble pairs. Instead, the selenium promotes the formation of A–U base pairs in RNA in vitro, suggesting selenium modifications may be important in translational fidelity (Huiyan Sun et al., 2012). While it is presently unknown if the bacterial selenation process is present in archaea or eukaryotes, the presence of mnm^5^se^2^U in archaea and eukaryotes suggests that a similar process may be possibly occurring.

Further, GTPBP3 and the taurine-adding enzyme MTO1 have also been linked to selenation reactions in mitochondrial tRNAs (Bykhovskaya, Mengesha, et al., 2004; Kopajtich et al., 2014; Xinjian Wang, Yan, & Guan, 2010; Yim, Moukadiri, Björk, & Armengod, 2006). Deficiencies in mitochondrial GTPBP3 have been linked to hypertrophic cardiomyopathy, lactic acidosis, encephalopathy, and deafness through deficiencies in cmnm-containing uridines (Bykhovskaya, Mengesha, et al., 2004; Kopajtich et al., 2014). While MTO1 deficiency can also be linked to these diseases, MTO1 deficiency is typically linked to deficiencies in taurine-containing uridine modifications (Fakruddin et al., 2018; Jonkhout et al., 2017; X. Wang et al., 2010). Additional discoveries of geranyl derivatives and selenium-containing RNAs will undoubtedly continue to provide new insights into biology and in further elucidating the differences between sulfur- and selenium-containing RNAs.

## 8 | MODIFICATIONS TO THE RIBOSE MOIETY

### 8.1 | Ribosyl-RNA modifications are found in select RNAs

*Nucleoside modification (IUPAC abbreviation)*: 2′-*O*-ribosyladenosine (Ar(p)); 2′-*O*-ribosylguanosine (Gr(p)).

*Domain assignments*: E.

*RNA classes*: ♣.

Ar(p) and Gr(p) represent disaccharide-modified ribonucleosides, whereby the 2′-hydroxyl of either adenosine or guanosine forms an α-(1,2)-glycosidic linkage with an additional 5′-phosphorylated ribose molecule. Though first discovered at position 64 in eukaryotic tRNA^Met^ that initiates translation, these disaccharide-modified ribonucleosides have been found in poly(ADP-ribose) (PAR), in select enzymes as prosthetic groups, and in assorted naturally derived compounds or secondary metabolites (Desgrès, Keith, Kuo, & Gehrke, 1989; Efimtseva, Kulikova, & Mikhaĭlov, 2009; Glasser, Desgres, Heitzler, Gehrke, & Keith, 1991; Juarez-Salinas, Mendoza-Alvarez, Levi, Jacobson, & Jacobson, 1983; Keith, Glasser, Desgrés, Kuo, & Gehrke, 1990; Q. Liu, van der Marel, & Filippov, 2019; Simsek & RajBhandary, 1972). Ar(p) and Gr(p) are synthesized by the enzyme Rit1 through addition of a PRPP group directly onto A64 or G64 in tRNA^Met^, which potentially distorts the RNA phosphodiester backbone of the initiator tRNA^Met^ and prevents the initiator tRNA^Met^ from binding to eEF1A (Åström & Byström, 1994; Kiesewetter, Ott, & Sprinzl, 1990; Kolitz & Lorsch, 2010). PAR is synthesized from NAD^+^ via eukaryotic PAR polymerases (Berger, Ramírez-Hernández, & Ziegler, 2004; Pleschke, Kleczkowska, Strohm, & Althaus, 2000; Rongvaux, Andris, Van Gool, & Leo, 2003; Schreiber, Dantzer, Ame, & de Murcia, 2006). One of the primary functions of PAR is aiding in the DNA-strand repair process, as it is added to DNA-strand breaks and serves as a signaling molecule to initiate DNA repair processes (Schreiber et al., 2006). Moreover, PAR can have roles in acute and chronic inflammation, while also contributing to the progression of degenerative diseases (Schreiber et al., 2006). Further discoveries pertaining to PAR, Ar(p), and Gr(p) will perhaps expand the roles of these modified nucleosides throughout eukaryotic biology.

### 8.2 | 2′-O-Methyl RNA modifications exist in otherwise unmodified and additionally modified ribonucleosides

*Nucleoside modification (IUPAC abbreviation)*: 2′-*O*-methyladenosine (Am); *N*^6^,2′-*O*-dimethyladenosine (m^6^Am); *N*^6^,*N*^6^,2′-*O*-trimethyladenosine (m^6,6^Am); *N*^1^,2′-*O*-dimethyladenosine (m^1^Am); 2′-*O*-methylinosine (Im); 1,2′-*O*-dimethylinosine (m^1^Im); 2′-*O*-methylguanosine (Gm); 1,2′-*O*-dimethylguanosine (m^1^Gm); *N*^2^,2′-*O*-dimethylguanosine (m^2^Gm); *N*^2^,*N*^2^,2′-*O*-trimethylguanosine (m^2,2^Gm); *N*^2^,2′-*O*-7-trimethylguanosine (m^2,7^Gm); 2′-*O*-methylcytidine (Cm); 4,2′-*O*-dimethylcytidine (m^4^Cm); 5,2′-*O*-dimethylcytidine (m^5^Cm); 5-formyl-2′-*O*-methylcytidine (f^5^Cm); *N*^4^,*N*^4^,2′-*O*-trimethylcytidine (m^4,4^Cm); *N*^4^-acetyl-2′-*O*-methylcytidine (ac^4^Cm); 2′-*O*-methyl-5-hydroxymethylcytidine (hm^5^Cm); 2′-*O*-methyluridine (Um); 3,2′-*O*-dimethyluridine (m^3^Um); 5,2′-*O*-dimethyluridine (m^5^Um); 2′-*O*-methylpseudouridine (Ψm); 2-thio-2′-*O*-methyluridine (s^2^Um); 5-methoxycarbonylmethyl-2′-*O*-methyluridine (mcm^5^Um); 5-carboxymethylaminomethyl-2′-*O*-methyluridine (cmnm^5^Um); 5-carbamoylmethyl-2′-*O*-methyluridine (ncm^5^Um); 5-(isopentenylaminomethyl)-2′-*O*-methyluridine (inm^5^Um); 5-(carboxyhydroxymethyl)-2′-*O*-methyluridine methyl ester (mchm^5^Um); 2′-*O*-methyluridine 5-oxyacetic acid methyl ester (mcmo^5^Um).

*Domain assignments*: A, B, E; see the following reviews for prokaryotic assignments (Ayadi et al., 2019; Boccaletto et al., 2018) and RMBase for eukaryotic assignments (Xuan et al., 2018).

*RNA classes*: ♣, 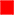, 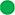, 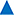; see for RNA classes (Ayadi et al., 2019; Boccaletto et al., 2018; Xuan et al., 2018).

While there are at least 28 2′-*O*-methyl modifications (Nm) to ribonucleosides, their origins are thought to be quite similar (Table 1). Assorted archaeal and eukaryotic snoRNAs, many of which are Box C/D snoRNAs, aid in assembling the snoRNPs NOP56, NOP58, fibrillarin, and 15.5K/Snu13/L7Ae. This protein complex then coordinates 2′-*O*-methylation events on pre-rRNA transcripts and other RNAs (reviewed in Sloan et al., 2017; Yip, Shigematsu, Taylor, & Baserga, 2016; Zhu, Pirnie, & Carmichael, 2017). Moreover, snoRNAs are also used by archaeal species to conduct 2′-*O*-methylation events on tRNA. Separately, bacteria also conduct 2′-*O*-methylations on ribosomes, although these methylations are made by site-specific enzymes (Marchand, Blanloeil-Oillo, Helm, & Motorin, 2016). There are also methyltransferases that are involved in the formation of 2′-*O*-methylation marks, as in the case of m^6^Am by PCIF1 (Akichika et al., 2019; Boulias et al., 2019; Sendinc et al., 2019).

While the existence of 2′-*O*-methyl modifications has been documented since 1960s, much of the function of several 2′-*O*-methyl modifications still remains unknown, though studies of 2′-*O*-methylation have garnered useful insights that may be helpful in elucidating future discoveries involving 2′-*O*-methylated nucleotides (reviewed in Ayadi, Galvanin, Pichot, Marchand, & Motorin, 2019; Baskin & Dekker, 1967). The presence of 2′-*O*-methyl marks results in degradation resistance upon exposure to alkaline solutions, the disruption of protein-binding activities, and disrupts RNA–DNA duplex formation to limiting off-target effects of siRNAs (Ayadi et al., 2019; Iribe et al., 2017). In eukaryotes, 2′-*O*-methyl modifications are essential for splicing reactions by the major spliceosome (Y.-T. Yu, Shu, & Steitz, 1998). ac^4^Cm and m^2,2^Gm have been shown to have additive effects in stabilizing tRNA structure and conformation at elevated temperatures in archaeal hyperthermophiles, a trend seen with Nm marks overall in 16S rRNA in *T. thermophilus* and possibly other thermophiles (Guymon, Pomerantz, Crain, & McCloskey, 2006; Kawai et al., 1991). In humans, self- and non-self-identification of mRNAs is dependent upon the presence or absence, respectively, of 2′-*O*-methyl marks and elicit an interferon-induced response during a human coronavirus infection and potentially for other viral infections (Abbas et al., 2017). Moreover, knockdown of fibrillarin resulted in a corresponding decrease in ribosome 2′-*O*-methylation and a corresponding increase in translational infidelity (Erales et al., 2017). Colorectal cancer, AML, and cancers that originate due to mutation or deletion of *tp53* may form due to a knockdown or a knockout of fibrillarin (Ayadi et al., 2019; Erales et al., 2017; Sharma, Marchand, Motorin, & Lafontaine, 2017; Zhou et al., 2017). While these modifications have been known for over 50 years, further advances in research on 2′-*O*-methyl marks may elucidate previously unknown biological functions.

## 9 | CONSIDERATIONS OF MODIFIED RIBONUCLEOSIDES AND EVIDENCE FOR THE EXISTENCE OF ADDITIONAL MODIFIED RIBONUCLEOSIDES

### 9.1 | Caution must be exercised for identification of modified ribonucleosides

Historically, several modifications or studies on modifications have demonstrated that caution must be used when interpreting data on RNA modifications, as noted by these nine examples: (a) Initial efforts to map A-to-I modifications across the human transcriptome (M. Li et al., 2011) were found to be filled with false-positive results (Kleinman & Majewski, 2012; W. Lin, Piskol, Tan, & Li, 2012; Pickrell, Gilad, & Pritchard, 2012; Piskol, Peng, Wang, & Li, 2013; Schrider, Gout, & Hahn, 2011). Subsequent research endeavors have been conducted to report more accurately instances of A-to-I modified RNAs (Bahn et al., 2012; Kleinman, Adoue, & Majewski, 2012; Peng et al., 2012; Ramaswami et al., 2012; Ramaswami & Li, 2014). (b) Likewise, failures in correcting for duplications, misannotations, mismapping, SNPs, sequencing errors, and incorporation of nontemplate nucleotides from reverse transcriptase in postsequencing analysis proved detrimental to studies on m^1^A modifications and led to some confusion over which dataset(s) or conditions were accurate (Schwartz, 2018). (c) Prior work indicated that when ms^2^t^6^A was exposed to Tris, an in vivo artifact formed that was a higher ms^2^t^6^A-related compound in mass spectrometry analyses. This artifact was later determined to be ms^2^ct^6^A (Kang et al., 2017; Krog et al., 2011). (d) Early studies on m^6^A involved use of an anti-m^6^A antibody that could also detect m^6^Am in RNA (Linder et al., 2015). m^6^Am marks were localized to being immediately adjacent to the m^7^G cap, although m^6^Am marks found in mRNAs and ncRNAs were misannotated to be m^6^A marks. These misannotated m^6^Am marks were localized to alternative transcription start sites in mRNA isoforms (Akichika et al., 2019; Boulias et al., 2019; Linder et al., 2015; Sendinc et al., 2019; Hanxiao Sun, Zhang, Li, Bai, & Yi, 2019). (e) Peroxywybutosine, o_2_yW, has only been isolated once in tRNA^Phe^ from rat liver extracts despite a separate lab attempting to replicate the o_2_yW isolation, leaving its biological relevance in question (Kasai, Yamaizumi, Kuchino, & Nishimura, 1979). (f) While 5-methyldihydrouridine (m^5^D) was initially identified in the chromosomal RNA of rat ascites tumor cells, a later paper found no evidence of the modification in mouse ascites (Jacobson & Bonner, 1968; Tolstoshev & Wells, 1973). (g) ms^2^m^6^A is another modification with conflicting evidence of existence. There is no manuscript that describes the existence of the modification and it is only referred to as a preliminary modification in The RNA Modification Database. However, it is listed as being present in the majority of tRNAs, in addition to m^6^A (Cantara et al., 2011; Kierzek & Kierzek, 2003). (h) Separately, in the production of mnm^5^se^2^U and cmnm^5^se^2^U, it has been speculated that formation of 5-aminomethyl-2-geranylthiouridine (nm^5^ges^2^U) and 5-aminomethyl-2-selenouridine (nm^5^se^2^U) can occur due to the possibility of decarboxylation of cmnm^5^ges^2^U and cmnm^5^se^2^U. The conversion from cmnm^5^ges^2^U and cmnm^5^se^2^U into nm^5^ges^2^U and nm^5^se^2^U is consistent with prior decarboxylation events with other precursors in this synthesis cascade (C. Zheng, Black, et al., 2017). (i) Historically, in vitro analysis of the effects of modifications on RNAs has been conducted through direct incorporation of the desired modification via synthetic means (reviewed in Dellafiore, Montserrat, & Iribarren, 2016; A. J. Meyer et al., 2015). However, through adjustments of SELEX protocols (reviewed in Dellafiore et al., 2016) or by guided gain-of-function mutations to T7 RNA polymerase (A. J. Meyer et al., 2015), some modified ribonucleotides (e.g., Nm and other 2′-containing modifications) can be incorporated. Though all ribonucleoside modifications contain the potential for newly discovered and exciting biology, they must be carefully vetted.

### 9.2 | Modified ribonucleosides have therapeutic applications

Several strategies involving the use of modified ribonucleosides or their chemical analogs have been exploited in the treatment of human disease. Examples include ribavirin, acyclovir, valacyclovir, and other ribonucleoside analogs in the treatment of viral infections or the use of cytarabine in the treatment of cancer (Seley-Radtke & Yates, 2018). Another example is the use of modified ribonucleobases when transfecting RNAs to increase their stability, to increase their lifetime in complex biological systems that include RNases, or to increase rates of translation (Andries et al., 2015; Karikó, Buckstein, Ni, & Weissman, 2005; Orellana et al., 2017). One type of modification strategy incorporates m^1^Ψ into transfected mRNAs to increase RNA stability and translation rates, consequently enhancing protein expression (Andries et al., 2015). m^1^Ψ and other modifications such as m^6^A, m^5^U, s^2^U, and Ψ reduce immunogenicity of mRNA (Karikó et al., 2005). While a combination of Ψ and m^5^U modifications in transfected RNAs has been successfully used for therapeutic applications in preclinical research, further studies show that m^1^Ψ introduced into mRNA alone or combined with m^5^U have increased rates of mRNA stabilization than mRNAs carrying only Ψ (Andries et al., 2015; Kormann et al., 2011; Miki et al., 2015; Luigi Warren et al., 2010; Wroblewska et al., 2015; Zangi et al., 2013). Incorporation of m^1^Ψ or m^1^Ψ/m^5^C into mRNA results in enhanced translation, lower cytotoxicity, and diminished activation of the intracellular innate immune response in comparison with Ψ or Ψ/m^5^C (Andries et al., 2015). Recent strategies of chemically modifying RNAs have been applied to antisense oligonucleotides or to miRNA derivatives to enhance their therapeutic potential and have even resulted in a cure for diseases such as spinal muscular atrophy or transthyretin-mediated amyloidosis (Adams et al., 2018; Bennett, Krainer, & Cleveland, 2019; Orellana et al., 2017). However, as with all therapeutics, caution must be exercised in the design of RNA-based therapeutics, as immune responses to RNA and normal RNases present in humans can hamper or negate these synthetic RNAs (A.-M. Yu, Jian, Yu, &Tu, 2019).

### 9.3 | Previously undiscovered ribonucleosides may exist

2′-*O*-ribosyluridine may exist, as a part of this previously unknown ribonucleoside was detected in the small molecule Hellecaucaside A, which is found in the hellebore plant *Helleborus caucasicus* or Lenten Rose (Kulikova, Muradova, Drenichev, & Mikhailov, 2015; Sylla et al., 2014). 5-Carboxycytidine (ca^5^C) may exist, as the DNA form of this nucleoside exists and the ribonucleoside form of ca^5^C can be made under in vitro conditions using the TET1 protein to catalyze the conversion of f^5^C to ca^5^C (Basanta-Sanchez et al., 2017). However, despite searches for ca^5^C, it cannot be found in vivo at present (Nakano et al., 2016). While 5-isopentenylaminomethyluridine (inm^5^U) and 5-isopentenylaminomethyl-2-thiouridine (inm^5^s^2^U) have been thought to exist in the tRNA of *Thermodesulfobacterium commune*, these two modifications may be a source of interest, due to the modifications using the 2-thiouridine precursor (Cantara et al., 2011; Limbach et al., 1994). Separately, *N*^6^-hydroperoxymethyladenosine (oxm^6^A) was discovered in an in vitro chemical reaction when m^6^A was exposed to bicarbonate-activated peroxide (J. Wu et al., 2015). Some or all of the new technologies mentioned above could possibly be applied in finding these modifications and others, thereby elucidating previously unknown aspects of biology that may also have medical significance.

## 10 | METHODS OF DETECTING MODIFIED RIBONUCLEOSIDES

### 10.1 | Mass spectrometry-based methods of detecting modified ribonucleosides

Ongoing improvements of well-established technologies and the development of new technology, research tools, and protocols may yield newfound modifications. For example, using mass spectrometry to analyze RNAs has been prevalent for over 20 years, as this method is able to directly distinguish all known modifications with the exception of Ψ-containing modifications (Wetzel & Limbach, 2016). However, new technological breakthroughs to mass spectrometry may further elucidate novel modifications in a high-throughput fashion or at the attomolar level that is consistent with small amounts of cellular RNA from individual cells (Basanta-Sanchez, Temple, Ansari, D’Amico, & Agris, 2016; Jora et al., 2019). For example, conducting LC–MS on *E. coli* tRNAs resulted in the discovery of msms^2^i^6^A and potentially 36 other modifications that could have previously gone undetected (Dal Magro et al., 2018). Additional LC–MS analysis could also yield any of the 143 known modifications in other organisms that were not known to contain them.

In addition to finding RNA modifications in total RNA, sequence-specific detection of RNA modifications can be performed with LC–MS (Addepalli, Venus, Thakur, & Limbach, 2017; Björkbom et al., 2015; Jora et al., 2019). Historically and in current research, finding sequence-specific locations of RNA modifications relies upon the purification of a specific RNA and subsequent partial enzymatic or chemical degradation of RNA into sequencing ladders (Addepalli et al., 2017; Björkbom et al., 2015; N. Zhang, Shi, et al., 2019). Recently, multiple RNases were used to generate unique, overlapping digestion products, which has bypassed the need for the RNA to be highly purified (Thakur, Estevez, Lobue, Limbach, & Addepalli, 2020). Because LC–MS can be limited by the RNase cleavage site, novel RNases that have different substrate specificities can be helpful in sequence-specific detection of modifications (Addepalli et al., 2017). Novel techniques that adapt mass-retention time ladders or hydrophobic end-labeling for LC–MS have been used to address some of the problems in identifying sequence-specific RNA modifications (Björkbom et al., 2015; N. Zhang, Shi, et al., 2019). Reacting CMCT with mRNAs pooled from human cells increases sensitivity of mass spectrometry experiments to m^5^U, as the introduction of a positively charged quaternary amine group found in CMCT onto uridine allowed for improved ionization efficiency in mass spectrometry experiments (Cheng et al., 2020). This use of CMCT was recently successful in determining m^5^U content in mRNAs from HEK293T, HeLa, HL-7702, and HepG2 human cell lines (Cheng et al., 2020).

Mass spectrometry-based approaches of detecting RNA modifications may also have clinical relevance or may be helpful in elucidating previously unknown aspects of biology. Quantitative measurements of RNA modifications have shown reproducible and predictable changes in tRNA modification states under different cellular stresses (Chan et al., 2010). For example, exposure of *S. cerevisiae* to hydrogen peroxide resulted in an increase of m^5^C in tRNA^Leu(UUG)^, which enhances translation of oxidative stress response genes (reviewed in Huber, Leonardi, Dedon, & Begley, 2019). Analysis of *Plasmodium falciparum* m^6^A levels in mRNA by mass spectrometry showed that overall m^6^A levels increase slightly and proportionally to the amount of time postinfection of human red blood cells (Baumgarten et al., 2019). However, individual m^6^A peaks within mRNA can vary drastically in the entire amount of time that *P. falciparum* are inside red blood cells (Baumgarten et al., 2019). Collectively, with further improvements to both mass spectrometers and reagents, more RNA modifications, their locations in the transcriptome, and their roles in biology and in disease will doubtlessly be discovered.

### 10.2 | RNA sequencing-based methods can detect modified ribonucleosides

Direct sequencing of RNAs poses an exciting opportunity for being able to detect known and unknown modifications in RNA transcripts. Both Oxford Nanopore Technologies and Pacific Biosciences possess sequencing methods that allow for quantification of RNA modifications, such as m^6^A marks, and can likely be expanded to find previously unknown modifications (H. Liu, Begik, et al., 2019; Parker et al., 2020). These sequencing methods directly sequence the RNA transcript, rather than cDNA, which allows for modified nucleotides to be detected. Sequencing methodologies to directly sequence tRNAs have begun to elucidate the content and extent of tRNA modifications, especially in disease contexts (Goodarzi et al., 2016). Presently, the noise in high-throughput sequencing unfortunately leads to false-positive RNA modifications. All proposed modifications should be validated using at least one biochemical assay, such as mass spectrometry or SCARLET (Grozhik & Jaffrey, 2018; N. Liu et al., 2013).

The use of antibodies and high-throughput sequencing has represented a recent boon in finding RNA modifications, as several have already been highlighted in this review (Arango et al., 2018; Dai et al., 2017; Dominissini et al., 2012, 2016; K. D. Meyer et al., 2012). Using antibodies greatly aids in reducing the noise of data derived from either mass spectrometry or through downstream high-throughput sequencing methods, as the RNA pool is enriched with the modified RNA (Grozhik & Jaffrey, 2018). Recent progress to use activated nucleotides of modified ribonucleotides or engineering mutants of RNA-modifying enzymes, especially enzymes that form an enzyme–substrate covalent intermediate, offer an intriguing way to pull out RNA targets (Khoddami & Cairns, 2013; T. S. Smith, Zoltek, & Simon, 2019; Tomkuvienė, Mickutė, Vilkaitis, & Klimašauskas, 2019). Further, use of AlkB to demethylate total RNA from human cells, coupled with comparative methylation analysis, was used to identify methylated small RNAs that were derived from tRNAs (Cozen et al., 2015). High-throughput sequencing will continue to be used in determining the location of the modified RNA and may aid in determining the substrates of specific RNA-modifying proteins. The use of new technology, or improvements on classic techniques, continues to unveil previously unknown modifications.

## 11 | CONCLUSIONS

Since the discovery and characterization of pseudouridine, numerous modifications from all domains of life and all four parent nucleotides have been discovered. Much research has been conducted on modifications in eukaryotes, specifically in humans. For example, the m^6^A modification is intimately involved in oncology, cardiology, and several other fields in human biology. However, there are several instances where the presence of modifications in prokaryotes or in nonhuman organisms remains unexplored. There are RNA classes that are not currently known to contain any modifications (e.g., archaeal mRNAs and ncRNAs in Figure 3). Moreover, there are domain-specific modifications that could be found in other domains of life (e.g., cnm^5^U in archaea only). While tRNAs and rRNAs remain a well-spring of RNA modifications, the use of antibodies in probing for modifications and additional technological advances may find modifications not previously known to exist in RNA classes. Moreover, while modifications such as methylations or hydroxylations occur, more novel and complicated modifications may exist that have not been considered. With the advances of LC–MS and other technologies, perhaps additional glyco- and lipid-ribonucleotide conjugates may be found. While threonine, glycine, lysine, arginine, and taurine can be found attached to ribonucleotides, it is possible that other amino acids could be coupled to pyrimidines or purines. The additional possibilities of thiolation or selenation events elsewhere in modifications may also yield new discoveries. As with all of the modifications, their impact on health is only beginning to be understood.

With the increasing progress of finding modifications and identifying their chemical composition, many outstanding questions remain. What is the limit to the number of chemical modifications that exist? Can the abundance of each modification be fit to a power-law plot that may aid in finding previously unknown modifications, similar to how a power-law plot was used to predict riboswitch classes and abundances (McCown, Corbino, Stav, Sherlock, & Breaker, 2017)? In which RNAs will these modifications appear? Will there be any additional discoveries of previously known ribonucleosides in domains in which they are not currently known to exist (Figure 3)? What is the chemical identity and three-dimensional structure of these modifications? Will they establish direct links to the primordial RNA world (Heuberger, Pal, Del Frate, Topkar, & Szostak, 2015; Larsen, Fahrenbach, Sheng, Pian, & Szostak, 2015; Nelson & Breaker, 2017)? Will they be variations of known modifications, or will these modifications sample chemical space in novel ways? Will these modifications have a direct role in catalysis? Will these modifications underlie disease states or elucidate truly unknown biology? All these exciting questions face this ever-growing field of RNA biology and leave much to be anticipated.

## Supplementary Material

Figure S1

## Figures and Tables

**TABLE 1:** All 143 presently known modifications and their abbreviations

Count	RNA modification	Symbol	New symbol	Original NT
*Adenosine*		A	A	A
1	*N*^6^-methyladenosine or 6-methyladenosine	m^6^A	6A	A
2	*N*^1^-methyladenosine or 1-methyladenosine	m^1^A	01A	A
3	*N*^2^-methyladenosine or 2-methyladenosine	m^2^A	2A	A
4	2′-O-methyladenosine	Am	0A	A
5	2-Methylthio-*N*^6^-methyladenosine	ms^2^m^6^A	621A	A
6	*N*^6^-isopentenyladenosine	i^6^A	61A	A
7	*N*^6^-(*cis*-hydroxyisopentenyl)-adenosine	io^6^A	60A	A
8	2-Methylthio-*N*^6^-isopentenyladenosine	ms^2^i^6^A	2161A	A
9	2-Methylthio-*N*^6^-(cis-hydroxyisopentenyl)-adenosine	ms^2^io^6^A	2160A	A
10	*N*^6^-glycinylcarbamoyladenosine	g^6^A	65A	A
11	*N*^6^-threonylcarbamoyladenosine	t^6^A	62A	A
12	2-Methylthio-*N*^6^-threonylcarbamoyl-adenosine	ms^2^t^6^A	2162A	A
13	*N*^6^-methyl-*N*^6^-threonylcarbamoyladenosine	m^6^t^6^A	662A	A
14	*N*^6^-hydroxynorvalylcarbamoyladenosine	hn^6^A	63A	A
15	2-Methylthio-*N*^6^-hydroxynorvalylcarbamoyladenosine	ms^2^hn^6^A	2163A	A
16	2′-O-ribosyladenosine (phosphate)	Ar(p)	00A	A
17	Inosine	I	9A	A
18	*N*^1^-inosine or 1-methylinosine	m^1^I	19A	A
19	1,2′-O-dimethylinosine	m^1^Im	019A	A
20	*N*^6^,*N*^6^-dimethyladenosine	m^6^_2_A or m^6,6^A	66A	A
21	2′-O-methylinosine	Im	09A	A
22	*N*^6^,2′-O-dimethyladenosine	m^6^Am	06A	A
23	*N*^6^,*N*^6^,2′-O-trimethyladenosine	m^6^_2_Am or m^6,6^Am	066A	A
24	*N*^1^,2′-O-dimethyladenosine	m^1^Am	01A	A
25	*N*^6^-acetyladenosine	ac^6^A	64A	A
26	8-Methyladenosine	m^8^A	8A	A
27	*N*^6^-formyladenosine	f^6^A	67A	A
28	Cyclic *N*^6^-threonylcarbamoyladenosine	ct^6^A	69A	A
29	*N*^6^-hydroxymethyladenosine	hm^6^A	68A	A
30	2,8-Dimethyladenosine	m^2,8^A	28A	A
31	Cyclic 2-methylthio-*N*^6^-threonylcarbamoyladenosine	ms^2^ct^6^A	2164A	A
32	*N*^6^-hydroxythreonylcarbamoyladenosine	ht^6^A	2165A	A
33	2-Methylthiomethylenethio-*N*^6^-isopentenyl- adenosine	msms^2^i^6^A	None	A
*Cytidine*		C	C	C
34	3-Methylcytidine	m^3^C	3C	C
35	5-Methylcytidine	m^5^C	5C	C
36	2′-O-methylcytidine	Cm	0C	C
37	2-Thiocytidine	s^2^C	2C	C
38	*N*^4^-acetylcytidine	ac^4^C	42C	C
39	5-Formylcytidine	f^5^C	71C	C
40	5,2′-O-dimethylcytidine	m^5^Cm	05C	C
41	Lysidine	k^2^C	21C	C
42	*N*^4^-methylcytidine	m^4^C	4C	C
43	4,2′-O-dimethylcytidine	m^4^Cm	04C	C
44	5-Hydroxymethylcytidine	hm^5^C	51C	C
45	5-Formyl-2′-O-methylcytidine	f^5^Cm	071C	C
46	*N*^4^,*N*^4^,2′-O-trimethylcytidine	m^4^_2_Cm or m^4,4^Cm	044C	C
47	Agmatidine	C^+^	20C	C
48	5-Hydroxycytidine	ho^5^C	50C	C
49	*N*^4^-acetyl-2′-O-methylcytidine	ac^4^Cm	042C	C
50	*N*^4^,*N*^4^-dimethylcytidine	m^4,4^C	44C	C
51	2-Methylthiocytidine	ms^2^C	None	C
52	2′-*O*-methyl-5-hydroxymethylcytidine	hm^5^Cm	None	C
*Guanosine*		G	G	G
53	1-Methylguanosine	m^1^G	1G	G
54	*N*^2^-methylguanosine	m^2^G	2G	G
55	7-Methylguanosine	m^7^G	7G	G
56	2′-O-methylguanosine	Gm	0G	G
57	*N*^2^,*N*^2^-dimethylguanosine	m^2,2^G	22G	G
58	*N*^2^-2′-O-dimethylguanosine	m^2^Gm	02G	G
59	*N*^2^,*N*^2^,2′-O-trimethylguanosine	m^2,2^Gm	022G	G
60	2′-O-ribosylguanosine (phosphate)	Gr(p)	00G	G
61	Wybutosine	yW	3483G	G
62	Peroxywybutosine	o_2_yW	34832G	G
63	Hydroxywybutosine	OHyW	34830G	G
64	Undermodified hydroxywybutosine	OHyWx or OHyW*	3470G	G
65	Wyosine	imG	34G	G
66	Methylwyosine	mimG	342G	G
67	Queuosine	Q	10G	G
68	Epoxyqueuosine	oQ	102G	G
69	Galactosyl-queuosine	galQ	104G	G
70	Mannosyl-queuosine	manQ	106G	G
71	Glutamyl-queuosine	gluQ	105G	G
72	Pre-queuosine_0_	preQ_0_	100G	G
73	Pre-queuosine_1_	preQ_1_	101G	G
74	Archaeosine	G^+^	103G	G
75	*N*^2^,7-dimethylguanosine	m^2,7^G	27G	G
76	*N*^2,2^-7-trimethylguanosine	m^2,2,7^G	227G	G
77	1,2′-O-dimethylguanosine	m^1^Gm	01G	G
78	4-Demethylwyosine	imG-14	4G	G
79	Isowyosine	imG2	42G	G
80	*N*^2^,2′-O-7-trimethylguanosine	m^2,7^Gm	027G	G
81	7-Aminocarboxypropylwyosine methyl ester	yW-58	348G	G
82	7-Aminocarboxypropyl-demethylwyosine	yW-86	47G	G
83	7-Aminocarboxypropylwyosine	yW-72	347G	G
84	Methylated undermodified hydroxywybutosine	OHyWy	3480G	G
85	2-Hydroxymethylguanosine	hm^2^G	None	G
*Uridine*		U	U	U
86	Pseudouridine	ψ	9U	U
87	Dihydrouridine	D	8U	U
88	5-Methyluridine, ribosylthymine, or ribothymidine	m^5^U or rT	5U	U
89	2′-O-methyluridine	Um	0U	U
90	5,2′-O-dimethyluridine	m^5^Um or Tm	05U	U
91	1-Methylpseudouridine	m^1^ψ	19U	U
92	2′-O-methylpseudouridine	ψm	09U	U
93	2-Thiouridine	s^2^U	2U	U
94	4-Thiouridine	s^4^U	74U	U
95	2-Thio-2′-O-methyluridine	s^2^Um	02U	U
96	3-(3-Amino-3-carboxypropyl)uridine	acp^3^U	30U	U
97	5-Hydroxyuridine	ho^5^U	50U	U
98	5-Methoxyuridine	mo^5^U	501U	U
99	Uridine 5-oxyacetic acid	cmo^5^U	502U	U
100	Uridine 5-oxyacetic acid methyl ester	mcmo^5^U	503U	U
101	5-Carboxyhydroxymethyluridine	chm^5^U	520U	U
102	5-Carboxyhydroxymethyluridine methyl ester	mchm^5^U	522U	U
103	5-Methoxycarbonylmethyluridine	mcm^5^U	521U	U
104	5-Methoxycarbonylmethyl-2′-O-methyluridine	mcm^5^Um	0521U	U
105	5-Aminomethyl-2-thiouridine	nm^5^s^2^U	2,510U	U
106	5-Methylaminomethyluridine	mnm^5^U	511U	U
107	5-Methylaminomethyl-2-thiouridine	mnm^5^s^2^U	2,511U	U
108	5-Methylaminomethyl-2-selenouridine	mnm^5^se^2^U	20,511U	U
109	5-Carbamoylmethyluridine	ncm^5^U	53U	U
110	5-Carbamoylmethyl-2′-O-methyluridine	ncm^5^Um	053U	U
111	5-Carboxymethylaminomethyluridine	cmnm^5^U	51U	U
112	5-Carboxymethylaminomethyl-2′-O-methyluridine	cmnm^5^Um	051U	U
113	5-Carboxymethylaminomethyl-2-thiouridine	cmnm^5^s^2^U	251U	U
114	3-Methyluridine	m^3^U	3U	U
115	1-Methyl-3(3-amino-3-carboxypropyl) pseudouridine	m^1^acp^3^ψ	1309U	U
116	5-Carboxymethyluridine	cm^5^U	52U	U
117	3,2′-O-dimethyluridine	m^3^Um	03U	U
118	5-Methyldihydrouridine	m^5^D	58U	U
119	3-Methylpseudouridine	m^3^ψ	39U	U
120	5-Taurinomethyluridine	τm^5^U	54U	U
121	5-Taurinomethyl-2-thiouridine	τm^5^s^2^U	254U	U
122	5-(Isopentenylaminomethyl)uridine	inm^5^U	583U	U
123	5-(Isopentenylaminomethyl)-2-thiouridine	inm^5^s^2^U	2583U	U
124	5-(Isopentenylaminomethyl)-2′-O-methyluridine	inm^5^Um	0583U	U
125	5-Cyanomethyluridine	cnm^5^U	55U	U
126	5-(Carboxyhydroxymethyl)-2′-O-methyluridine methyl ester	mchm^5^Um	0522U	U
127	5-Carboxymethylaminomethyl-2-selenouridine	cmnm^5^se^2^U	2051U	U
128	5-Carboxymethylaminomethyl-2-geranylthiouridine	cmnm^5^ges^2^U	2151U	U
129	5-Methylaminomethyl-2-geranylthiouridine	mnm^5^ges^2^U	21511U	U
130	5-Aminomethyl-2-geranylthiouridine	nm^5^ges^2^U	21510U	U
131	5-Methoxycarbonylmethyl-2-thiouridine	mcm^5^s^2^U	2521U	U
132	5-Carbamoylmethyl-2-thiouridine	ncm^5^s^2^U	253U	U
133	3(3-Amino-3-carboxypropyl)-5,6-dihydrouridine	acp^3^D	308U	U
134	5-Aminomethyl-2-selenouridine	nm^5^se^2^U	20510U	U
135	5-Carbamoylhydroxymethyluridine	nchm^5^U	531U	U
136	5-Carboxymethyl-2-thiouridine	cm^5^s^2^U	2540U	U
137	5-Methyl-2-thiouridine	m^5^s^2^U	25U	U
138	2-Geranylthiouridine	ges^2^U	21U	U
139	2-Selenouridine	se^2^U	20U	U
140	5-Aminomethyluridine	nm^5^U	510U	U
141	2′-O-methyluridine 5-oxyacetic acid methyl ester	mcmo^5^Um	0503U	U
142	3-(3-Amino-3-carboxypropyl)pseudouridine	acp^3^ψ	309U	U
143	5-Cyanomethyl-2-thiouridine	cnm^5^s^2^U	None	U

*Note*: The column labeled “RNA modification” lists the modification name. The column labeled “Symbol” lists the traditionally named abbreviations or symbols for the modification. Nonalphanumeric characters seen in abbreviations, such as plus signs, commas, parentheses, asterisks, and other such characters, significantly complicate regular expression scripting in Linux-based environments. To mitigate this problem, the column labeled “New symbol” lists a new set of symbols for these modifications that is easier for regular expressions to use (Boccaletto et al., 2018; Jonkhout et al., 2017). The column labeled “Original NT” indicates which nucleotide (A, C, G, or U) is the ultimate originating nucleotide for the modification.

**TABLE 2:** Modifications at known nucleotide positions in tRNAs

tRNA position	Modification(s)
1	Ψ
4	Am, Cm
8	s^4^U
9	m^1^A, m^1^G, s^4^U
10	m^2^G, m^2,2^G, m^2^Gm, m^2,2^Gm
12	ac^4^C
13	Ψ
15	G^+^, preQ_0_
16	D
17	D, Gm
19	D
20	D,acp^3^U
21	Ψ
26	m^2^G, m^2,2^G, m^2^Gm, m^2,2^Gm
27	Ψ
28	Ψ
31	Ψ
32	Ψ, m^3^C, Cm, s^2^C, s^2^U, ms^2^C
33	s^2^U
34	Ψ, cnm^5^U, ho^5^U, mo^5^U, cmo^5^U, mcmo^5^U, mcmo^5^Um, m^3^U, I, f^5^C, m^5^C, m^3^C, preQ_0_, preQ_1_, Q, oQ, galQ, manQ, gluQ, Cm, s^2^U, s^2^Um, nm^5^s^2^U, mnm^5^s^2^U, cmnm^5^s^2^U, C^+^, k^2^C, ac^4^C, Gm, mcm^5^U, mcm^5^Um, ncm^5^U, ncm^5^Um, τm^5^U, τm^5^s^5^U, cmnm^5^U, cmnm^5^s^5^U, mnm^5^se^2^U, cmnm^5^ges^2^U, mnm^5^ges^2^U, cmnm^5^se^2^U, mnm^5^se^2^U, mnm^5^U, nm^5^U, nchm^5^U, se^2^U, ges^2^U, chm^5^U, mchm^5^U, mcm^5^s^2^U, ncm^5^s^2^U, cm^5^s^2^U
35	Ψ
36	Ψ
37	i^6^A, io^6^A, ms^2^i^6^A, ms^2^io^6^A, t^6^A, g^6^A, ht^6^A, m^6^t^6^A, ct^6^A, ms^2^t^6^A, ms^2^ct^6^A, I, m^1^A, m^1^I, m^2^A, m^1^G, imG-14, imG, imG2, mimG, yW-86, yW-72, yW-58, yW, OHyW*, OHyWy, o_2_yW, OHyW, m^6^A, s^2^C
38	Ψ
39	Ψ, Um
40	m^5^C
46	m^7^G
47	acp^3^U
48	m^5^C
49	m^5^C
52	Ψ
53	Ψ
54	Ψ,m^1^Ψ, m^5^U, m^5^s^2^U
55	Ψ
56	Cm
57	m^1^A, m^1^I, I
58	m^1^A, m^1^I
64	Ar(p), Gr(p)
65	Ψ
66	Ψ
Unknown location	msms^2^i^6^A, ac^6^A, hn^6^A, ms^2^hn^6^A, m^6,6^A, acp^3^D, m^3^Ψ, m^4,4^Cm, m^6^Am, m^6,6^Am, m^1^Am, Im, m^1^Im, m^1^Gm, m^2,7^Gm, m^4^Cm, m^5^Cm, f^5^Cm, ac^4^Cm, mchm^5^Um, mcmo^5^Um, inm^5^Um

*Note*: Specific positions within tRNAs, labeled “tRNA position,” indicate which nucleotide is modified (related to Figure 4). The column labeled “Modification(s)” indicates which modification(s) can be found at a given nucleotide position. All modifications are abbreviated as indicated in “Symbol” column of Table 1.
